# Tissue Culture in Ornamentals: Cultivation Factors, Propagation Techniques, and Its Application

**DOI:** 10.3390/plants11233208

**Published:** 2022-11-23

**Authors:** Hasan Mehbub, Ayasha Akter, Mst. Arjina Akter, Mohammad Shamim Hasan Mandal, Md. Ashraful Hoque, Monika Tuleja, Hasan Mehraj

**Affiliations:** 1 The United Graduate School of Agricultural Science, Ehime University, Matsuyama 790-8556, Japan; 2 Department of Horticulture, Bangladesh Agricultural University, Mymensingh 2202, Bangladesh; 3 Department of Plant Pathology, Bangladesh Agricultural University, Mymensingh 2202, Bangladesh; 4 Graduate School of Agricultural Science, Kobe University, Rokkodai, Nada-ku, Kobe 657-8501, Japan; 5 Graduate School of Advanced Science and Engineering, Hiroshima University, Hiroshima 739-8511, Japan; 6 Department of Plant Cytology and Embryology, Institute of Botany, Faculty of Biology, Jagiellonian University, Gronostajowa 9, 30-387 Krakow, Poland

**Keywords:** in vitro, callus, somatic embryogenesis, hybridization, protoplast fusion, protocorm-like body, synthetic seeds, epigenetic variation

## Abstract

Ornamentals come in a variety of shapes, sizes, and colors to suit a wide range of climates, landscapes, and gardening needs. Compared to demand, a shortage of plant materials and diversity force the search for solutions for their constant acquisition and improvement to increase their commercial value, respectively. In vitro cultures are a suitable solution to meet expectations using callus culture, somatic embryogenesis, protoplast culture, and the organogenesis of protocorm-like bodies; many of these techniques are commercially practiced. Factors such as culture media, explants, carbohydrates, plant growth regulators, and light are associated with the success of in vitro propagation. Techniques, especially embryo rescue and somatic hybridization, are widely used to improve ornamentals. The development of synthetic seed allows season-independent seed production and preservation in the long term. Despite the advantages of propagation and the improvement of ornamentals, many barriers still need to be resolved. In contrast to propagation and crop developmental studies, there is also a high scope for molecular studies, especially epigenetic changes caused by plant tissue culture of ornamentals. In this review, we have accumulated and discussed an overall update on cultivation factors, propagation techniques in ornamental plant tissue culture, in vitro plant improvement techniques, and future perspectives.

## 1. Introduction

Ornamental horticulture, flowering, and landscape horticulture are economically viable industries in the agricultural sector. It deals with producing and commercializing flowers, flowering plants, foliage plants, and landscape plants; plants used in ornamental horticulture are collectively called ornamentals. Plant tissue culture, including micropropagation, is the most applicable plant propagation technique in ornamentals. It allows the production of several exact genetic copies from small pieces of plant tissue (known as explants), and the propagation of uniform, season-independent, and seed-borne diseases free seedlings is an additional advantage [1]. Thus, micropropagation techniques are frequently used in the commercial production of seedlings in diversified plant species. Explants are usually cultured in a nutrient-supplemented medium under sterile conditions. White’s medium was the first chemically defined nutrient medium [2,3,4,5]. Afterward, Murashige and Skoog (1962) developed a new nutrient supplemented medium, which is known as Murashige–Skoog (MS) medium [6], and it is the most used nutrient supplemented medium in the world for plant tissue culture. Other nutrient-supplemented media, such as White (WH) medium [7], Linsmaier and Skoog (LS) medium [8], Gamborg (B5) medium [9], Nitsch and Nitsch (NN) medium [10], etc., are also widely accepted. These nutrient supplement media are the basal media that usually contain major salts (plant macronutrients), minor salts (plant micronutrients), vitamins, and organic compounds. Solidifying agents are used in the basal medium to support the plantlets in micropropagation and, to some extent, in liquid culture medium. Different kinds of agars—phytagel, gelrite, gellan gum, etc.—are used to solidify the nutrient supplement culture media for plant tissue culture that are available in the commercial market. The pH of the basal nutrient supplement media keeps changing during preparation, which is required to adjust before autoclaving. The pH in the media is considered a dynamic variable for in vitro plant growth and development. MS medium is the most used medium in vitro, and manipulation of MS medium and culture conditions according to the plant—specific requirements are also practiced [11,12]. The success of plant tissue culture techniques largely depends on sources of carbon, plant growth regulators (PGRs), culture environment, lights, genotype, type of explant, etc. The key tool for the success of plant tissue culture technology greatly relies on the proper culture media composition and their culture condition because of plant—specific response. Other than propagation, tissue culture technology has been used for plant improvement, somatic hybrid development, synthetic seed production, and ploidy manipulation. We reviewed the research findings of plant tissue culture technologies for ornamental plant propagation, cultivation factors, and their application in ornamentals from a future perspective.

## 2. In Vitro Cultivation Factors

### 2.1. Carbohydrate Supplements as Carbon Sources in Culture Media

Plant tissues, cells, and organs usually go through either heterotrophic or mixotrophic conditions in vitro. Heterotrophic conditions are the primary obstacle to in vitro plant growth and development where cultured tissue or cell or organ can only synthesize their required nutrients from the basal media, and mixotrophy conditions where plants depend on heterotrophy and can produce food by photosynthesis as well [13]. Plants need exogenous carbohydrates in both phases for proper growth and development due to their morphogenic effect on nutritional value, osmotic potential, and cell division [13,14]. The addition of exogenous carbohydrates can easily supply energy to explants when explants are not ready for photosynthesis. Even though plants become ready for photosynthesis, they need exogenous carbohydrate supplements because of their lower photosynthesis efficiency than in vivo conditions [15]. Exogenous carbohydrate supplements assist plant embryos in increasing cell division by encouraging cell expansion and reserve accumulation [16]. Many forms of carbon sources are available in commercial markets, such as sucrose, glucose, fructose, maltose, trehalose, lactose, galactose, sorbitol, etc. The specific sources and requirements vary according to the plant species, stages, tissues for explants, culture period, culture environment, etc. [13]. Sucrose is superior and cheaper than other carbon sources, which ensures favorable effects on in vitro plant growth [17,18,19,20]. In plants, phloem sap contains sucrose to control plant growth and developmental processes [21], while sucrose is highly soluble in water, acts as a molecule transporter, and is transported by the plasma membrane [22,23]. Therefore, plants efficiently utilize sucrose for their carbon requirements during the in vitro heterotrophic and mixotrophic phases. About 2–5% sucrose concentrations are generally used in plant tissue culture of ornamentals [24]. Depending on the plant species and culture conditions, other carbon sources showed more efficiency than sucrose. For example, the wishbone flower (*Torenia fournieri*) extended twice its vegetative culture period in a trehalose-based culture medium over a sucrose-based medium without alteration in plant viability [25]. Trehalose was found to be equally or sometimes more effective than sucrose for the propagation of protocorm-like bodies (PLBs) in *Phalaenopsis* and *Doritaenopsos* orchids [20,26]. Glucose stimulates the in vitro shoot and root growth of chrysanthemum, while its intermediate product, fructose, slowly affects in vitro plant growth and development; however, its efficiency varies according to plant species and culture conditions [27,28]. On the other hand, slower hydrolysis (20 times slower than sucrose) of maltose is the main oblige [29]. Plants take a long time to absorb and metabolize maltose, and the requirement is sometimes twice that of sucrose [29]. From the above discussion, it is clear that exogenous carbohydrate supplements are crucial for in vitro plant growth and development. Exogenous carbohydrate concentration is also varied, and concentrations over a threshold level could be toxic, hamper photosynthesis, and inhibit in vitro plant growth [30,31,32].

### 2.2. Plant Growth Regulators, Inhibitors, and Elicitors in Culture Media

The application of PGRs in basal media accelerates the induction of a new plant from a cell or tissue. Auxins (Au), gibberellins (GA), cytokinins (CK), abscisic acid (ABA), and ethylene (ET) are the five groups of PGRs; Au and CK are widely used, while GA, ABA, and ET are less used for vitro micropropagation of ornamentals [33]. The in vitro propagation of plants is significantly influenced by the addition of auxin to culture media. Auxin triggers cell division and leaf initiation before lateral root initiation [34,35,36], and it is crucial for the formation of meristems [37]. In culture media with auxin, the cells of the explant rapidly undergo cell division to form calli [38] and start to develop shoots and/or roots from the calli [39]. A proper concentration of auxin can assist in initiating plant roots; application of exogenous auxin can stimulate auxin-triggered pathways and GA biosynthesis; meanwhile, it can suppress ABA and ET biosynthesis [40]. The naturally occurring auxins (indole-3-acetic acid (IAA), indole-3-butyric acid (IBA), indole-3-propionic acid (IPA), 4-chloroindole-3-acetic acid, phenylacetic acid, etc.) and synthetic auxins (4-chlorophenoxy acetic acid (4-CPA), dicamba, picloram, etc.) are used in plant tissue culture [41,42]. Cytokinin is naturally found in all plant tissues; however, it is enriched in the root tip, shoot apex, and immature seeds [43]. In vitro micropropagation, cytokinin stimulates cell division and is usually used to initiate the growth and proliferation of buds and shoots and slow down root formation [24]. 6-benzyloaminopurine (BAP), 6-(γ,γ-dimethylallylamino)purine (2iP), kinetin, zeatin, and thidiazuron-N-phenyl-N-1,2,3 thiadiazol-5-ylurea (TDZ) are commonly used cytokinins in plant tissue culture. A combination of Au–CK, different combinations of their concentrations, is frequently used in micropropagation, and the effect of both Au and CK depends on their relative concentrations. A culture medium with high-cytokinin and low-auxin causes shoot initiation, while a high-auxin and low-cytokinin medium causes root initiation [44]. Gibberellic acid (GA_3_) is the most used gibberellin, which is used to accelerate in vitro plant growth, while the function of ABA can be stimulatory or inhibitory depending on different factors such as media ingredients, light intensity and quality, or plant species [24]. We have summarized 200 research articles belonging to 52 ornamentals studied for the effective PGR concentration and/or effective PGR combinations with their suitable concentrations (Supplementary Table S1).

Different growth retardants, such as paclobutrazol, daminozide, chlorocholine chloride (CCC), ancymidol, etc., can be beneficial for plants propagated in vitro media (Supplementary Table S2). Growth retardants act as anti-gibberellin in sucrose supplemented medium; the addition of growth retardant in culture media activates adenosine diphosphate-glucose pyrophosphorylase (ADGPase) and UDP glucose pyrophosphorylase (UDPGPase), which promotes starch synthesis [45,46]. Similar to tuberization in potatoes, CCC showed a prompting effect on in vitro PLB regeneration in *Phalaenopsis* orchid [26,46]. In vitro treatment of growth retardants, e.g., paclobutrazol, has an additional advantage in increasing in vitro to ex vitro transfer efficiency [47,48]. The positive regulation of growth retardants in micropropagation cannot be ignored. Elicitors, biotic and abiotic, have been widely used for triggering secondary metabolites in plant tissue culture [49]. Chitosan (Ch), aminolevulinic acid (ALA), alginate (Ag), *N*-acetylglucosamine (NAG), salicylic acid (SA), hyaluronic acid (HA), silver nitrate (AgNO_3_), jasmonic acid (JA), methyl jasmonate (MeJA), phloroglucinol (PG), pectin, casein hydrolysate, and yeast extracts are some common elicitors [50]. Like growth retardants, elicitors are also beneficial for micropropagation. For example, MeJA, ALA, AgNO_3_, HA, Ch, and PG trigger PLBs, root shoots, and flower organogenesis in ornamentals (Supplementary Table S2). In tulips, MeJA has been applied in a combination of different polyamines for efficient bulb formation, and MeJA-polyamine combinations significantly enhanced bulb formation [51]. An oxidizing biocicide, chlorine dioxide (ClO_2_), has been used in the culture media for in vitro plant regeneration in chrysanthemum and gerbera, and it accelerates plant shoot and root regeneration [52,53]. Additives, organic and synthetic, can also influence in vitro plant growth and development (Supplementary Table S3).

### 2.3. Light-Emitting Diodes over Conventional Light

**Figure 1:**
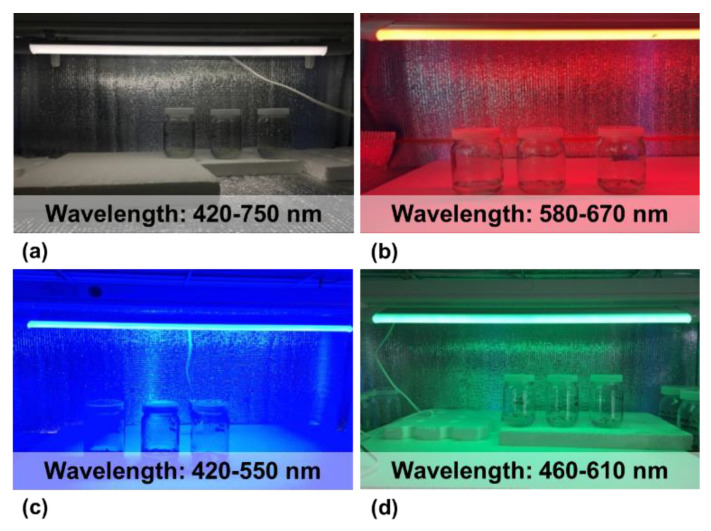
The application of monochromatic white (**a**), red (**b**), blue (**c**), and green (**d**) LEDs with specific wavelengths (white LED; 420–750 nm, red LED; 580–670 nm, blue LED; 420–550 nm, and green LED; 460–610 nm) for in vitro PLB proliferation.

In vitro culture conditions can be manipulated by altering the light color and intensity for effective morphogenesis or organogenesis. Plants can respond well to a wide spectrum of light in terms of plant growth and development with wavelengths of <400 nm (UV radiation), 400~700 nm (visible), and 700~800 nm (far-red) [54]. White fluorescent light with 350~750 nm wavelengths spectral emission is the conventional light source in vitro culture; however, the consumption of high electricity, high radiant heat, and uneven radiation are the major disadvantages [55]. Monochromatic light-emitting diodes (LEDs) with a specific range of wavelengths are widely used for in vitro plant propagation (Figure 1). 

LEDs are the most efficient over white fluorescent light, which overcomes the stated disadvantages [56,57]. Red LED showed efficiency for callus proliferation, PLB organogenesis, PLB proliferation, shoot induction, shoot multiplications, and plantlet regeneration in different ornamentals, such as orchids, gerbera, chrysanthemum (cv. Kitam Cheonsu), anthurium (cv. Violeta and Pink Lady), heliconia, peace lily, giant protea, and hosta (Supplementary Table S4). Far red was efficient for the plant growth of chrysanthemum (cv. Ellen) (Supplementary Table S4). A higher percentage of red LED with a lower percentage of blue LED is suitable for the PLBs and plantlet regeneration of *Phalaenopsis*, *Rosa × kordesii*, chrysanthemum (cv. Ellen), gerbera, anthurium, heliconia, peony, and spurflower, while some other ratios of red and blue LED mixture were found to be effective in some ornamentals (Supplementary Table S4).

A mixture of red and blue LEDs, compared with red LED alone, enhanced both plant growth and development by increasing the net photosynthesis in *Cymbidium* [58,59] because the spectral energy distribution of red and blue light coincides with that of chlorophyll absorption [60]. Red and blue LED combinations were reported as effective for the growth and development of PLBs in *Cymbidium*, *Doritaenopsis*, *Phalaenopsis*, and *Calanthe* [26,57] (Supplementary Table S4). Blue LED increases the shoot formation of PLB cultures in *Dendrobium officinale* and *D. kingianum* [61,62], while PLBs cultured under red and blue LED showed the lowest and highest, respectively, in vitro differentiation rates on *Oncidium* and *D. officinale* [61,63]. Very little information is available on the effect of green LED on in vitro micropropagation of ornamentals. In recent studies, it was found that green LED increased PLB regeneration in *Dendrobium* [31], and *Cymbidium* [64]; however, PLB generation was more efficient under green LED when culture media had anti-auxin, PCIB (*p*-Chlorophenoxyisobutyric acid, an anti-auxin), in *D. okinawense* [31]. Additionally, yellow and orange light spectra have also been reported, respectively, in PLB, shoot, and plantlet regeneration of *Dendrobium* and seed germination and rhizoid development of *Bletilla ochracea* (Supplementary Table S4). These results suggest the requirement for a diverse range of light spectra for in vitro micropropagation, which largely depends on plant species and culture media supplements (Supplementary Table S4; Supplementary Table S5). 

## 3. Standard Techniques Involved in Plantlet Generation In Vitro

### 3.1. Callus Culture

In the early 20th century, callus formation and its ability to generate independent life were first noticed [65,66]. The callus is a mass of loosely packed parenchymatous cells with various degrees of differentiation, which is raised from the in vitro proliferating cells of plant tissue in response to biotic and abiotic stimuli. It is similar to non-differentiated meristematic cells but different from differentiated plant cells. Depending on the accumulated compounds, calli may be pale brown, creamish yellow, greenish, or colorless. The callus is cytologically diverse in shape and type of cells and is genetically heterogeneous. Under the influence of selected phytohormones, a certain pool of parenchymal callus cells is dedifferentiated and has dividing activity. Calli lack chloroplasts for photosynthesis and have a small vacuole, and their culture can generate new plants. Callus can be inducted from any plant part, such as seeds, leaves, stem, root, flowers, etc.; successful callus induction depends on plant species, explant used for the callus inductions, culture media, PGR supplements in culture media, and growth conditions [67]. Two major PGR groups, auxin and cytokinin, are largely used for callus induction [67]. Some plant species induce callus in day–night conditions, while some need entirely night conditions. Callus induction gives an idea of the potentiality of in vitro regeneration of any plant species, while it can also be a good source of materials for other in vitro culture techniques and can be used for long-term preservation [67]. Callus has been used for the successful plant regeneration and genetic modification of different ornamental plant species [68].

### 3.2. Protoplast Culture

**Figure 2:**
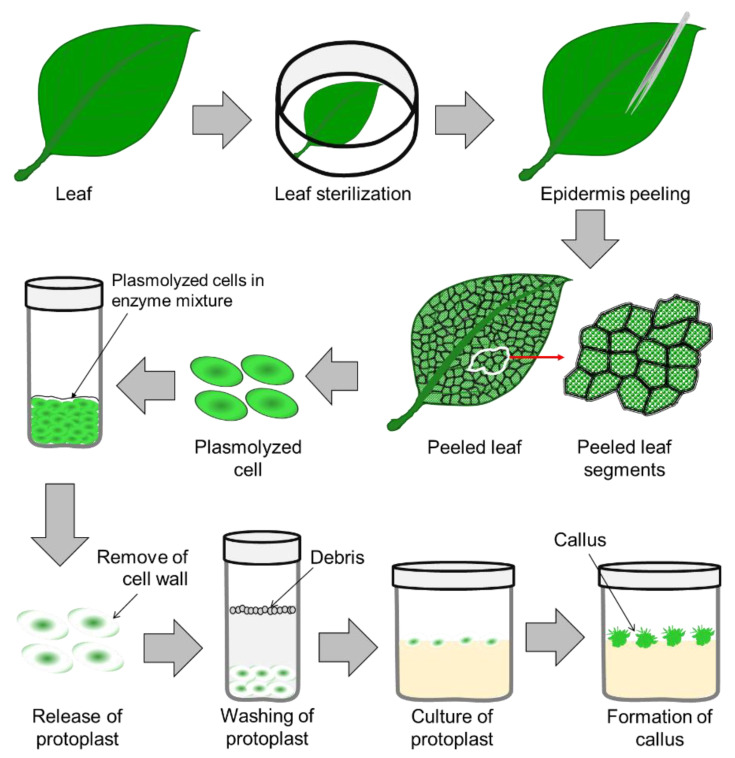
A detailed scheme of protoplast isolation and establishment of an in vitro protoplast culture.

Protoplast culture is used for plantlet regeneration (process illustrated in Figure 2), and protoplast fusion is used for crop improvement, which is known as somatic hybridization (details in Section 4.2) [69]. The nature of the explant tissue and the thickness of the cell wall play an important role in high-efficiency protoplast isolation, which is a critical stage in the process of seedling regeneration or somatic hybridization. However, protoplasts were successfully isolated and cultured in different ornamentals, such as *Dendrobium* [70], lily [71], rose [72], chrysanthemum [73], petunia [74], carnation [75], coneflower [76], geraniums [77,78], Persian silk tree [79], etc. Pre-plasmolyzing the explant tissue with osmotic stabilizers, such as mannitol and sorbitol, before enzyme treatment is effective for protoplast isolation in most plant tissue [80].

Sugar concentration is another important factor for high-yield protoplasts, and the effective sugar concentration ranges from 0.3 to 0.8 M in ornamentals [69,74,76,81,82,83,84]. Factors such as the concentration of enzyme, digestion period, pH of the enzyme solution, temperature, and agitation during incubation are also important for protoplast isolation in ornamentals [69,73,74,79,83,85,86]. In orchids, the first protoplasts were isolated in 1978 [87,88], while few studies reported colony formation [89,90,91,92,93]. After successful protoplast isolation, there are some challenges to plantlet regeneration from an isolated protoplast. Types of culture medium, culture medium components, strength of the culture medium, carbon sources, pH of the culture medium, supplements of the culture medium, PGRs, and culture conditions have been proven to be vital factors for plantlet generations from protoplasts [69]. Considering these factors and despite these limitations, plantlets have been generated successfully in several ornamental plant species [69,73,74,78,94,95].

### 3.3. Somatic Embryogenesis

**Figure 3:**
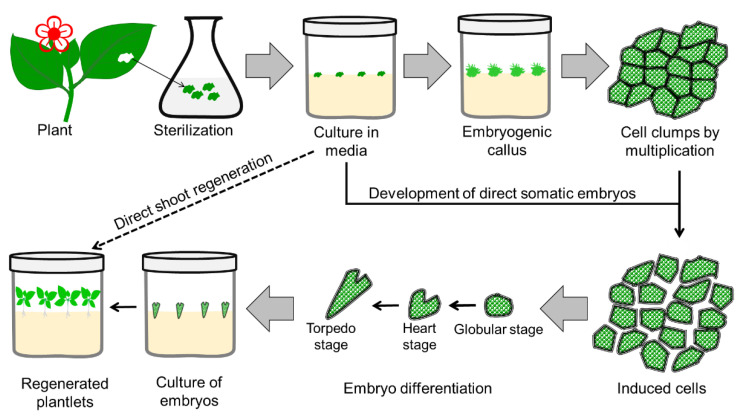
Diagrammatic presentation of the steps involved in somatic embryogenesis for mass propagation in plants.

An alternative to root and shoot regeneration from the callus, regeneration of the whole plant from the plant cell throughout embryo formation, was identified in 1958 [96,97]. The development of an embryo or plant from the vegetative/somatic cell is known as somatic embryogenesis [98]. The procedure for somatic embryogenesis is illustrated in Figure 3. Somatic embryogenesis is considered more efficient than other propagation techniques. which guarantees variability. It produces identical genotypes differing from zygotic embryos, which guarantees variability. The bipolar structure of a somatic embryo consists of apical (known as plumule) and basal meristem regions (known as radicles), which are responsible for shoot and root formation, respectively [99]. Cytological and histological studies have confirmed that PLBs (details in Section 3.4) are also somatic embryos [99]. Morphogenesis or regeneration of PLBs can be initiated by direct or indirect embryogenesis. Organogenesis of PLB avoiding the callus phase is known as direct embryogenesis, and PLB generated from the callus (an intermediate phase) is known as indirect embryogenesis [99].

In somatic embryogenesis, the morphogenic response varies on factors like explants, PGRs, hormones, concentrations of PGRs or hormones, light, etc. [99,100,101,102]. Plantlet regeneration by somatic embryogenesis has been reported in many genera of orchids; for example—*Cymbidium* [103,104,105,106,107,108], *Phalaenopsis* [108,109,110,111,112,113,114,115], *Oncidium* [28,116,117,118,119,120], *Dendrobium* [121,122,123,124], *Rhynchostylis* [125], *Renanthera* [126], *Paphiopedilum* [127,128], *Malaxis* [129,130], *Epipactis veratrifolia* [131], *Spathoglottis plicata* [132], *Geodorum densiflorum* [133], *Anoectochilus elatus* [134], and *Nothodoritis zhejiangensis* [135]. In addition to orchids, it has also been reported in diverse ornamentals, such as rose [136], *Rosa × damascena* [137], chrysanthemum [138,139], lilies [140,141,142,143,144,145,146], jasmine [147], lisianthus [148,149,150,151], carnation [152], *Camellia* [153,154,155,156,157], *Cineraria* [158], coneflower [159,160], *Crocus* [161,162,163], *Clematis* [164,165,166]; Sawara cypress [167], cyclamen [168], bellflower [169], passion flowers [170], perennial daisy and false daisy [171,172]; tulip [173], periwinkle [174], peony [175,176], anthurium [177,178,179,180,181], gentian [182,183,184,185], *Exacum trinervium* [186], gloriosa [187,188], amaryllis [189], phlox [190], *Centaurium erythraea* [191], *Lachenalia viridiflora* [192], pine [193,194,195,196], Japanese black pine [197], agave [198,199,200,201], and hosta [202].

### 3.4. Protocorm-like Body

In *Cymbidium* orchid, protocorm-like bodies (PLBs) were noticed for the first time during the shoot-tip culture by Morel (1960) [203]. Protocorms are small spherical tuber-like structures formed in a germinating seed; protocorm-like structures with similar characteristics generated from somatic cells in tissue culture techniques are known as PLBs [204,205]. PLBs are induced directly from explants and/or indirectly from calluses [206], and the formation, regeneration, and proliferation of PLBs are among the most efficient techniques of micropropagation, especially for clonal propagation of orchids [207]. Meristemoids in callus cells initiate polarized growth, and continuous cell division causes the shoot pole (for shoot initiation) and the base pole (for root initiation) of a protocorm-like body (PLB) [127,204,208]. The induction of PLBs has several advantages over typical shoot and plantlet regeneration, such as a higher rate of multiplications, long-term preservation, easy differentiation into shoots, generations of secondary PLBs, etc. The success of efficient PLB induction, regeneration, and proliferation depends on multiple factors. Culture media ingredients, such as carbohydrate sources, plant growth regulators, elicitors, etc., are also crucial for efficient PLB organogenesis and regeneration [205]. Growth retardants also stimulate PLB regeneration in orchids through the inhibition of GA biosynthesis [26]. Setting up the optimum temperature in the growth chamber is also necessary for PLB proliferation, and a higher or lower temperature compared to the optimum causes stress in PLB regeneration in orchids [209]. Light quality is another crucial factor for PLB organogenesis and regeneration for photosynthetic and phototropic responses, and many studies have suggested the efficiency of LEDs over traditional fluorescent light, suggesting the advantages of monochromatic light for PLB organogenesis and regeneration (Supplementary Table S4) [205]. However, different factors can work synergistically for better PLB organogenesis and regeneration compared with their independent applications. However, all these external factors are highly species-specific (Supplementary Table S5) [205]. We have also reported the manipulation of culture media and growth conditions for PLB regeneration in *Dendrobium* [30,209,210,211,212,213,214] and *Phalaenopsis* [26,215,216,217]. We found that culture media manipulation and light quality are highly species-specific in orchid PLB proliferation. 

Besides these techniques, seed culture, meristem culture, anther culture, embryo culture, ovule culture, cell suspension culture, and direct shoot organogenesis are also practiced for in vitro plantlet generation in ornamentals. 

## 4. Application of In Vitro Techniques in Ornamentals

Plant tissue culture is well known for producing disease-free plantlets by clonal propagation. In vitro culture offers a wide range of possibilities for manipulating plant materials to improve their quality. In vitro techniques are used for hybridization with the assistance of micropropagation, embryo rescue, and somatic hybridization.

### 4.1. Plant Improvement by the Application of In Vitro Embryo Rescue

**Figure 4:**
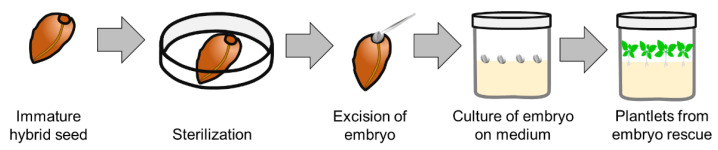
Process of embryo rescue from immature (or non-viable) seed after hybridization.

The technique of developing a viable plant from an embryo is known as embryo culture or embryo rescue (Figure 4). The embryo culture technique was introduced by Hannig, who cultured mature embryos of a few Brassicaceae plants on sugar-supplemented salt medium [218]. In 1924, Dietrich disclosed that both mature and immature embryos could be rescued [219]. The first interspecific hybridization by embryo rescue from nonviable seeds was reported in the perennial flax (*Linum perenne* L. × *Linum austriacum* L.) in 1925 [220]. Since the first report, embryo rescue has been used for interspecific hybridization in many crops, flowering, ornamentals, medicinals, and woody plants [221,222]. 

It allows for the culture of the ovary, ovule, and embryo [223,224,225]. The success of embryo rescue depends on various factors, such as size and age of the embryo, intactness of embryo, excision procedure, sterilization, culture medium, supplementation in culture medium, light, temperature, etc. [221,222]. It has been used in crop improvement by intraspecific/interspecific/intergeneric hybrid development, haploid/double haploid production, overcoming embryo abortion, overcoming seed dormancy, overcoming self- and cross-incompatibility, shortening the breeding cycle, propagating rare plants, etc. [226,227,228]. For example, breeding cycles were shortened by embryo rescue in rose [229], and lily [230]. Interspecific hybrids were developed in chrysanthemums by embryo rescue technique for cold-tolerant [224,225,231], heat-tolerant [232], drought-tolerant [233,234], salt-tolerant [235], aphid resistance [236], and heterotic [224,232,237] characteristics. A new flower shape and cold-tolerant intraspecific (*Campanula carpatica* ‘White’) and interspecific (*C. medium* and *C. formanekiana*) hybrid, respectively, were developed in bellflowers [238]. Interspecific hybrids, haploids, or double haploids were developed in rose [239,240,241], tulip [242], lisianthus [243], lily [244], day lily [245], calla lily [246], alstroemeria or peruvian lily [247,248,249,250], *Primula* [251,252], night-blooming cactus [253,254,255], gentian [256,257,258], *Camellia* [259], begonia [260], Christmas bells or golden lily of the valley [261], carnation [262,263], *Gypsophila* [264], *Rhododendron* [265], cyclamen [266], and ornamental alliums [267,268]. Embry rescue has been widely studied for crop improvement, while its current research has been reduced by the rapid evolution of advanced molecular breeding. 

In addition, embryo rescue is generally used to overcome post-fertilization barriers in plants, while many ornamentals have pre-fertilization barriers [269,270] that can be overcome by in vitro pollination. In in vitro pollination, plant reproductive cells (stigma and anther) are isolated and fused under controlled conditions to develop a zygotic embryo. The in vitro technique has been applied for in vitro flowering and pollination in different ornamentals [227,271].

### 4.2. Plant Improvement by Somatic Hybridization and In Vitro Pollination

**Figure 5:**
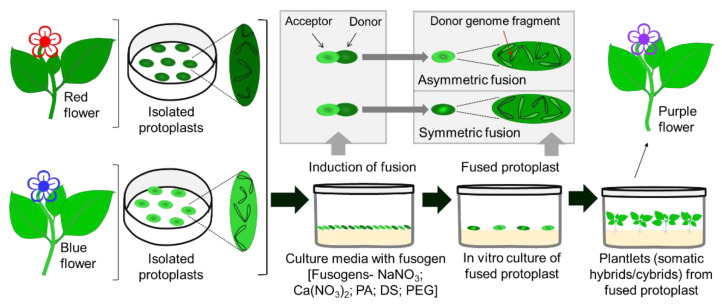
Illustration of somatic hybrid or cybrid development through protoplast fusion. Here, NaNO_3_; sodium nitrate, Ca(NO_3_)_2_; calcium nitrate, PA; polyvinyl alcohol, DS; dextran sulfate, polyethylene glycol (PEG).

Somatic hybridization has been proven to be a great source to produce genetic variability which is known as somaclonal variation. Many somaclones have been considered superior hybrids. Two methods are usually followed to produce the somatic hybrid, one is cytoplast-protoplast fusion and the other is the donor-recipient method. In cytoplast–protoplast fusion, protoplasts are allowed to fuse for combining somatic cells either fully or partially from different cultivars or species or genera (Figure 5). 

The combination of the nuclear genome of one parent with the mitochondrial and/or chloroplast genome of the other parent proceeds in somatic hybridization. An alternative and improved somatic incompatibility is the donor–recipient fusion method, where specific genes or chromosomes can be transferred [272,273]. Chemicals used for protoplast fusions are known as fusogens, and sodium nitrate (NaNO_3_), calcium nitrate (Ca(NO_3_)_2_), dextran sulfate, polyvinyl alcohol, and polyethylene glycol are common fusogens [274]. Somatic hybridization by protoplast fusion can develop either symmetric or asymmetric hybrids, which are known as somatic hybrids or cybrids (Figure 5). 

The first asymmetric hybrid was found in somatic hybridization through fusion between *Nicotiana tabacum* (tobacco) and *Petroselium hortense* (parsley) [275,276]. Many wild plant species have some significant traits, especially disease and pathogen resistance, and these traits can be transferred into cultivated crop species. Somatic hybridization allows the transfer of desirable traits to increase yield, resistance, tolerance, etc. [277,278]. It allows breeders to create novel hybrids by the asexual process, bypassing conventional breeding (Figure 5).

Somatic hybridization has been applied for the genetic improvements of various flowering and ornamentals, such as rose [72], *Dendrobium* [279], chrysanthemum [95], dianthus [280], gentin [281,282], iris [283], lily [284], petunia [285], between petunia and *Calibrachoa* [286], hydrangea [287], cyclamen [288], coneflower [289], and *Saintpaulia* [290].

Somaclonal variants or somatic hybrids can be confirmed by morphological, biochemical, protein marker, cytogenetic, and molecular analyses. Restriction fragment length polymorphism (RFLP), simple sequence repeat (SSR), amplified fragment length polymorphism (AFLP), methylation-sensitive amplification polymorphism (MSAP), transposon-based marker systems, and Next-Generation Sequencing (NGS) have been applied for the validation of somatic hybrids at the molecular level in several ornamentals [278]. Somaclonal variation is highly dependent on the PGRs [291]. The main constraints of somatic hybridization are the difficulties in isolating protoplasts (described in Section 3.2), generating unexpected and useless variations, newly generated variants that are not novel, etc. [278]. 

### 4.3. Production of Synthetic Seeds

**Figure 6:**
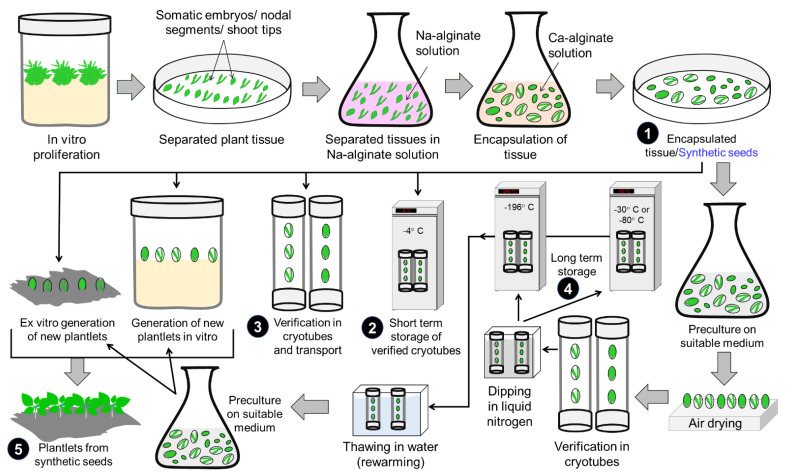
Production and application of synthetic seeds. The numbers in the figure represent the ending point of each step, such as the production of synthetic seeds (1), short-term storage of synthetic seeds (2), synthetic seeds for transportation (3), long-term storage of synthetic seeds (4), and plantlet generation from synthetic seeds (5).

Any encapsulated plant tissue, somatic embryos, or any other micropropagules is known as a synthetic seed or artificial seed (Figure 6). Synthetic seeds have several advantages over natural seeds, such as season-independent seed production, genetic uniformity, maintain hybrid vigor, long-term storage capacity, rapid multiplication, free from vegetative and seed-borne pathogens, propagation of high volume with low cost, assure quality plant materials, and shorten the life cycles [292,293]. Somatic embryos, nodal segments, and shoot tips are mostly used as explants for the development of synthetic seeds in ornamentals, while callus is rarely used, and PLBs are mainly used in orchids to produce synthetic seeds. Synthetic seeds have been generated in *Caladium bicolor* (caladium), *Eustoma grandiflorum* (lishianthus), *Pinus patula* (pine), *Genista monosperma* (bridal broom), *Hyoscyamus muticus* (Egyptian henbane), and *Clitoria ternatea* (bluepea or bluebellvine) from the somatic embryo; *Gypsophila paniculata* (gypsophila), *Saintpaulia ionantha* (saintpaulia), *Urginea altissima* (tall white squill), and *Taraxacum pieninicum* (Mniszek pieninski) from shoot tip; *Rosa* × *damascena* f. *trigintipetala* (Damask rose), *Syringa vulgaris* (lilac), *Nerium oleander* (oleander), *Centella asiatica* (Asiatic pennywort), *Eclipta alba* (false daisy), *Erythrina variegata* (tiger’s claw), *Photinia fraseri* (red tip photinia), *Ruta graveolens* (rue), *Salix tetrasperma* (Indian willow) from axillary buds/nodes, *Anthurium andreanum* (anthurium) from callus, *Lilium longiflorum* (easter lily) from bulb, and different species of orchids from PLBs (*Cymbidium giganteum*, *Vanda coerulea*, *Geodorum densiflorum*, *Coelogyne breviscapa*, *Cremastra appendiculata*, *Flickingeria nodosa, Spathoglottis plicata*, etc.) [292,293]. 

In vitro synthetic seeds in ornamentals allow season-independent seed production, to preserve for long term, and to supply in time to the growers. Some factors are crucial for the synthesis of artificial seeds in ornamentals; these are concentrations of sucrose, sodium alginate (Na-alginate), and calcium chloride (CaCl_2_). A range of 2–3% sucrose, 2–3% Na-alginate, and 50–100 mM CaCl_2_ was found to be effective concentrations for synthetic seed development in ornamentals [292,293].

Synthetic seeds have some limitations over the advantages: low efficient root systems, development of non-synchronous seeds from the somatic embryo (the most effective plant material for synthetic seed development), deviation from the normal structure, loss of embryogenic potential with time, etc. Synthetic seed technology can be used more effectively in the commercial ornamental plant propagation sector after resolving these limitations.

### 4.4. In Vitro Ploidy Manipulation

**Figure 7:**
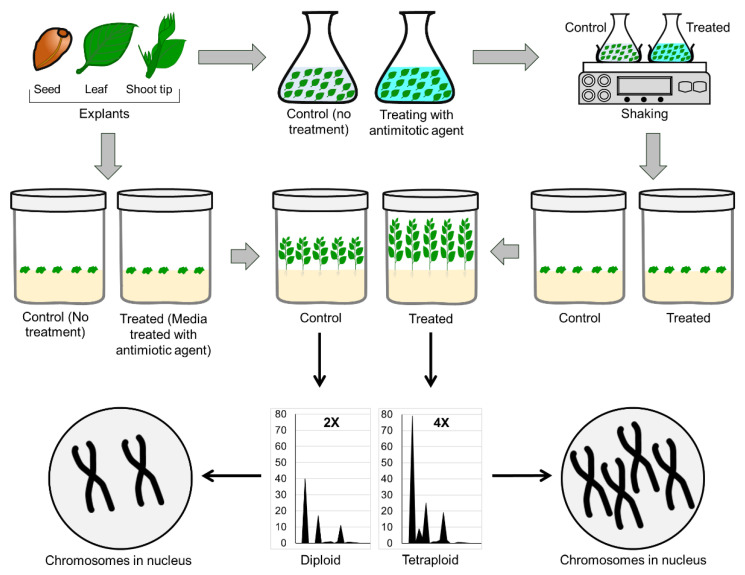
In vitro chromosome doubling (ploidy manipulation) for genetic diversification.

In vitro ploidy manipulation is a way of developing genetic diversification by increasing or decreasing chromosome numbers (Figure 7). The induction of polyploidy is used for crop improvement in ornamentals and can expand breeding opportunities to expand traits in ornamentals, environmental tolerances, and restore fertility in wide hybrids [294]. Two antimitotic agents, colchicine or oryzalin, are mostly used for chromosome doubling [295]. Two ginger lily lines: *Hedychium gardnerianum* Shepard ex Ker Gawl. and *H. coronarium* J. Koenig were used for chromosome doubling using colchicine or oryzalin and successfully developed the tetraploid ginger lily [296]. Forty—eight tetraploids were developed in ornamental aroid plants using colchicine (*Caladium* × *hortulanum* Birdsey) that showed variation in leaf shape, color, and thickness compared to the wild type [297]. Tetraploid anise hyssop (*Agastache foeniculum* L.) was induced by the application of colchicine, which showed a wide range of variation compared to diploid plants in their morphophysiological characteristics [298]. Polyploid has also been inducted in *Dendrobium*, *Phalaenopsis*, *Epidendrum*, and *Odontioda* orchids by the application of oryzalin [299]. Diverse phenotypic variations were observed in the in vitro-generated polyploids in rose, lilies, phlox, petunia, bellflowers, rhododendron, etc. [295]. Besides the antimitotic agents, ploidy manipulation also depends on the species, types of explants, antimitotic agent exposure method, duration of antimitotic agent exposure, etc. 

Diverse phenotypic variations were observed in the in vitro-generated polyploids in roses, lilies, phlox, petunia, bellflowers, rhododendron, etc. [295]. Besides the antimitotic agents, ploidy manipulation also depends on the species, types of explants, antimitotic agent exposure method, duration of antimitotic agent exposure, etc. The chromosome doubling technique produces only additional copies of chromosomes and genes, but it does not generate new genetic materials. However, it may cause morphological changes, anatomical changes, loss of duplicated genes, changes in gene expression, changes in epigenome status, and changes in epigenomic alteration-mediated gene expression, which ultimately lead to superior phenotypes in polyploids compared with diploids. These changes may also help generate resistance and tolerance capacity to biotic and abiotic stress.

## 5. Future Perspective

In vitro plant propagation and multiplication offer significant potential for the advancement of both basic and applied biological sciences. Rapid multiplication and propagation by callus culture, protoplast culture, somatic embryogenesis, PLB organogenesis, and direct plantlet regeneration allowed for the cheaper and disease-free seedling of a diverse ornamental plant species. Millions of in vitro plantlets of different ornamentals are generated worldwide for commercial purposes. However, it is important to put more effort into reducing the cost of production. In contrast to propagation, it also facilitates plant improvement following diverse techniques, such as embryo rescue, somatic hybridization, in vitro pollination, ploidy manipulation, the development of synthetic seeds, etc., and large numbers of hybrids in various ornamentals have already been developed. In addition, the in vitro technique is largely used for phytochemicals and secondary metabolite production. However, more effort is needed to reduce species-specific and other factor-specific responses for the efficient regeneration of ornamentals.

In recent years, researchers have started to study at the molecular level, including genetic transformation, using in vitro technology in ornamentals [300]. About 40 genera have been reported on creating transgenic ornamental species using *Agrobacterium tumefaciens*-mediated transformation [301]; however, only a few ornamentals, such as *Phalaenopsis* and petunia, have suitable and efficient transformation techniques. Some studies have revealed that many genes and transcriptions are involved in the in vitro organogenic callus, shoot, root, somatic embryos, and PLBs, and the transcriptions of those genes are also regulated by the exogenous application of different growth regulators [302]. 

**Figure 8:**
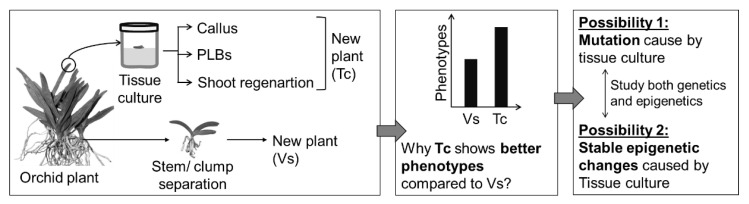
Prospects for advanced molecular research in plant tissue culture using orchid plants as an example. Here, Tc; Tissue culture regenerated plants, Vs; traditional vegetatively propagated plants.

It is believed that plant tissue culture generates genetically identical genotypes or somaclonal variants. Recent studies in *Arabidopsis* and crop plants, such as rice, wheat, corn, barley, and rye, have suggested that tissue culture can alter the genetic nature by point mutations [303]. In contrast to genetic factors, different epigenetic regulators, such as DNA methylation and histone modifications, are also involved in regulating the success of in vitro plant propagation [302,303]. Most of the genetic and epigenetic studies were conducted in the model plant *Arabidopsis* or crop plants, and this suggests the scope of future study in genetic and epigenetic aspects (Figure 8). 

Tissue culture alters genome-wide DNA methylation in the CG, CHG, and CHH contexts (H represents the A, C, or T), and these alterations change the gene expression that might be regulating factors for in vitro plant growth and development. DNA methylation was studied in the callus and somatic embryos of *Arabidopsis* and crop plants, and callus and somatic embryos are vulnerable to the alteration of DNA methylation, leading to changes in gene expression [303,304]. Involvement of di-methylated lysine 4 of histone H3 (H3K4me2) was associated with successful shoot regeneration from callus in *Arabidopsis* [305], while H3K4me3 and H3K27me3 histone marks are involved with the callus tissues in rice [306]. These reports suggest the importance of epigenetic regulation of in vitro regenerated plants. Besides DNA methylation and histone modification, different miRNAs and sRNAs may also be involved in the success of in vitro plant propagation. The expression of transposable elements (TEs) can also be epigenetically regulated in vitro environments; for example, TEs can be activated by the plant tissue culture [307]. However, there has been no significant advancement in the molecular mechanisms controlling in vitro regeneration in ornamentals. 

Studies on Arabidopsis and crop plants provide fundamental knowledge for disclosing the molecular mechanisms in ornamental plant species. Therefore, it is high time for advanced study of the genetic and epigenetic mechanisms that would provide a breakthrough in the commercialization of in vitro propagation of ornamental plant species. 

## Data Availability

Not applicable.

## Supplementary Materials

The following supporting information can be downloaded at: https://www.mdpi.com/article/10.3390/plants11233208/s1, Table S1: Effective plant growth regulators and other factors for the in vitro culture in ornamentals [38,110,111,113,114,123,173,192,308,309,310,311,312,313,314,315,316,317,318,319,320,321,322,323,324,325,326,327,328,329,330,331,332,333,334,335,336,337,338,339,340,341,342,343,344,345,346,347,348,349,350,351,352,353,354,355,356,357,358,359,360,361,362,363,364,365,366,367,368,369,370,371,372,373,374,375,376,377,378,379,380,381,382,383,384,385,386,387,388,389,390,391,392,393,394,395,396,397,398,399,400,401,402,403,404,405,406,407,408,409,410,411,412,413,414,415,416,417,418,419,420,421,422,423,424,425,426,427,428,429,430,431,432,433,434,435,436,437,438,439,440,441,442,443,444,445,446,447,448,449,450,451,452,453,454,455,456,457,458,459,460,461,462,463,464,465,466,467,468,469,470,471,472,473,474,475,476,477,478,479,480,481,482,483,484,485,486,487,488,489,490,491,492,493,494,495,496,497,498,499,500,501,502,503,504,505,506,507]; Table S2: Effective elicitors for the in vitro culture in ornamentals [26,51,215,216,217,340,404,458,470,482,508,509,510,511,512,513,514,515,516,517]; Table S3: Effective additives for the in vitro culture in ornamentals [342,347,350,363,365,504,518,519,520,521,522,523]; Table S4: Effective light emitting diodes (LEDs) for the in vitro culture in ornamentals [26,31,55,58,61,210,440,467,524,525,526,527,528,529,530,531,532,533,534,535,536,537,538,539,540,541,542,543,544,545,546,547,548,549,550,551,552,553,554]; Table S5: Studies in combination of light emitting diodes (LEDs) and plant growth regulators in vitro culture in ornamentals [26,31,55,58,61,62,210,317,440,467,524,525,526,527,528,529,530,531,532,533,534,535,536,537,538,539,540,541,542,543,544,545,546,547,548,549,550,551,552,553,554,555,556,557,558,559].
