# Supplementary material for: Tissue Culture in Ornamentals: Cultivation Factors, Propagation Techniques, and Its Application
Source: Plants (Basel). 2022 Nov 23;11(23):3208. doi: 10.3390/plants11233208 (PMC9736077; doi:10.3390/plants11233208)
Supplement: Supplementary file 1 [file plants-11-03208-s001.zip › plants-2010021-supplementary-final.pdf]

Tissue culture in ornamentals: Factors and techniques in propagation, and its application

Hasan Mehbub [1], Ayasha Akter [2], Mst. Arjina Akter [3,4], Md Shamim Hasan Mandal [5], Md. Ashraful Hoque [3], Monika Teluja [6], Hasan Mehraj [4,*]

[1] The United Graduate School of Agricultural Science, Ehime University, Matsuyama 790-8556, Japan
[2] Department of Horticulture, Bangladesh Agricultural University, Mymensingh, Bangladesh
[3] Department of Plant Pathology, Bangladesh Agricultural University, Mymensingh, Bangladesh
[4] Graduate School of Agricultural Science, Kobe University, Japan
[5] Graduate School of Advanced Science and Engineering, Hiroshima University, Japan
[6] Department of Plant Cytology and Embryology, Institute of Botany, Faculty of Biology, Jagiellonian University, Gronostajowa 9, 30-387 Krakow, Poland
* Correspondence: hmehraj02@yahoo.com or hmehraj02@port.kobe-u.ac.jp

Abbreviations		
**Medium**		
	MS	Murashige and Skoog
	1/2 MS	Half strength MS medium
	LM	Liquid media
	MS-LM	MS liquid medium
	mMS[1]	MS medium supplemented with 412.5 mg/L NH_4NO_3 and 950 mg/L KNO_3
	mMS[2]	MS medium supplemented with 100 mg/L $MgSO_4.7H_2O$, 100 mg/L NH_4NO_3, 30 mg/L Fe-EDTA, 1 mg/L thyamine
	mMS[3]	MS medium with the elimination of Cl^- and reduced NH_4^+ ions
	mMS[4]	MS medium with extra addition of 330 mg/L $CaCl_2{\cdot}6H_2O$, 13.9 mg/L $FeSO_4$, 20.65 mg/L $Na_2EDTA{\cdot}2H_2O$
	NN	Nitsch and Nitsch
	NDM	New Dogashima medium
	VW	Vacin and Went
	mVW	Ferric tartrate replaced by Na_2EDTA 37.3 mg/L and $FeSO_4{\cdot}7H_2O$ 27.8 mg/L
	uVW	Modified VW medium, modification is not specified
	B5	Gamborg medium
	WH	White medium
	WPM	Woody Plant Medium
	SH	Schenk & Hildebrandt medium
	TC	Teixeira Cymbidium medium
	TC1	Teixeira Cymbidium medium 1
	mHm	Modified Hyponex medium (6.5 N4.5 P–19K + 20N–20P–20K)
	SM	Simplified medium with 5 mL/L of Biofert Plus® NPK (08-09-09) fertilizer and 60 g/L 'Nanica' banana pulp
	MM	Basal medium of Mitra et al. (1976)
	KC	Knudson's C media
	mKC	Modified by $ZnSO_4.7H_2O$ (from 0.331 to 6.62 mg/L), H_3BO_3 (from 0.056 to 1.4 mg/L) and $MnSO_4.H_2O$ (from 7.5 to 15 mg/L)
	mNS	Modified nutrient solution of KC, replacement of the iron source by iron-EDTA of MS medium
	O753	Orchid Multiplication Medium, PHYTAMAX™
	P0931	Phytamax basal medium
	NP	Ichihashi New Phalaenopsis Medium
	CIM	Callus induction medium [half strength MS macro- and micronutrients, 250 mg/L peptone, 100 mg/L myo-inositol, 2 mg/L glycine, 0.5 mg/L nicotinic acid, 0.1 mg/L thiamine-HCl, 0.5 mg/L pyridoxin-HCl, 1 mg/L MES]
	CM	Chen medium
	DKW	Driver and Kuniyuki walnut (Driver and Kuniyuki 1984)
**Carbon sources**		
	suc.	Sucrose
	mal.	Maltose
	glu.	Glucose
	tre.	Trehalose
**Solidifying agents**		
	ag	Agar
	gg	Gellan gum
	pg	Phytagel
	gr	Gelrite
	bag	Bacto agar
	cg	Clarigel
	cn	Carrageenan
	pa	Phyto agar
**Light**		

	WF	White fluorescent
	PAR LED	Photosynthetically Active Radiation (blue; 430 nm 5%, 460 nm 10%; red, 610 nm 10%, 630 nm 35%, 660 nm 35%, 730 nm 5%)
**Plant growth regulators (PGRs)**		
**Auxin**	IAA	Indole-3-acetic-acid
	IBA	Indole-3-butyric acid
	NAA	1-Napthaleneacetic acid
	2,4-D	2, 4-Diclorophenoxiacetic acid
	Pic	Picloram (4-amino-3,5,6-trichloropicolinic acid), a synthetic auxin
**Cytokinins**	BAP	6-benzylaminopurine
	BA	6-benzyladenine
	KIN	Kinetin
	ZT	Zeatin
	TDZ	Thidiazuron (N-phenyl-N'-1,2,3-thiadiazol-5-yl urea)
	mT	meta-Topolin
	ZR	Zeatin riboside
**Gibberellins**	GA_3	gibberellic acid
**Elicitor**	Ch	Chitosan
	O-Ch	Oligomeric-Chitosan mixture
	P-Ch	Polymeric-Chitosan
	5-ALA	5-aminolevulinic acid
	NAG	*N*-acetylglucosamine
	SA	Salicylic acid
	L-phe	L-Phenylalanine
	HA9	Hyaluronic Acid with molecular weight 1.08x106 Da
	HA12	Hyaluronic Acid with molecular weight 1.2x106 Da
	JA	Jasmonic acid
	MeJA	Methyl jasmonate
	PG	P hloroglucinol
**Carbocyclic sugar**		
	MI	*myo*-inositol
	MeI	*meso*-inositol
**Plant hormones**		
	24-EpiB	Epin-Extra® (24-epibrassinolide)
	RE	Ribav-Extra®
	TRIA	Triacontanol
**Lanthanoids**		
	Nd	Neodymium nitrate

	CCC	Chlorocholine chloride (CCC: (2-chloroethyl) trimethyl-ammonium chloride)				
	MES	4-morpholineethanesulfonic acid				
	NAg	Nano-silve				
	2iP	6-(γ,γ-dimethylallylamino)purine				
	ac	Activatedd charcoal				
	HYPONeX	liquid, N:P:K=6:3:3				
**Misc.**						
	Thi	Thiamine	Bio	Biotin	AS	Adenine sulphate
	L-gl	L–glutamine	Nia	Niacin	CA	Citric acid
	Pt	Peptone	PHCl	Pyridoxine HCl	L-arg	L-arginine
	p–ABC	*p*–aminobenzoic acid	gly	Glycine	6P	6-(4-hydroxy-3-methylbut-2- enylamino) purine
	T–HCl	Thiamine HCl	Chl	Casein hydrolysate	CPPU	N-(2-chloro4-pyridl)-N′-phenylurea
	cw	Coconut water	pyr	Pyridoxine	Tt	Tryptone
	BH	Banana homogenate	NA	Nicotinic acid	Spe	Spermin, a Polyamines
	PH	Potato homogenate	pac	Paclobutrazol	Orn	Ornithyne, a Polyamines
	TH	tomato homogenate	glu	Glutamine	wks	Weeks
	Pu	Putrescine, a Polyamines	AA	Ascorbic acid		

Table S1. Effective plant growth regulators and other factors for the in vitro culture in ornamentals.

Plant species	Media	Carbon source	Solidifying agent	Media supplements	Effective concentration and combinations	Temperature	Light / dark cycle	Culture period	Target of regeneration	Ref
**Cymbidium orchid**										
Cymbidium Twilight Moon "Day Light"	TC	2% suc.	0.8% bag	–	1-2 mg/L 2,4-D or 1 mg/L TDZ	25 °C	16 h/ 8 h	–	Embryogenic callus	da Silva 2014a
Cymbidium aloifolium	MS	3% suc.	0.8% ag	100 mg/L MI	5 mg/L NAA	25±1 °C	–	8 wks	Rhizomes	Nayak et al. 1998
C. aloifolium	MS	3% suc.	0.8% ag	100 mg/L MI	1 mg/L BA + 0.1 mg/L NAA	25±1 °C	–	8 wks	Shoot/rhizome	Nayak et al. 2002
C. aloifolium Sw.	MS	3% suc.	0.8% ag	100 mg/L MI	14 μM ZR (PLBs); 9.8 μM IBA (root)	25±1 °C	–	10 wks	PLBs and root induction	
Cymbidium "Memoria Amelia Earhart"	1/2 MS	–	0.7% ag	0.15% ac	10^6% 24-EpiB; 11.6 μM KIN + 2.9 μM IAA	22±2 °C	16 h/ 8 h	6 wks	PLBs	Lukatkin et al. 2019
	MS	–	0.7% ag	0.15% ac	10^4%-10^5% 24-EpiB; 0.5 μM KIN + 2.9 μM IAA	22±2 °C	16 h/ 8 h	6 wks	Shoot organogenesis	
	MS	–	0.7% ag	0.15% ac	10^4% RE; 4.4 μM or 13.3 μM BA; 109 μM TDZ + 2.9 μM IAA	22±2 °C	16 h/ 8 h	6 wks	Shoot organogenesis	
Cymbidium Waltz "Idol"	mMS[1]	2% suc.	0.2% pg	–	0.1 mg/L HA9	25±2 °C	16 h/ 8 h	40 days	PLBs proliferation	Kaewjampa et al. 2012
C. insigne	mMS[1]	2% suc.	0.2% pg	–	0.1 mg/L CS	–	16 h/ 8 h	8 wks	PLBs proliferation	Nahar et al. 2013
C. faberi Rolfe	1/2 MS	3% suc.	0.68% ag	2 g/L ac + 0.5 g/L Chl	0.5, 1, 2 mg/L NAA	25±2 °C	12 h/ 12 h	30 days	PLB organogenesis and proliferation	Tao et al. 2011
	1/2 MS	3% suc.	0.68% ag	3 g/L ac + 0.5 g/L Chl	1 mg/L TDZ + 0.5 mg/L NAA (shoot); 0.5 mg/L IBA (root)	25±2 °C	12 h/ 12 h	30~60 days	Shoot and root organogenesis	
C. dayanum	mMS[1]	2% suc.	0.2% pg	–	0.1 mg/L Ch; 0.1 mg/L HA9	25 °C	16 h/ 8 h	40 days	PLBs proliferation	Nahar et al. 2011
C. iridioides D. Don	MS	–	0.8% ag	–	0.5 mg/L BAP (shoot); 1 mg/L IBA (root)	25±2 °C	16 h/ 8 h	8 wks	Plantlet regeneration	Pant and Swar 2011
C. f inlaysonianum	MS	–	0.6% ag	–	1.5 mg/L BAP + 0.75 mg/L NAA	25±2 °C	16 h/ 8 h	45 days	Shoot regeneration	Islam et al. 2015
**Dendrobium orchid**										
Dendrobium hybrid	MS	2% suc.	0.8% ag	1 g/L ac	1 mg/L NAA + 1 mg/L BAP	26±1 °C	16 h/ 8 h	60 days	PLBs and plantlet regeneration	Khatun H et al. 2010
Dendrobium hybrid	MS	2% suc.	0.8% ag	–	1.0 mg/L IAA + 1.0 mg/L IBA	26±1 °C	16 h/ 8 h	–	Rooting	Khatun M et al. 2010
Dendrobium hybrid (Sonia 17 and 28)	1/2 MS	2% suc.	0.7% ag	–	44.4 μM BA + 6.97 μM KIN	25±2 °C	16 h/ 8 h	60 days	PLBs and shoot organogenesis	Martin and Madassery 2006
	1/2 MS	2% suc.	0.7% ag	–	0.44 μM BA (shoot); 2 g/L ac (root)	25±2 °C	16 h/ 8 h	60 days	PLBs to shoots induction, root induction	
Dendrobium sp.	MS	–	–	–	10 mg/L 2,4-D (PLBs); 0.5 mg/L BAP + 0.5 mg/L NAA (shoot)	25±1 °C	–	60 days	PLBs and shoot regeneration	Goswami et al. 2015
Dendrobium sp.	MS	3% suc.	0.8% ag	–	2.5 mg/L NAA; 0.5 or 2.5 mg/L BAP	25±2 °C	16 h/ 8 h	30 & 60 days	PLBs proliferation	Hossen et al. 2021
	MS	3% suc.	0.8% ag	–	0.5 mg/L KIN + 2.5 mg/L BAP + 0.5 mg/L NAA	25±2 °C	16 h/ 8 h	45 days	Shoot regeneration	
	MS	3% suc.	0.8% ag	–	0.5 mg/L KIN + 2.5 mg/L BAP + 5 mg/L NAA	25±2 °C	16 h/ 8 h	45 days	Root induction	
D. densiflorum Lindl. ex Wall.	MS	3% suc.	0.7% ag	100 mg/L MI	5 mg/L BAP + 0.1 mg/L NAA	25±2 °C	16 h/ 8 h	2 months	PLBs proliferation	Luo et al .2008
	MS	3% suc.	0.7% ag	100 mg/L MI	2 mg/L BAP (shoot); 5 mg/L IBA + 5 g/L ac or 2 mg/L Nd (root)	25±2 °C	16 h/ 8 h	–	Shoot and root from PLB	
D. densiflorum Lindl.	MS	–	0.8% ag	–	2 mg/L BAP + 0.5 mg/L NAA (shoot); 1.5 mg/L IBA (root)	25±2 °C	16 h/ 8 h	12 wks	Shoot and root proliferation	Pradhan et al. 2013

Dendrobium cv. Sonia	1/2 MS	3% suc.	0.7% ag	–	1 mg/L BA	25±2 °C	16 h/ 8 h	–	PLBs multiplication	Sheela et al. 2006
D. nobile	MS	3% suc.	0.8% ag	–	1 mg/L mT + 0.5 mg/L NAA + 0.8 mg/L Pu	25±2 °C	12 h/ 12 h	8 wks	shoot proliferation	Bhattacharyya et al. 2016
D. nobile var. Emma white	O753	–	–	–	2 mg/L BAP (shoot); 2 mg/L IBA (root)	–	–	6 wks	Shoot and root proliferation	Asghar et al. 2011
D. nobile Lindl.	MM	3% suc.	0.7% ag	0.5 g/L MeI + 1 g/L Chl + 0.5 g/L L–gl + 250 mg/L Pt + 0.2 g/L *p*- ABC + 0.1 g/L Bio	4 μg/L TRIA (PLB); 2 μg/L TRIA (shoot and root)	25±3 °C	–	6~12 wks (PLB), 5~10 wks (shoot and root)	PLBs and shoot organogenesis	Malabadi et al. 2005
D. nobile Lindl.	MS	3% suc.	0.8% ag	100 mg/L MI	11 μM BA (PLBs); 9.8 μM IBA (root)	25±1 °C	–	10 wks	PLBs, root induction	Nayak et al. 2002
D. fimbriatum Lindl. var. *oculatum* Hk. f.	mNS	–	0.9% ag	10% cw + 0.5 mg/L Nia + 0.5 mg/L + PHCl + 0.1 mg/L T-HCl	0.5 mg/L NAA + 1 mg/L BAP	25±2 °C	10 h/ 14 h	8 wks (PLB)	Callus and shoot regeneration	Roy and Banerjee 2003
D. fimbriatum Lindl. var. *oculatum* Hk. f.	MM	3% suc.	0.9% ag	–	2.4 mg/L (PLB formation); 4.8 mg/L KIN (shoot); 2.4 mg/L BAP (root)	25±2 °C	16 h/ 8 h	–	PLBs, shoor-root generation	Tikendra et al. 2021
D. palpebrae Lindl.	MS	–	0.8% ag	–	1 mg/L NAA + 2 mg/L BAP (in number); 1 mg/L Pic + 2 mg/L BAP (in length)	25±2 °C	14 h/ 10 h	–	Multiple shoot bud initiations	Bhowmik et al. 2020
D. candidum Wall. Ex Lindl.	1/2 MS, LM	3% suc.	–	1 ml/L HYPONeX	2 mg/L BA + 0.1 mg/L NAA	25±1 °C	16 h/ 8 h	75 days	Shoot regeneration	Shiau et al. 2005
D. candidum Wall. Ex Lindl.	1/2 MS	2% suc.	0.6% ag	–	8.8 μM BA + 9.2 μM 2,4-D (callus); 1.08-2.7 μM NAA (Shoot)	25±2 °C	12 h/ 12 h	60 days	Callus and shoot development	Zhao et al. 2008
D. heterocarpum Wall. ex Lindl.	MS	3% suc.	0.8% ag	–	3 μM/L KIN + 12 μM/L NAA	25±2 °C	12 h/ 12 h	–	Plantlet regeneration	Longchar and Deb 2022
D. primulinum Lindl.	MS	3% suc.	0.8% ag	–	1.5 mg/L BAP + 0.5 mg/L NAA (shoot); 0.5 mg/L IAA (root)	25±2 °C	16 h/ 8 h	24 wks (shoot), 16 wks (root)	Shoot multiplication and rooting	Pant and Thapa 2012
D. chrysotoxum Lindl	MM	2% suc.	0.9% ag	–	3.5 mg/L BAP + 0.5 mg/L IBA (shoot); 0.5 mg/L BAP + 3.0 mg/L IBA (shoot)	25±2 °C	12 h/ 12 h	–	Plantlet regeneration	Tikendra et al. 2019
D. chrysanthum	MS	–	0.8% ag	–	5 μM TDZ + 5 μM BAP (shoot); 10 μM NAA	25±2 °C	12 h/ 12 h	30 days	Shooting and rooting	Hajong et al. 2013
D. thyrsiflorum	MS	–	-	–	2 mg/L of TDZ + 0.5 mg/L of NAA	25±1 °C	12 h/ 12 h	5 wks	PLBs and indirect shoot organogenesis	Bhattacharyya et al. 2015
	MS	–	0.3% cg	–	2 mg/L 2iP + 0.5 mg/L IBA	25±1 °C	12 h/ 12 h	–	direct shoot organogenesis	
	1/2 MS	–	-	–	2 mg/L IBA + 0.6 mg/L NAA	25±1 °C	12 h/ 12 h	–	Root induction	
D. wangliangii	1/2 MS	2% suc.	0.56% ag	–	1 mg/L BA + 0.1 mg/L NAA	22±2 °C	16 h/ 8 h	90 days	PLBs organogenesis	Zhao et al. 2013
	1/2 MS	2% suc.	0.56% ag	–	2 mg/L BA + 0.1 mg/L NAA	22±2 °C	16 h/ 8 h	70 days	Shoot organogenesis	
	1/2 MS	2% suc.	0.56% ag	–	2 mg/L of TDZ	22±2 °C	16 h/ 8 h	–	Inflorescences induction	
D. officinale	MS	3% suc.	0.55% cn	–	2 mg/L BA + 0.1 mg/L NAA	26±2 °C	12 h/ 12 h	60 days	Shoot multiplication	Chen et al. 2014
D. formosum	MS	–	–	–	1 mg/L 2,4-D (PLB); 2.5 mg/L BAP + 1 or 2 mg/L NAA (plantlets)	25±1 °C	16 h/ 8 h	60 days	PLBs proliferation and plantlet	Nasiruddin et al. 2003
D. bensoniae	MS	–	–	–	1 mg/L BA + 1.5 mg/L IBA	25±2 °C	14 h/ 10 h	60 days	Shoot regeneration	Riva et al. 2016

**Phalaenopsis orchid** (reviewed at Khatun et al. 2020)										
Phalaenopsis cv. "Ph908"	NDM	2% suc.	0.3% ag	0.1 g/L MI, 2 mg/L BA, 1 mg/L NAA, 2 mg/L GA3	0.25 mg/L TDZ	25±2 °C	0 h/ 24 h	60 days	PLB formation	Zanello and Cardoso 2019
Phalaenopsis cv. "RP3"	NDM	2% suc.	0.3% ag		0.125 mg/L TDZ + 1 mg/L NAA + 20 mg/L BA	25±2 °C	0 h/ 24 h	60 days	PLB formation	
P. amabilis cv. "Blume"	NP	–	–	–	3 mg/L TDZ + 1 mg/L NAA	25±1 °C	0 h/ 24 h (1st 14 days); 16 h/ 8 h	8 wks	Embryo formation	Mose et al. 2020
P. amabilis cv. "Surabaya"	CM	–	–	–	5 mg/L BAP + 2 mg/L NAA (PLBs); 2 mg/L NAA (root)	25±1 °C	16 h/ 8 h	160 days	PLBs formation and rooting	Balilashaki et al. 2015
P. amabilis var. *formosa*	1/2 MS	2% suc.	0.22% gr	100 mg/L MI + 0.5 mg/L Nia + 0.5 mg/L + PHCl + 0.1 mg/L T–HCl + 2 mg/L gly + 1000 mg/L Pt + 170 mg/L NaH_2PO_4	3 mg/L TDZ	26±1 °C	16 h/ 8 h	45 days	Embryo formation	Chen and Chang 2006
P. amabilis Shimadzu var. *formosa and Phalaenopsis* "Nebula"	1/2 MS	2% suc.	0.22% gr	100 mg/L MI + 0.5 mg/L Nia + 0.5 mg/L + PHCl + 0.1 mg/L T–HCl + 2 mg/L gly + 1000 mg/L Pt + 170 mg/L NaH_2PO_4	4 mg/L TDZ (embryo); 0.5 mg/L of BA (plantlet)	26±1 °C	0 h/ 24 h	60 days	Embryo formation and plantet generation	Gow et al. 2009; Gow et al. 2010
P. gigantea	NDM	–	–	–	1 mg/L NAA + 0.1 mg/L TDZ	25±2 °C	–	6 wks	Callus and PLBs formation	Niknejad et al. 2011
Phalaenopsis "Little Steve"	1/2 MS	2% suc.	0.22% gr	100 mg/L MI + 0.5 mg/L Nia + 0.5 mg/L + PHCl	4.54 μM TDZ	26±1 °C	0 h/ 24 h	30 days	Embryo formation	Kuo et al. 2005
				+ 0.1 mg/L T–HCl + 2 mg/L gly + 1000 mg/L Pt + 170 mg/L NaH_2PO_4						
P. amabilis BL. cv. "Golden Horizon"	1/2 MS	2% suc.	0.22% gr	10% cw + 2 g/L Pt + 1 g/L ac + 150 mg/L L–gl	2 mg/L BA + 0.5 mg/L NAA	24±1 °C	16 h/ 8 h	12 wks	PLBs	Sinha and Jahan 2011
P. cornu-cervi (Breda) Blume & Rchb. f.	1/2 MS	–	–	–	0.1 mg/L TDZ + 0.1 mg/L NAA (PLB)	25±1 °C	16 h/ 8 h	45 days	PLBs	Rittirat et al. 2012
	MS	–	–	–	15% cw + 0.2% ac (root)	25±1 °C	16 h/ 8 h	45 days	Root induction	
**Oncidium orchid**										
Oncidium sp. "Dancing Dolls"	MS	–	0.8% ag	–	2 mg/L BAP + 1.5 mg/L NAA	25±1 °C	12 h/ 12 h	–	Shoot and root organogenesis	Kalimuthu et al. 2007
O. sp. "Gower Ramsey"	1/2 MS	2% suc.	0.22% gr	100 mg/L MI + 2 mg/L gly + 1000 mg/L Pt + 170 mg/L NaH_2PO_4	0.1 μM TIBA or 0.5 μM TIBA	26±1 °C	16 h/ 8 h	8 wks	Embryo formation	Chen et al. 2004
O. tigrinum Llave & Lex.	MS	3% suc.	0.8% ag	–	1-2 mg/L BA + 0.1 mg/L NAA (shoot); 1 g/L ac (root)	25±1 °C	16 h/ 8 h	90 days	Shoot and root induction	Mata-Rosas et al. 2011
O. flexuosum Sims	MS	3% suc.	0.5% ag	–	1.5 μM TDZ	25±2 °C	0 h/ 24 h	90 days	PLBs regeneration	Mayer et al. 2010
**Vanda orchid**										
Vanda tessellata (Roxb.) Hook. ExG.Don	MS	3% suc.	ag	–	1.5 mg/L BAP + 0.5 mg/L NAA	25±2 °C	16 h/ 8 h	30 days	Seedlings development and shoot elongation	Bhattacharjee et al. 2014

	MS	3% suc.	ag	–	1 mg/L BAP + 1 mg/L NAA	25±2 °C	16 h/ 8 h	28-35 days	Shoot induction	
	1/2 MS	3% suc.	ag	–	1 mg/L IAA	25±2 °C	16 h/ 8 h	–	Root induction	
V. coerulea Griff ex.Lindl. (Blue Vanda)	P0931	3% suc.	0.7% ag	3 g/L ac	5.36 μM + 3.80 μM	24±2 °C	16 h/ 8 h	8 wks	PLBs proliferation	Roy et al. 2011
V. spathulata Spreng	MM	2% suc.	0.6% ag	–	2:1μM BA:IAA	28±2 °C	12 h/ 12 h	3 months	Shoot multiplication	Decruse et al. 2003
**Coelogyne orchid**										
Coelogyne cristata	1/2 MS	3% suc.	0.8% ag	–	0.44 mg/L TDZ	22~25 °C	–	60 days	Shoot induction from shoot axis	Naing et al. 2010
C. cristata	1/2 MS	3% suc.	0.8% ag	–	1 mg/L NAA + 2 mg/L BA	22~25 °C	–	8 wks	Shoot induction from pseudobulb	
C. ovalis	KC	3% suc.	0.8% ag	–	10 μM mT + 0.5 μM NAA; 15 μM BAP + 0.5 μM NAA	25±2 °C	16 h/ 8 h	45 days	Shoot and PLBs – induction and proliferation	Singh et al. 2020
C. flaccida	MS	–	0.8% ag	–	4.44 μM NAA + 5.37 μM BA	25±2 °C	12 h/ 12 h	–	Pseudobulb segments regeneration	Kaur and Bhutani 2013
C. flaccida	MS	–	–	–	2 mg/L NAA + 2 mg/L KIN	20±2	16 h/ 8 h	15 wks	Plantlets regeneration	Kalyan and Sil 2015
**Bulbophyllum orchid**										
Bulbophyllum auricomum Lindl.	MS	3% suc.	0.8% ag	15% cw	2 mg/L BAP + 1.0 mg/L NAA	25±2 °C	16 h/ 8 h	1 and 2 months	Callus induction and PLB proliferation	Aung et al. 2022
B. odoratissimum	MS	3% suc.	0.8% ag	–	4 mg/L BA + 0.5 mg/L IBA	25±2 °C	16 h/ 8 h	12 wks	Shoot multiplication	Prasad et al. 2021
**Paphiopedilum orchid**										
Paphiopedilum rothschildianum	1/2 MS	3% suc.	3% gr	–	4.0 μM KIN	25±2 °C	0 h/ 24 h	8 wks	PLB formation	Ng et al. 2011
P. rothschildianum	1/2 MS	58 mM suc.	0.22% gr	full-strength MS vitamins, 2 g/L Pt, 170 mg/L NaH_2PO_4	13.56 μM 2,4-D + 4.54 μM TDZ	25±2 °C	0 h/ 24 h	90 days	PLB formation	Masnoddin et al. 2016
**Other genus of orchid**										
Guarianthe skinneri "Bateman"	MS	3% suc.	0.25% pg	0.1 g/L MI + 0.05 g/L $NaHPO_4$	16.1 μM NAA; 17.1 μM IAA; 6.3×10^{-9} μM GA3; 0.0023 μM BA	25~27 °C	16 h/ 8 h	2 month	Shoot regeneration	Coello et al. 2010

	MS	3% suc.	0.25% pg	0.1 g/L MI + 0.05 g/L $NaHPO_4$	5.4 μM NAA; 17.1 μM IAA; 0.001 μM GA3; 4.6×10^{-9} μM BA (root)	25~28 °C	16 h/ 8 h	3 months	Root regeneration	
Orchis catasetum	MS	3% suc.	0.8% ag	-	0.5 mg/L BA + 0.5 mg/L NAA	24±2 °C	16 h/ 8 h	60 days	PLBs proliferation & plantlet regeneration	Baker et al. 2014
Eulophia dabia (D. Don) Hochr.	MS	2% suc.	0.9% ag	0.2% ac	1 mg/L BAP 0.5 mg/L NAA; 0.1 or 1 mg/L TDZ (for PLBs)	25±2 °C	12 h/ 12 h	–	Plantlet regeneration	Chauhan et al. 2015
Grammatophyllum speciosum	1/2 MS-LM	2% mal.	–	–	–	25±2 °C	16 h/ 8 h	2 months	PLBs and shoot formation	Sopalun et al. 2010
	1/2 MS-LM	2% suc.	–	–	15 mg/L Ch	25±2 °C	16 h/ 8 h	2 months	PLBs and shoot formation	

	1/2 MS	2% suc.	–	–	2.0 mg/l NAA + 1.0 mg/l BA	25±2 °C	16 h/ 8 h	2 months	PLBs and shoot formation	
Dimorphorchis lowi	KC	2% suc.	0.8% ag	–	2 mg/L KIN (shoot); 1 mg/L IAA + 0.5 mg/L IBA (root)		24 h/ 0 h	180 days	Shoot and root regeneration	Jainol and Gansau 2016
Serapias vomeracea	KC	2% suc.	0.35% pg	–	0.5 mg/L JA; 0.5 mg/L O-Ch	23±1 °C	16 h/ 8 h	90 days	PLB formation	Acemi et al. 2020
	KC	2% suc.	0.35% pg	–	15-20 mg/L P-Ch	23±1 °C	16 h/ 8 h	180 days	Shoot induction	
	KC	2% suc.	0.35% pg	–	10 mg/L O-Ch, 15 mg/L P-Ch	23±1 °C	16 h/ 8 h	180 days	Root induction	
	KC	2% suc.	0.35% pg	–	0.5 mg/L JA; 10 mg/L O-Ch	23±1 °C	16 h/ 8 h	180 days	Tuberization	
Rhynchostylis retusa	VW	2% suc.	–	–	0.1 mg/L NAA + 1 mg/L KIN or BAP	25±1 °C	14 h/ 10 h	–	Callus, PLBs, plantlets	Sunitibala et al. 2018
Hybrid (*Aranda* Wan Chark Kuan "Blue" × *Vanda coerulea* Grifft. ex. Lindl.)	MS	3% suc.	– gr	100 mg/L MI	1.5 mg/L TDZ (PLB); 1.0 mg/L BA + 0.5 m/L IBA (Plantlets)	25±1 °C	14 h/ 10 h	40 (PLBs), and 30 (pllantlets) days	PLB induction and plantlets generations	Gantait et al. 2012
Esmeralda clarkei Rchb. f.	MS	–	0.8% ag	–	1 mg/L BAP (shoot); 1 mg/L NAA (root)	25±2 °C	16 h/ 8 h	120 days	Shoot multiplication and rooting	Paudel and Pant 2012
Aenhenrya rotundifolia (Blatt.) C.S. Kumar and F.N. Rasm.	MM	2% suc.	0.7% ag	–	6.20 μM mT + 2.25 μM TDZ; 6.20 μM mT + 5.37 μM NAA	23±2 °C	16 h/ 8 h	4 wks	shoot proliferation	Sherif et al. 2020
Caladenia huegelii	1/2 MS	60 mM suc.	0.8% ag	500 μM MI + 500 μM MES + 1 μM T–HCl + 2.5 μM pyr + 4 μM NA	5 μM NAA + 2 μM BA	20±0.5 °C	–	–	PLB proliferation	Bustam et al. 2016
Cyrtopodium paludicolum	KC	58.43 mM suc.	0.4% ag	–	1.34 μM NAA + 2.27 μM TDZ	27±2 °C	16 h/ 8 h	–	PLBs proliferation from root tip	Picolotto et al. 2017
Ansellia africana Lindl	MS	3% suc.	0.8% ag	–	0 μM mT + 5 μM NAA +15 μM IBA + 30 μM PG	25±2 °C	12 h/ 12 h	10 wks	Shoots with roots organogenesis	Saleh-E-In et al. 2021
Tolumnia Snow Fairy	1/2 MS	3% suc.	0.8% ag	100 mg/L MI + 170 mg/L NaH_2PO_4	4 mg/L BA	25±2 °C	12 h/ 12 h	16 wks	PLBs organogenesis	Chookoh et al. 2019
Cyrtopodium paranaense	MS	3.5% suc.	1% ag	–	0.2 μM IAA + 9 μM TDZ	25-27 °C	0 h/ 24 h	3 wks	PLBs organogenesis	Guo et al. 2010
Brassavola nodosa "Remar" x "Mas Mejor" hybrid	1/2 MS	3% suc.	0.7% ag	2 mg/L gly + 0.1 mg/L NAA + 30% cw	2 mg/L BA + 3 mg/L adenine sulfate	26±4 °C	16 h/ 8 h	45 days	Shoot multiplication	Xu et al. 2022
	1/2 MS	3% suc.	0.7% ag	2 mg/L gly + 0.1 mg/L NAA + 30% cw	0.5 mg/L NAA or 1 mg/L IBA	26±4 °C	16 h/ 8 h	45 days	Root induction	
**Lily**										
Cyrtanthus contractus (Fire lily)	MS	3% suc.	0.8% ag	0.1 g/L MI	4.4 μM BA + 1.1 μM NAA	25±2 °C	16 h/ 8 h	8 wks	Shoot induction	Ncube et al. 2015
C. guthrieae (Berg lily)	MS	3% suc.	0.8% ag	0.1 g/L MI	4.4 μM BA + 1.1 μM NAA	25±2 °C	16 h/ 8 h	8 wks	Shoot induction	
C. obliquus (Knysna Lily)	MS	3% suc.	0.8% ag	0.1 g/L MI	6.7 μM BA + 2.7 μM NAA	25±2 °C	16 h/ 8 h	8 wks	Shoot induction	
C. contractus (Fire lily)	MS	3% suc.	0.8% ag	0.1 g/L MI	4.4 μM BA + 1.1 μM NAA + 10 μM TDZ	25±2 °C	16 h/ 8 h	8 wks	Shoot organogenesis	
C. guthrieae (Berg lily)	MS	3% suc.	0.8% ag	0.1 g/L MI	4.4 μM BA + 1.1 μM NAA	25±2 °C	16 h/ 8 h	8 wks	Shoot organogenesis	
C. obliquus (Knysna Lily)	MS	3% suc.	0.8% ag	0.1 g/L MI	6.7 μM BA + 2.7 μM NAA + 10 μM BA	25±2 °C	16 h/ 8 h	8 wks	Shoot organogenesis	
Hemerocallis sp (19 Day lily cultivars)	MS	–	0.4% pg	–	1 , 5 or 10 mg/L TDZ	–	–	69 days	Shoot organogenesis	Matand et al. 2020

**Oriental lily**										
Lilium orientalis cv. "Starfighter" (Oriental lily)	MS	3% suc.	0.7% ag	–	0.5 mg/L TDZ + 10 mg/L 2,4-D	23±2 °C	16 h/ 8 h	1 month	Shoot organogenesis	Youssef et al. 2019
	MS	3% suc.	0.7% ag	–	0.5 mg/L 2ip + 5 mg/L Pic + 1.0 mg/L BA + 0.2 mg/L NAA	23±2 °C	16 h/ 8 h	3 months	Shoots multiplication	
	MS	6% suc.	0.7% ag	3-6 mg/L pac	–	23±2 °C	16 h/ 8 h	–	Bulblet formation	
Lilium oriental hybrid cv. "Casablanca"	MS	–	–	–	0.5 mg/L BA + 0.4 mg/L BA NAA	–	–	–	adventitious bud regeneration	Javaheri and Kaviani, 2022
Lilium oriental hybrid cv. "Casablanca"	MS	9% suc.	– pg	ac	0.5 mg/L BA	25±2 °C	16 h/ 8 h	8 wks	Shoot proliferation	Han et al. 2005
**Tulip**										
T. gesneriana cv. "Apeldoorn"	MS	3% suc.	0.8% ag	–	25 or 50 μM Pic + 25 or 50 mg/L TDZ	20±2 °C	–	4 wks	Callus induction and multiplication	Ptak and Bach 2007
	MS	3% suc.	0.8% ag	–	5 μM BA + 0.5 μM NAA	20±2 °C	–	–	Plantlet regeneration	
T. gesneriana cv. "Apeldoorn"	MS	3% suc.	0.8% bag	10 mg/L thi + 1 mg/L pyr + 5 mg/L NA + 10 mg/L gly + 100 mg/L glu + 1g/L Chl	10 mg/L NAA + 0.1~0.5 mg/L TDZ	20 °C	0 h/ 24 h	60 days	Callus induction	Podwyszyńska et al. 2020
T. gesneriana cv. "Prominence"	MS	3% suc.	0.8% bag	Same to above	2.5 mg/L Pic + 0.1 mg/L TDZ	20 °C	0 h/ 24 h	60 days	Callus induction	
Tulipa gesneriana cv. "Blue Parrot"	MS	3% suc.	0.8% bag	Same to above	10 mg/L NAA	20 °C	0 h/ 24 h	60 days	Callus induction	
T. suaveolens	MS	–	–	–	0.5 mg/L BAP + 1 mg/L NAA	25±1 °C	16 h/ 8 h	2.5 months	Shoot formation	Kritskaya et al. 2019
T. tarda cv. "Stapf. "	MS	3% suc.	0.5% ag	–	0.5 μM BAP	20 °C	0 h/ 24 h	12 wks	Callus induction	Maślanka et al. 2014
	MS	6% suc.	0.5% ag	–	0.5 μM BAP	20 °C	0 h/ 24 h	12 wks	Bulb formation	
T. gesneriana cv. "Arma"	MS	3% suc.	0.75% pa	–	1.5 mg/L BA	25±2 °C	–	4 wks	Bulb formation	Ibrahim and Draaj 2020
T. gesneriana cv. "Flaming flag"	MS	3% suc.	0.75% pa	–	1.5 mg/L BA	25±2 °C	–	4 wks	Bulb formation	
**Rose**										
Rose	MS	3% suc.		400 mg/L MI	0.5 mg/L TDZ + 0.1 mg/LNAA; 0.5 mg/L ZT + 0.1 mg/L NAA	25±2 °C	16 h/ 8 h	25,45, 60 days	Flower induction	Wang et al. 2002
Hybrid *Rosa* sp.	MS	3% suc.	0.6% ag	1 mg/L GA_3	3 mg/L BAP	26±1 ºC	-	-	Shoot induction	Oo et al. 2021
	1/2 MS	1.5% suc.	0.5% ag	–	1 mg/L NAA	26±1 ºC	-	-	Root induction	
Rosa hybrida cv. Eiffel Tower	MS	3% suc.	0.8% ag	–	1 mg/L BAP + 0.5 mg/L KIN	25±2 °C	16 h/ 8 h	4 wks	Shoot multiplication	Tawfik et al. 2018
	1/2 MS	5% suc.	0.8% ag	–	–	25±2 °C	16 h/ 8 h	20 days	Root induction	
Rosa hybrida	MS	-	0.8% ag	–	3 mg/L BAP	25±3 °C	-	3 wks	Shoot induction	Tirkey et al. 2019
Rosa hybrida	MS	3% suc.	0.75% ag	0.3 g/L ac	3 mg/L BAP + 1 mg/L NAA + 0.5 mg/L GA	–	–	–	Shoot proliferation	Khaskheli et al. 2018
	MS	3% suc.	0.75% ag	0.3 g/L ac	3 mg/L IAA	–	–	–	Root induction	
Rosa hybrida cv. Red Masterpiece	MS	3% suc.	0.82% ag	–	3 mg/L BA + 1 mg/L KIN	25±1 °C	16 h/ 8 h	3 wks	Multiple shoot induction	Kanchanapoom et. al. 2009)

Rosa hybrida cv. Heirloom	MS	3% suc.	0.82% ag	–	13.3 mM BA + 9.3 mM KIN	25±1 °C	16 h/ 8 h	3 wks	Multiple shoot induction	Kanchanapoom et. al. 2010
Rosa sp.	MS	3% suc.	0.7% ag	–	4 mg/L 2,4-D	26±1 ºC	16 h/ 8 h	28-30 days	Callus induction	Afrin et al. 2022
	MS	3% suc.	0.7% ag	–	2 mg/L BAP + 0.5 mg/L KIN	26±1 ºC	16 h/ 8 h	26-28 days	Shoot regeneration	
	MS	3% suc.	0.7% ag	–	2 mg/L BAP + 0.5 mg/L KIN + 0.1 mg/L GA3	26±1 ºC	16 h/ 8 h	28 days	Shoot proliferation	
	1/2 MS	3% suc.	0.7% ag	–	1 mg/L IBA + 1 mg/L NAA	26±1 ºC	16 h/ 8 h	15-18 days	Root induction	
Rosa sp. cv. Konstancin	MS	1.9% suc.	0.3% ag	100 mg/L MI + 1 mg/L NA + 1 mg/L pyr + 1 mg/L thi + 2 mg/L gly + 10 mg/L AA	2.2 µM BAP + 0.3 µM GA_3	23±2 °C	16 h/ 8 h	3 wks	Shoot induction	Wojtania et al. 2018
	MS	1.2% suc.	0.3% ag		3.1 µM BAP + 0.9 µM GA_3 + 138 µM Fe-EDTA	23±2 °C	16 h/ 8 h	5 wks	Shoot multiplication	
Rosa indica	MS	3% suc.	–	–	2 or 4 mg/L 2,4-D	25±2 °C	16 h/ 8 h	3 wks	Callus induction	Ali et al. 2018
	MS	3% suc.	–	–	3 mg/L BAP + 3 mg/L IAA	25±2 °C	16 h/ 8 h	3 wks	Shoot regeneration	
Rosa indica	MS	–	–	–	1.5 mg/L BAP or 0.5~1 mg/L BAP + 0.5 mg/L KIN	25±2 °C	16 h/ 8 h	12 days	Shoot induction	Shabbir et al. 2009
	MS	–	–	–	0.5~1 mg/L BAP + 0.5 mg/L KIN	25±2 °C	16 h/ 8 h	12 days	Shoot multiplication	
Rose	MS	3% suc.	0.5% gr	–	3 mg/L BAP + 3 mg/L IAA	–	16 h/ 8 h	4-5 weeks	Shoot organogenesis	Chhalgri et al. 2020
Rosa sp. cv. Red Eden (climbing rose)	MS	3% suc.	0.8% ag	–	0.5 mg/L BA + 0 mg/L NAA	26±2 ºC	–	30 days	Shoot multiplication	Quynh and Pha 2020
**Damask rose**										
Rosa damascena	MS	–	–	–	4 mg/L BA + 0.1 mg/L NAA (Shoot); 0.5 mg/L NAA (Root)	–	–	4 weeks	Shoot and root induction	Maheswari et al. 2018
R. damascena cv. Azaran	mMS[3]	–	–	–	1~2 mg/L BA + 0.1 mg/L GA_3 + 0.1 mg/L NAA	23±3 °C	16 h/ 8 h	every 4 wks	Shoot proliferation and multiplication	Nikbakht et al. 2005
R. damascena cv. Ghamsar	mMS[3]	–	–	–	1~2 mg/L BA + 0.1 mg/L GA_3	23±3 °C	16 h/ 8 h	every 4 wks	Shoot proliferation and multiplication	
R. damascena	MS	0.18% suc.	0.8% ag	–	5 µM BA + 1 µM TDZ	25±1 °C	14 h/ 10 h	48 wks	Shoot organogenesis	Kumar et al 2001
R. damascena cv. Almarah	MS	3% suc.	0.8% ag	3 g/L ac	2 mg/L BA + 2 mg/L GA_3	–	–	–	Shoot organogenesis	Alsemaan 2013
R. damascena	MS	3% suc.	0.8% ag	–	2.5~3 mg/L BA + 0.1 mg/L IBA	25 ± 5 °C	16 h/ 8 h	–	Shoot organogenesis	Jabbarzadeh et al. 2005
R. damascena cv. Kashan	MS	–	–	100 mg/L AA	3 mg/L BAP	–	–	–	Shoot proliferation	Mirzaei et al. 2019
Yasooj aromatic rose	MS	–	–	100 mg/L AA	3 mg/L BAP; 1.5 mg/L BAP + 1.5 mg/L 2ip + 0.1 mg/L IBA	–	–	–	Shoot proliferation	
Kashan damask rose × Yasooj aromatic rose	MS	–	–	100 mg/L AA	3 mg/L BAP	–	–	–	Shoot proliferation	
Yasooj aromatic rose	MS	–	–	100 mg/L AA	2 mg/L NAA	–	–	–	Root induction	
**China rose**										
Rosa chinensis Jacq. var.	MS 4%	suc. – –	1.5 mg/L	TDZ 25±2 °C 16 h/ 8 h	4 wks Shoot organogenesis Tibkwang et al. 2018 *minima voss*					
**Tuberose**										
Polianthes tuberosa	MS	–	–	–	0.5 mg/L IAA + 1 mg/L KIN	25±1 °C	16 h/ 8 h	60 days	Plantlet regeneration	Ali et al. 2015
P. tuberosa	MS	–	–	–	0.1 mg/L IAA + KIN	25±1 °C	16 h/ 8 h	60 days	Plantlet regeneration	Ali et al. 2014
P. tuberosa	MS	–	–	–	0.25 mg/L BAP + 0.25 mg/L KIN	–	–	–	Shoot regeneration	Daneshvar et al. 2022
	MS	–	–	–	0.2 mg/L IBA + 0.5 mg/L NAA	–	–	–	Root induction	
P. tuberosa	MS	3% suc.	0.8% ag	–	4 mg/L BAP + 0.1 mg/L NAA	25±2 °C	16 h/ 8 h	60 days	Shoot multiplication	Singh et al. 2020

	MS	3% suc.	0.8% ag	–	1 mg/L 2,4-D + 1 mg/L NAA + 0.5 mg/L BAP or 1 mg/L 2,4-D + 2.25 mg/L BAP	25±2 °C	16 h/ 8 h	60 days	Callus induction	
	MS	3% suc.	0.8% ag	–	0.5 mg/L KIN	25±2 °C	16 h/ 8 h	60 days	Tuber regeneration	
P. tuberosa	WH	2% suc.	0.75% ag	–	0.3 mg/L TDZ + 0.5 mg/L NAA	25±2 °C	16 h/ 8 h	–	Shoot multiplication	Gajbhiye et al. 2011
	WH	1% suc.	0.75% ag	–	2 mg/L IBA	25±2 °C	16 h/ 8 h	–	Root regeneration	
P. tuberosa	WH	1.5% suc.	0.75% ag	1 mg/L GA_3	4 mg/L 2,4-D	25±2 °C	16 h/ 8 h	–	Callus induction	Raghuvanshi et al. 2013
	WH	1.5% suc.	0.75% ag	1 mg/L GA_3	1 mg/L TDZ + 0.5 mg/L NAA	25±2 °C	16 h/ 8 h	–	Shoot proliferation	
	WH	1.5% suc.	0.75% ag	1 mg/L GA_3	2 mg/L TDZ + 0.5 mg/L NAA	25±2 °C	16 h/ 8 h	–	Shoot multiplication	
	WH	1.5% suc.	0.75% ag	1 mg/L GA_3	2 mg/L IBA	25±2 °C	16 h/ 8 h	–	Root induction	
P. tuberosa	MS	3% suc.	0.8% ag	–	3 mg/L IAA + 0.5 mg/L BAP	27 °C	–	–	Callus induction	Sangavai and Chellapandi 2008
	MS	3% suc.	0.8% ag	–	1.5 mg/L BAP + 0.5 mg/L IAA	27 °C	–	–	Shoot induction	
P. tuberosa	MS	–	–	–	2 mg/L BAP + 0.5 mg/L NAA	25±2 °C	16 h/ 8 h	–	Shoot regeneration	Khanchana et al. 2019
	1/2 MS	–	–	–	1 mg/L IBA + 1 mg/L NAA	25±2 °C	16 h/ 8 h	–	Root induction	
P. tuberosa	MS	3% suc.	0.8% ag	–	4 mg/L BAP + 0.5 mg/L NAA + 0.5 mg/L TDZ	–	–	–	Shoot organogenesis and multiplication	Surendranath et al
	1/2 MS	3% suc.	0.8% ag	–	3 mg/L IBA + 1 mg/L NAA	–	–	–	Root organogenesis	
P. tuberosa	MS	2% suc.	0.64% ag	–	4.5 mg/L BAP + 0.05 mg/L NAA	–	–	–	Callus induction	Hernández-Mendoza et al. 2021
	MS	2% suc.	0.64% ag	–	4.5 mg/L BAP + 0.1 mg/L NAA	–	–	–	Shoot regeneration	
	MS	2% suc.	0.64% ag	–	1 mg/L IBAA + 0.1 mg/L BAP	–	–	–	Root induction	
**Gladiolus**										
Gladiolus grandiflorus cv. "White Friendship"	MS	3% suc.	0.8% ag	–	4 mg/L BAP	25~27 °C	16 h/ 8 h	–	Shoot regeneration	Memon et al. 2010
	MS	3% suc.	0.8% ag	–	2 mg/L IBA	25~27 °C	16 h/ 8 h	–	Root regeneration	
	MS	7% suc.	0.8% ag	–	1 mg/L IBA	25~27 °C	16 h/ 8 h	–	Cormel production	

G. grandiflorus cv. "Pink"	MS	3 % suc.	0.7 % ag	–	2 mg/L BAP + 2 mg/L NAA or 4 mg/L BAP + 1 mg/L NAA	25±1 °C	16 h/ 8 h	14 wks	Shoot regeneration	Torabi et al. 2008
				–	2 mg/L NAA	25±1 °C	16 h/ 8 h	14 wks	Root organogenesis	
				–	4 mg/L BAP + 1 mg/L NAA	25±1 °C	16 h/ 8 h	14 wks	Cormel organogenesis	
Gladiolus hybridus	MS	3% suc.	0.75% ag	–	2 mg/L BAP + 0.5 mg/L 2,4-D	25±2 °C	–	–	Callus induction	Tripathi et al. 2017
		3% suc.	0.75% ag	–	2-3 mg/L BA + 0.5 mg/L NAA	25±2 °C	–	–	Shoot proliferation	
		1.5% suc.	0.75% ag	–	0.5 mg/L IBA + 0.5 mg/L KIN	25±2 °C	–	–	Root proliferation	
G. grandiflorus cv. "White Prosperity"	MS	3% suc.	0.8% ag	–	5 mg/L BA + 0.5 mg/L IAA	25±2 °C	16 h/ 8 h	–	Shoot proliferation	Deshmukh et al. 2021
Gladiolus	MS	-	0.8% ag	–	1 mg/L BAP + 0.5 mg/L KIN	27±2 °C	16 h/ 8 h	27 days	Plantlet regeneration	Mateen 2019
	MS	-	0.8% ag	–	1 mg/L BAP + 0.5 mg/L KIN	27±2 °C	16 h/ 8 h	8 days	Shoot organogenesis	
	MS	-	0.8% ag	–	1 mg/L NAA + 0.25 mg/L KIN	27±2 °C	16 h/ 8 h	21 days	Shoot multiplication	
	MS	-	0.8% ag	–	1 mg/L NAA + 0.5 mg/L IBA	27±2 °C	16 h/ 8 h	8 days	Root organogenesis	
G. hybrida cv. "White Prosperity"	MS	3% suc.	0.7% ag	–	2 mg/L 2,4-D	25±2 °C	16 h/ 8 h	28 days	Shoot regeneration	Devi et al. 2019
G. hybrida cv. "Sylvia"	MS	3% suc.	0.7% ag	–	2 mg/L 2,4-D	25±2 °C	16 h/ 8 h	28 days		
G. hybrida cv. "Amsterdam"	MS	3% suc.	0.7% ag	–	2 mg/L 2,4-D	25±2 °C	16 h/ 8 h	28 days		
G. hybrida cv. "White Prosperity"	B5	3% suc.	0.75% ag	–	1 mg/L BAP	–	–	–	Shoot organogenesis	Kumar et al. 2018
	MS	3% suc.	0.75% ag	–	1 mg/L BAP	–	–	–		

	B5	3% suc.	0.75% ag	–	1.5 mg/L NAA + 1 mg/L BAP	–	–	–	Root organogenesis	
	MS	3% suc.	0.75% ag	–	1.5 mg/L NAA + 1 mg/L BAP	–	–	–		
**Peony**										
Paeonia suffruticosa	WPM	3% suc.	0.21% gg	–	4 mg/L IBA	25±1 °C	16 h/ 8 h	–	Shoot regeneration and root initiation	Wang et al. 2012a
**Ginger Lily/ Butterfly lily**										
Hedychium coronarium Koen.	MS	3% suc.	0.8% ag	–	3 mg/L BAP + 3 mg/L KIN + 0.2 mg/L TDZ	25±1 °C	16 h/ 8 h	–	Shoot multiplication	Parida et al. 2013
**Cyclamen** [reviewed in Jalali et al. 2012]										
Cyclamen mirabile; C. cilicium; C. pseudibericum; C. parviflorum	CIM	3% suc. + 2% glu.	0.37% gr	–	2.5 mg/L 2,4-D + 1 mg/L 2iP	25±1 °C	0 h/ 24 h	16 + 8 wks	Callus	İzgü et al. 2016
C. mirabile	1/2 MS	3% suc.	0.8% ag	–	0.1 mg/L IAA + 0.5 mg/L KIN; 0.5 mg/L NAA + 0.5 mg/L KIN	24±1 °C	16 h/ 8 h	–	Shoot and microtuber induction	Yamaner and Erdag 2008
C. persicum Mill.	MS	3% suc.	0.8% ag	–	1 mg/L NAA + 1 mg/L BA or 0.9 mg/L TDZ (shoot); 0.9 mg/L TDZ (microtuber)	22~24 °C	16 h/ 8 h	–	Shoot and microtuber induction	Abu-Qaoud 2004
**Carnation**										
Dianthus caryophyllus	MS	3% suc.	0.8% ag	–	1 mg/L BA + 2 mg/L 2,4-D	24±2 °C	16 h/ 8 h	4 wks	Callus induction	Kumar and Kumar 2009
D. caryophyllus	MS	3~4% suc.	0.7% ag	–	8.9 μM BA + 2.9 μM IAA; 10^{-3}% 24-EpiB; 10^{-5}-10^{-6}% RE	22±2 °C	16 h/ 8 h	3, 6 wks	Shoot organogenesis	Lukatkin et al. 2017
**Anthurium**										
Anthurium andreanum cv. "Jewel"	MS	3% suc.	–	–	3 mg/L 2,4-D + 0.5 mg/L BAP + 0.4 mg/L TDZ (induction); 3 mg/L NAA (compactness)	25±2 °C	16 h/ 8 h	–	Callus induction	Thokchom an Maitra 2017
	MS	3% suc.	–	–	2-3 mg/L BAP + 0.5 mg/L NAA	25±2 °C	16 h/ 8 h	–	Shoot regeneration	
	1/2 MS	3% suc.	–	–	1-2 mg/L NAA	25±2 °C	16 h/ 8 h	–	Root regeneration	
A. andreanum cv. "White Beauty"	1/2 MS	3% suc.	0.6% ag	100 mg/L MI + 250 mg/L NH_4NO_3 + 1mg/L thi + 1 mg/L BAP + 0.1 mg/L 2,4-D	1 mg/L BAP (juvenile leaf as explant)	25±5 °C	16 h/ 8 h	30 days	Shoot organogenesis	Cardoso and Habermann 2014
	MS	3% suc.	0.6% ag	–	1.82 μM TDZ	25 °C	12 h/ 12 h	–	Callus induction	Gu et al. 2012
A. andraeanum cv. "Alabama" and "Sierra"	MS	3% suc.	0.6% ag	–	0.89 μM BA + 2.32 μM KIN + 0.98 μM IBA; 0.89 μM BA + 2.32 μM KIN	25 °C	12 h/ 12 h	–	Shoot regenration	
**Zinnia**										
Zinnia elegans L.	MS	3% suc.	–	–	1 mg/L 2.4-D + 1 mg/L BA	25±2 °C	8 h/ 16 h	–	Shoot regeneration and callus induction	Ulfer et al. 2020
**Vernonia**										

Vernonia amygdalina	MS	3% suc.	0.3% gr	–	0.5~2.0 mg/L 2,4-D	25±1 °C	–	8 wks	Callus induction	Mei-Yin et al. 2018
	MS	3% suc.	0.3% gr	–	2 mg/L BAP + 2 mg/L NAA	25±1 °C	–	8 wks	Shoot multiplication	
	MS	3% suc.	0.3% gr	–	1 mg/L BAP	25±1 °C	–	8 wks	Leaf proliferation	
V. amygdalina	MS	3% suc.	0.8% ag	2 mg/L NAA	0.5 mg/L BAP + 0.5 mg/L NAA	25±2 °C	16 h/ 8 h	–	Shoot induction	Khalafalla et al. 2007
**Croton**										
Codiaeum variegatum	MS	2.5% suc.	0.25% pg	25 mg/L malt extract + 25 mg/L Pt	0.5 mg/L BAP	24±2 °C	16 h/ 8 h	6 wks	Shoot organogenesis	Nasib et al. 2008
	MS	2.5% suc.	0.25%pg	–	2 mg/L of IBA	24±2 °C	16 h/ 8 h	6 wks	Root organogenesis	
C. variegatum cv. "Coraz6n de Oro"	MS	3% suc.	0.8% ag	–	4.4 μM BA	24±1 °C	16 h/ 8 h	30 days	Shoot multiplication	Marconi et al. 1997
C. variegatum cv. "Golden Queen"	MS	3% suc.	0.6% ag	–	2 mg/L BA+ 0.2 mg/L IBA	25±2 °C	16 h/ 8 h	10, 20, and 30 days	Bud induction	Wei et al. 2019
	MS	3% suc.	0.6% ag	–	2 mg/L BA+ 0.2 mg/L NAA	25±2 °C	16 h/ 8 h	10, 20, and 30 days	Bud proliferation	
	MS	3% suc.	0.6% ag	–	1.5 mg/L IBA	25±2 °C	16 h/ 8 h	10, 20, and 30 days	Root induction	
C. variegatum cv. "Petra"	MS	6% suc.	0.8% ag	–	2 mg/L NAA	25±1 °C	16 h/ 8 h	45 days	Callus induction	Bakheet et al. 2018
C. variegatum cv. "Gold Dust"	MS	6% suc.	0.8% ag	–	1 mg/L BAP + 25 mg/L Peptone	25±1 °C	16 h/ 8 h	45 days	Callus induction	
**Chrysanthemum**										
Dendranthema morifolium	MS	–	0.8% ag	0.2 mg/L IBA	0.5~1.0 mg/L NAA + 1.0~1.5 mg/L BAP	25±1 °C	16 h/ 8 h	6 weeks	Shoot multiplication	Waseem et al. 2011
Chrysanthemum cv. "Vivid Scarlet"	MS	–	0.3% gr	1 g/L ac	1 mg/L BA + 2 mg/L NAA	–	16 h/ 8 h	45 days	Shoot formation	Naing et al. 2014
Chrysanthemum cv. "Shinma"	MS	–	0.3% gr	–	0.5 mg/L BA + 0.5 mg/L NAA	–	16 h/ 8 h	45 days	Shoot formation	Naing et al. 2016
C. morifolium cv. "Resomee Splendid"	MS	3% suc.	0.65% ag	–	4.5 mg/L BAP + 1 mg/L NAA (leaf as explant)	25±2 °C	16 h/ 8 h	7 weeks	Shoot organogenesis	Kazeroonian et al. 2018
	MS	3% suc.	0.65% ag	–	1.5 mg/L BAP + 1 mg/L NAA (petiole as explant)	25±2 °C	16 h/ 8 h	7 weeks		
**Campsis**										
Campsis radicans (L.) Seem.	MS	3% suc.	0.65% ag	–	2.5~5 mg/L IAA or IBA	20±2 °C	16 h/ 8 h	4 weeks	Root formation	Parzymies et al. 2014
**Brunfelsia**										
Brunfelsia calycina	MS	3% suc.	0.75% ag	–	1 mg/L BA + 0.6 mg/L IAA	–	16 h/ 8 h	–	Shoot regeneration	Liberman et al. 2010
**Bougainvillea**										
Bougainvillea × buttiana	WPM	3% suc.	0.7% ag	–	2 mg/L BA + 0.2 mg/L NAA.	25±2 °C	16 h/ 8 h	4 weeks	Shoot regeneration	Duhoky and Al-Mizory, 2014
**Bottle Brush**										

Callistemon citrinus	WPM	3% suc.	0.8% ag	100 mg/L MI + 1 mg/L thi + 0.5 mg/L pyr + 0.5 mg/L NA	0.5 mg/L BA + 0.25 mg/L NAA	25 °C	16 h/ 8 h	–	Shoot proliferation	Papafotiou and Skylourakis, 2010
**Petunias**										
Petunia hybrida Vilm. cv. "Bravo"	MS	3% suc.	–	MI	1.5 mg/L TDZ + 1 mg/L BAP	24±1 °C	16 h/ 8 h	–	Callus induction and	Farooq et al. 2021
	MS	3% suc.	–	MI	0.5 mg/L IBA + 0.5 mg/L BAP	24±1 °C	16 h/ 8 h	–	Shoot regeneration	
Petunia hybrida	MS	–	–	–	0.6 mg/L BAP + 0.5 mg/L IBA	–	–	–	Shoot multiplication	Habas et al. 2019
**Periwinkle**										
Catharanthus roseus Don	MS	3% suc.	0.7% ag	100 mg/L MI	3 μM BA + 3 μM KIN + 0.1 μM AgNO3	25±1 °C	12 h/ 12 h	12 days	Shoot formation	Panigrahi et al. 2018
Catharanthus roseus Don	MS	3% suc.	0.7% ag	–	1 mg/L IAA + 4 mg/L BA	–	16 h/ 8 h	4 wks	Callus induction	Hoda 2013
	MS	3% suc.	0.7% ag	–	4 mg/L BA	–	16 h/ 8 h	–	Shoot formation	
	MS	3% suc.	0.7% ag	–	1 mg/L IAA + 4.0 mg/L BA	–	16 h/ 8 h	–	Root formation	
**Hibiscus**										
Hibiscus coddii subsp. *barnardii*	MS	3% suc.	0.3% gr	–	1 mg/L BAP	24±2 °C	16 h/ 8 h	–	Callus induction	Plessis et al. 2021
H. syriacus	MS	3% suc.	0.3% gr	–	0.01 mg/L TDZ	25±1 °C	16 h/ 8 h	–	Shoot regeneration	Seo et al. 2017
	MS	3% suc.	0.3% gr	–	1 mg/L BAP	25±1 °C	16 h/ 8 h	–	Callus induction	
**Begonia**										
Begonia rubrovenia var. *meisneri* C.B. Clarke	MS	3% suc.	0.8% ag	–	0.1 mg/L TDZ (shoot); 0.1 mg/L IAA (root)	25±2 °C	12 h/ 12 h	–	Plantlet regeneration	Kumaria et al. 2012
**Coleus**										
Coleus aromaticus Benth	MS	–	–	–	1.0 mg/L BAP + 40 mg/L Ch (shoot); 0.5 mg/L NAA (root)	24±2 °C	16 h/ 8 h	–	Shoot and root organogenesis	Govindaraju and Arulselvi 2018
**Hosta**										
Hosta sieboldiana	MS	3% suc.	0.2% gg	–	0.5~1 mg/L Pic	25 °C	16 h/ 8 h	6 wks	Callus induction	Saito and Nakano 2002
H. sieboldiana	MS	3% suc.	0.2% gg	0.5-1 mg/L Pic	0.1 mg/L NAA + 1 or 5 mg/L TDZ	25 °C	16 h/ 8 h	2 months	Plantlet regeneration	
H. capitata (Koidz.) Nakai	MS	3% suc.	0.7% ag	–	2 mg/L TDZ	–	–	–	Shoot multiplication	Choi et al. 2019
H. clausa Nakai and *H. jonesii* M.G.Chung	MS	3% suc.	0.7% ag	–	1 mg/L TDZ	–	–	–	Shoot multiplication	
H. minor (Baker) Nakai	MS	3% suc.	0.7% ag	–	0.5 mg/L BA	–	–	–	Shoot multiplication	
H. venusta F.Maek.	MS	3% suc.	0.7% ag	–	1 mg/L BA; 0.1 mg/L TDZ	–	–	–	Shoot multiplication	
H. yingeri S.B.Jones	MS	3% suc.	0.7% ag	–	0.5 mg/L TDZ	–	–	–	Shoot multiplication	
H. capitata	MS	3% suc.	–	–	3 mg/L BA + 0.1 mg/L NAA	–	16 h/ 8 h	6wks	Shoot regeneration	Pe et al. 2020
H. capitata	MS	3% suc.	–	3 mg/L BA + 0.1 mg/L NAA	20 mg/L NAg	–	16 h/ 8 h	6wks	Plant growth	
H. plantaginea'Joseon'	MS	3% suc.	0.8% ag	0.1 mg/L MI	4 mg/L BA + 0.2 mg/L NAA	23±1 °C	16 h/ 8 h	6wks	Plantlet regeneration	Ku et al. 2016

H. plantaginea'Joseon'	MS	3% suc.	0.8% ag	0.1 mg/L MI	2 or 4 mg/L KIN + 0.2 mg/L-1 NAA	23±2 °C	16 h/ 8 h	6wks	Plantlet regeneration	
H. minor (Baker) Nakai	MS	3% suc.	0.8% ag	–	2.0 μM TDZ + 0.5 μM NAA	–	–	–	Shoot multiplication	Song et al. 2020
H. minor (Baker) Nakai	1/2 MS	3% suc.	0.8% ag	–	2 μM IBA	–	–	–	Root organogenesis	
**Bauhinia**										
Bauhinia racemosa Lam.	MS	3% suc.	0.8% ag	50 mg/L AA + 25 mg/L AS **+** 25 mg/L **CA** + 25 mg/L L–arg	3 mg/L BA	26±2 °C	–	30–35 days	Shoot induction	Sharma et al. 2017
	MS	3% suc.	0.8% ag		0.75 mg/L BA	26±2 °C	–	30–35 days	Shoot multiplication	
**Morning glory**										
Ipomoea purpurea	MS	3% suc.	0.7% ag	–	0.25~0.50 mg/L IBA	23±1 °C	16 h/ 8 h	–	Root organogenesis	Acemi et al. 2018
Merremia quinquefolia	MS	3% suc.	0.8% ag	–	4.0 mg/L BAP	25±2 °C	16 h/ 8 h	45 days	Shoot multiplication	Kher et al. 2015
M. quinquefolia	1/2 MS	3% suc.	–	–	2 mg/L IBA	25±2 °C	16 h/ 8 h	45 days	Shoot multiplication	
**Common laburnum**										
Laburnum anagyroides	MS	2% suc.	–	–	2.22 μM BAP	24±2 °C	14h /10h	–	Shoot organogenesis	Timofeeva et al. 2014
L. anagyroides	1/2 MS	1% suc.	–	–	2.22 μM BAP	24±2 °C	14h /10h	–	Shoot multiplication	

**Lanterns**										
Ceropegia attenuata Hook	MS	3% suc.	0.2% gr	–	13.31 μM BAP	25±2 °C	16 h/ 8 h	4 wks	Shoot induction and multiplication	Chavan et al. 2011
C. bulbosa var. bulbosa	MS	3% suc.	0.8% ag	–	8.88 μM BAP + 0.54 μM NAA	25±2 °C	16 h/ 8 h	4 wks	Shoot induction and multiplication	Dhir and Shekhawat 2014
C. bulbosa var. *lushii*	MS	3% suc.	0.8% ag	–	8.88 μM BAP + 0.27 μM NAA	25±2 °C	16 h/ 8 h	4 wks		
C. bulbosa var. bulbosa	MS	3% suc.	0.8% ag	–	8.88 μM BA	25±2 °C	16 h/ 8 h	4 wks	Shoot multiplication	Dhir and Shekhawat 2013
C. elegans	MS	3% suc.	0.8% ag	–	23.2 μM KIN + 5.71 μM IAA	25±2 °C	16 h/ 8 h	4 wks	Shoot multiplication	Krishnareddy and Pullaiah 2012
C. ensifolia	MS	3% suc.	0.8% ag	–	3 mg/L BAP + 0.2 mg/L TDZ	25±2 °C	16 h/ 8 h	6 wks	Shoot multiplication	Reddy et al. 2015
	MS	3% suc.	0.8% ag	–	0.1 mg/L NAA + 0.05 mg/L BAP	25±2 °C	16 h/ 8 h	6 wks	Root organogenesis	
C. evansii	MS	3% suc.	0.8% ag	–	4 mg/L BAP + 0.3 mg/L IAA	25±1 °C	16 h/ 8 h	–	Shoot regeneration and multiplication	Chavan et al. 2015
	MS	3% suc.	0.8% ag	–	2 mg/L NAA + 1.5 mg/L IBA	25±1 °C	16 h/ 8 h	–	Root organogenesis	
C. noorjahaniae	MS	3% suc.	0.2% cg	–	2 mg/L BAP	25±1 °C	16 h/ 8 h	4 wks	Shoot induction and multiplication	Chavan et al. 2014
	1/2 MS	3% suc.	0.2% cg	–	1 mg/L IAA	25±1 °C	16 h/ 8 h	4 wks	Root organogenesis	
C. panchganiensis	MS	3% suc.	0.2% cg	–	13.31 μM BAP + 2.69 μM NAA	25±1 °C	16 h/ 8 h		Shoot induction and multiplication	Chavan et al. 2013
	MS	3% suc.	0.2% cg	–	9.05 μM 2,4-D	25±1 °C	16 h/ 8 h	–	Callus induction	
	1/2 MS	175 mM suc.	–	–	4.44 μM BAP (flower bud); 17.74 μM BAP (microtuber); 7.36 μM IBA (root)	25±1 °C	16 h/ 8 h	–	Flower bud, microtuber formation, and rooting	

**Dracaena/ Snake plant**										
Dracaena sanderiana Sander ex Mast	MS	–	–	–	6.78 µM 2,4-D (callus); 7.84 µM BAP (shoot); 7.38 µM IBA (root)	25±2 °C	16 h/ 8 h	–	Callus, shoot and root induction	Aslam et al. 2013
**Hyacinths**										
Lachenalia viridiflora (Turquoise hyacinth)	MS	3% suc.	–	0.1 g/L MI	2.5 µM Pic + 1 µM TDZ	25±2 °C	16 h/ 8 h	6 wks	Embryogenic callus induction	Kumar et al. 2016
	MS	3% suc.	–	0.1 g/L MI	5 µM phloroglucinol	25±2 °C	16 h/ 8 h	6 wks	Plantlet regeneration	
Hyacinthus orientalis subsp. *chionophyllus* Wendelbo	MS	3% suc.	0.7% ag	2 mg/L BA + 0.5 mg/L IAA	1 mg/L JA + 0.25 mg/L 2iP	24±2 °C	16 h/ 8 h	12 wks	Bulb formation	Doğan et al. 2020
	MS	3% suc.	0.7% ag	2 mg/L BA + 0.5 mg/L IAA	2 mg/L JA + 1 mg/L IAA	24±2 °C	16 h/ 8 h	12 wks	Bulb formation	
**Dumb cane**										
Dieffenbachia cv. "Camouflage", "Camille", "Octopus", "Star Bright"	MS	–	0.6% ag	–	5 µM TDZ + 1 µM 2,4-D	22±3 °C	16 h/ 8 h	8 wks	Callus	Shen et al. 2008
	MS	–	0.6% ag	–	80 µM 2iP + 2 µM IAA	22±3 °C	16 h/ 8 h	8 wks	Shoot organogenesis	
**Ixora**										
Ixora coccinea	MS	–	–	0, 0.5, 2 and 4 mg/L Kinetin and Zeatin	0, 0.5, 2 and 4 mg/L of different type of cytokinins BAP or 0.5 mg/L IBA	25±2 °C	–	8 wks	Shoot multiplication	Onsa et al. 2018
**Rhododendron**										
Rhododendron fortunei	WPM	1.5% suc.	0.7% ag	4 mg/L 6P or ZT	1.0 mg/L NAA	25 °C	116 h/ 8 h	90 days	Axillary shoot induction	Wei et al. 2018
**Lantena**										
Lantana camara L	MS	–	–	–	12.0e20 mM TDZ (shoot), 5 or 10 mM NAA (root), 21.5 mM NAA and 22.5 mM BA (callus)	25±2 °C	16 h/ 8 h	4 wks	Shoot multiplication, callus and root induction	Veraplakorn et al. 2018
**Oleander**										
Nerium oleander L.	MS	3% suc.	0.7% ag	–	8.8 µM TDZ or 4.4 µM BA	25±2 °C	16 h/ 8 h	45 days	Shoot proliferation	Vila et al. 2010
**Jasmine**										
Jasminum grandiflorum L.	MS	–	–	–	1 mg/L BAP + 45 mg/L cw	25±2 °C	16 h/ 8 h	4 wks	Shoot proliferation	Rahman et al. 2018
	MS	–	–	–	1.5 mg/L IBA	25±2 °C	16 h/ 8 h	14–23 days	Rooting	
**Passion Flower**										
Passiflora caerulea L	MS	–	–	–	2 mg/L BAP + 1 mg/L KIN	26±1 °C	16 h/ 8 h	54 days	Direct shoot induction	Rathod et al. 2014
**Honey Suckle**										
Lonicera japonica "Japanese honeysuckle"	DKW	3% suc.	0.7% ag	100 mg/L MI	5 µM BA	25 °C	16 h/ 8 h	12 wks	Axillary micro shoots	Osburn et al. 2009
L. japonica "Amur honeysuckle"	MS	3% suc.	0.7% ag	100 mg/L MI	2.5 µM BA ± 1.25 µM IBA	25 °C	16 h/ 8 h	4 wks	Shoot proliferation	

**Heliconia**										
Heliconia rostrata and *H. bihai*	MS	6% suc.	0.8% ag	0.25% ac	1 mg/L BAP + 1 mg/L GA_3	27±1 °C	16 h/ 8 h	45 days	Callus formation	Souza et al. 2008
**Angelonia**										
Agelonia integerrima MS 2019	3% suc.	0.7% ag –	1 mg/L	BAP + 0.1 mg/L NAA	– – – Multiplication Winhelmann et al.					
**Gardenia**										
Gardenia jasminoides Ellis	1/2 MS	3% suc.	0.7% ag	–	1 mg/L BA	–	–	6 wks	Shoot multiplication	Amer et al. 2019
**Gerbera**										
Gerbera jamesonii Bolus	MS	3% suc.	0.8% ag	1 mg/L KIN	1 mg/L BA	25±2 °C	16 h/ 8 h	4 wks	Shoot regeneration	Minerva et al. 2012
**Treasure Flower**										
Gazania rigens	MS	3% suc.	–	–	0.2 mg/L BA + 1.0 mg/L IAA (100%), or<br>0.5 mg/L BA + 1.0 mg/L IAA (98%), or<br>0.5 mg/L BA + 0.5 mg/L IAA (97%), or<br>1 mg/L BA + 1 mg/L IAA (97%), or<br>1.0 mg/L BA + 0.5 mg/L IAA (98%), or<br>0.5 mg/L BA (97%)	25±2 °C	16 h/ 8 h	–	Callus induction	Yalcın-Mendı et al. 2009
**Cypress Vine**										
Ipomoea quamoclit	MS	3 % suc.	0.7 % ag	0.3 mg/L KIN	0.3 mg/L BAP	23±2 °C	16 h/ 8 h	–	F lower bud induction	Haque et al. 2013
**Bleeding Heart**										
Lamprocapnos spectabilis Fukuhara	mMS[4]	3% suc.	0.8% ag	–	1.11 μM BA + 1.23 μM IBA	23±1 °C	16 h/ 8 h	10 wks	Axillary bud activation	Kulus and Miler 2021
**Coral Vine**										
Antigonon leptopus	MS	–	0.7% ag	1 mg/L 2ip	0.2 mg/L IBA	24±2 °C	16 h/ 8 h	4 wks	Shoot formation	Ghareeb and Taha 2018
	MS	–	0.7% ag	1 mg/L 2ip	0.5 mg/L + 0.2 mg/L IBA or 1 mg/L + 0.2 mg/L IBA	24±2 °C	16 h/ 8 h	4 wks	Shoot multiplication	
**Amaranthus**										
Amaranthus	MS	3% suc.	0.6% ag	–	1.5 mg/L NAA	26±2 °C	16 h/ 8 h	–	Rooting	Tour et al. 2020
	MS	3% suc.	0.6% ag	–	1.5 mg/L BA + 0.1 mg/L NAA	27±2 °C	16 h/ 8 h	–	Shoot multiplication	
	MS	3% suc.	0.6% ag	–	1.5 mg/L BA + 0.5 mg/L NAA	26±2 °C	16 h/ 8 h	4 wks	Direct shoots initiation	
**Alocasia**										
Alocasia amazonica, A. cuprea, A. robusta, A. longiloba and A. chaii	MS	3% suc.	0.75% ag	100 mg/L MI	2 mg/L BA	23±2 °C	24 h/ 0 h	4 wks	Shoot proliferation.	Bhatt et al. 2013
**Silver Nerve Plant**										
Fittonia Albivenis (Lindl. ex Veitch) Brummitt	1/2 MS	–	–	–	1.5 or 0.5 mg/L	25 °C	16-h	–	Shoot formation	Belokurova et al. 2019
**Money plant**										

Epipremnum aureum "Linden" and "Andre"	MS	2% suc.	0.6% ag.	1, 5 or 10 μ*M* TDZ	0.5 or 1.0 μM NAA	25 °C	16 h/ 8 h	45 days	Shoot regeneration	Qu et al. 2002
Epipremnum aureum "Älbino"	MS	0.25% suc.	5 g/L ag	2 mg/L CPPU, 10 mg/L AA	0.2 mg/L NAA	23 °C	24 h/ 0 h	37 days	Chorophyll development	Hung et al. 2021
**Spider plant**										
Chlorophytum borivilianum Sant. Et Fernand	MS	–	–	20 mg/L $AdSO_4$	2 mg/L BAP + 1 mg/L NAA	–	–	45 days	shoot induction and shoot proliferation	Khatri et al. 2019
**Common rue**										
Ruta graveolens	MS	3% suc.	1% ag	–	10 μM BA + 2.5 μM NAA	25±2 °C	16 h/ 8 h	8 wks	Shoot regeneration	Faisal et al 2018
R. graveolens	MS	3% suc.	0.8% ag	–	11 μM BA + 2.5 μM NAA	24±3 °C	16 h/ 8 h	8 wks	Shoot regeneration	Faisal et al 2005
**Mezereum**										
Daphne mezereum "Alba"	MS	Suc.	ag	MI	1 mg/L BA + 0.1 mg/L NAA; 0.3 mg/L GA3 + 1 mg/L mT	–	–	6 wks	Shoot organogenesis	Nowakowska et al. 2019

Table S2. Effective elicitors for the in vitro culture in ornamentals.

**Plant species**	**Media**	**Carbon source**	**Solidifying agent**	**Media supplements**	**Effective concentration and combinations**	**Temperature (°C)**	**Light /dark cycle**	**Culture period**	**Target of regeneration**	**References**
**Cymbidium**										
Cymbidium insigne	mMS[1]	2% suc.	0.22% pg	–	1 mg/L 5-ALA		16 h/ 8 h	8 wks	PLB regeneration	Nahar and Shimasaki 2014
C. insigne	mMS[1]	2% suc.	0.2% pg	–	0.1 mg/L MeJA or 0.1 mg/L Lysozyme	25 **°C**	16 h/ 8 h	6 wks	PLB regeneration	Nahar et al. 2015
C. finlaysonianum	mMS[1]	2% suc.	0.22 % pg	–	0.01 mg/L 5-ALA		16 h/ 8 h			
Cymbidium Twilight Moon 'Day Light'	TC	2% suc.		0.1 mg/L NAA + 0.1 mg/L KIN + 2 g/L Tt	1-2 mg/L $AgNO_3$			60 days	PLB	da Silva 2013
**Dendrobium**										
Dendrobium sp	VW	2% suc.	–	50 ml/L cw, 0.525 g/L KNO_3, 0.25 g/L KH_2PO_4, 0.5 g/L $(NH_4)_2S0_4$, 0.007 g/L $MnSO_4.4H_2O$, 0.025 g/L $MgS0_4.7H_20$, 0.028 g/L $FeS0_4.7H_20$ and 0.037 g/L Na_2- EDTA, 10 mg/L auxin, 5 mg/L cytokines and 2 mg/L 2,4-D	15 ppm of Ch	–	–	3, 6 and 9 wks	PLB organogenesis	Restanto et al 2016
Dendrobium sp cv. 'Eiskul'	mVW	2% suc.	0.8% ag	15% cw	10 or 20 mg/L of 80% O-Ch (PLBs); 10 mg/L of 80% O-Ch or 80% P-Ch (plantlets)	25±2 **°C**	16 h/ 8 h	3 months	PLB-shoot induction	Pornpienpakdee et al. 2010
D. phalaenopsis	VW	2% suc.	1.2% ag	15% cw + 0.525 gL KNO_3 + 0.25 g/L KH_2PO_4 + 0.5 g/L $(NH_4)_2SO_4$ + 0.007 g/L $MnSO_4{\cdot}4H_2O$ + 0.025g/L $MgSO_4{\cdot}7H_2O$ + 0.028 g/L $FeSO_4{\cdot}7H_2O$ + 0.037 g/L Na_2-EDTA	15 ppm (PLB) or 20 ppm (plantlet) of 1 kDa O-Ch-shrimp	27 **°C**	16 h/ 8 h	12 wks	PLB and plantlet regeneration	Nge et al. 2006
D. bigibbum var. *compactum*	mVW	2% suc.	0.8% ag	15% cw	10 mg/L O-Ch/P-Ch (70%, 80%, and 90%, except 90% O-Ch)	25 **°C**	16 h/ 8 h	12 wks	PLB organogenesis	Kananont et al. 2010

D. formosum	mVW	2% suc.	0.8% ag	15% cw	10 or 20 mg/L of 70% P-Ch	25 °C	16 h/ 8 h	12 wks		
D. thyrsiflorum	1/2 MS	–	–	2 mg/L IBA + 0.6 mg/L NAA	0.5 mg/L PG	25±1 °C	12 h/ 12 h	5 wks	Rooting	Bhattacharyya et al. 2015
**Phalaenopsis**										
Phalaenopsis cv. 'Fmk02010'	mMS[1]	2% suc.	0.22% pg	–	0.01 mg/L CCC	25±2 °C	16 h/ 8 h	42 days	PLB regeneration	Mehraj et al. 2019
Phalaenopsis cv. 'Fmk02010'	mMS[1]	2% suc.	0.2% pg	–	0.1 mg/L HA9 + HA12	25±2 °C	16 h/ 8 h	42 days	PLBs proliferation	Mehraj and Shimasaki 2017
Phalaenopsis cv. 'Fmk02010'	mMS[1]	2% suc.	0.2% pg	–	0.1 mg/L HA9; 0.1 mg/L HA12	25±2 °C	16 h/ 8 h	42 days	PLBs proliferation	Sultana et al. 2015a
Phalaenopsis cv. 'Fmk02010'	mMS[1]	2% suc.	0.2% pg	–	0.1 mg/L NAG; 0.1 mg/L 5-ALA	25±2 °C	16 h/ 8 h	6 wks	PLBs proliferation	Sultana et al. 2015b
**Cattleya**										
Cattleya loddigesii	KC		0.5% ag	–	5 mL/L K_2SiO_3 + 20 mg/L Na_2SiO_3	25±1 °C	16 h/ 8 h	120 days	Rooting	Soares et al. 2011
**Rose**										
Rosa x *hybrida* cv. Sena	1/2 MS	3% suc.		0.1 g/L MI + 0.5 mg/L BA	2 mg/L $AgNO_3$			20 , 60 days	Flower induction	Matos et al. 2020
Rosa sp cv. Red Eden (climbing rose)	1/2 MS	3% suc.	0.8% ag	–	30 μM $AgNO_3$	26±2 °C	–		Flower and root induction	Quynh and Pha 2020
**Anthurium**										
Anthurium andraeanum cv. Alabama and Dakota	1/2 MS	3% suc.		0.1 g/L MI + 0.5 mg/L KIN + 1.5 g/L ac	1 mg/L $AgNO_3$			60 days	Shoot multiplication	Cardoso 2019
	1/2 MS	3% suc.		0.1 g/L MI + 0.1 mg/L IBA + 1.5 g/L ac	1-2 mg/L $AgNO_3$			60 days	Rooting	
**Morning glory**										
Ipomoea purpurea	MS	3% suc.			5 mg/L Ch (mixture O-Ch-DP 2-15)			–	Shoot induction and growth	Acemi et al. 2018
**Periwinkle**										
Catharanthus roseus	MS	3% suc.		100 mg/L MI	3 μM BA + 3 μM KIN + 0.1 μM $AgNO_3$			–	Plantlet regeneration and flower induction	Panigrahi et al. 2018
**Tulip**										
Tulipa gesneriana cv. New Beet	1/2 MS	7% suc.	0.1% ac	–	100 μM Pu + 100 μM Spe + 5 mL of 0.1% MeJA	16 °C	16 h/ 8 h	13 wks	Bulb formation	Podwyszyńska et al. 2015
T. gesneriana cv. New Beet	1/2 MS	7% suc.	0.1% ac	–	50 μM Pu + 5 mL of 0.1% MeJA	16 °C	16 h/ 8 h	13 wks	Bulb formation	
T. gesneriana cv. Fringed Black	1/2 MS	7% suc.	0.1% ac	–	100 μM Pu + 5 mL of 0.1% MeJA	16 °C	16 h/ 8 h	13 wks	Bulb formation	
T. gesneriana cv. P14 (late flowering, Lily-flowered Group)	1/2 MS	7% suc.	0.1% ac	–	500 μM Orn + 5 mL of 0.1% MeJA	16 °C	16 h/ 8 h	13 wks	Bulb formation	

T. gesneriana cv. P20 (meadseason flowering, Fringed Group)	1/2 MS	7% suc.	0.1% ac	–	100 μM Pu + 5 mL of 0.1% MeJA or 100 μM Spe + 5 mL of 0.1% MeJA	16 °C	16 h/ 8 h	13 wks	Bulb formation	
**Hyacinths**										
Hyacinthus orientalis subsp. *chionophyllus* Wendelbo	MS	3% suc.	0.7% ag	2 mg/L BA+ 0.5 mg/L IAA	1 mg/L JA + 0.25 mg/L 2iP	24±2 °C	16 h/ 8 h	12 wks	bulb formation	Doğan et al. 2020
	MS	3% suc.	0.7% ag	2 mg/L BA+ 0.5 mg/L IAA	2 mg/L JA + 1 mg/L IAA	24±2 °C	16 h/ 8 h	12 wks	bulb formation	

Table S3. Effective organic additives for the in vitro culture in ornamentals.

Plant species	Media	Carbon source	Solidifying agent	Media supplements	Effective factors	Temperature (°C)	Light /dark cycle	Culture period	Target of regeneration	Ref
**Phalaenopsis orchid**										
Phalaenopsis Hybrid 'Pink'	MS	2% suc.	–	–	10% carrot juice	–	15 h/ 7 h	4 months	Plantlet regeneration	Zahara et al. 2017
	VW	2% suc.	–	–	20% carrot juice	–	15 h/ 7 h	4 months	Plantlet regeneration	
P. amabilis 'Blume'	NP	–	–	–	15% tomato extract	25±1 °C	0 h/ 24 h (1st 14 days); 16 h/ 8 h	12 wks	somatic embryogenesis and plantlet generations	Mose et al. 2020
P. amabilis BL. cv. 'Golden Horizon	1/2 MS	2% suc.	0.22% gr	10% cw + 2 g/L peptone + 1 g/L ac + 150 mg/L L-glutamone	2.5 g/L banana powder	24±1 °C	16 h/ 8 h	4, 6, and 8 wks	Rooting	Sinha and Jahan 2011
Bulbophyllum auricomum Lindl.	MS	3% suc.	0.8% ag	–	15% coconut water (cw)	25±2 °C	16 h/ 8 h	1 and 2 months	Callus induction and PLB proliferation	Aung et al. 2022
Paphiopedilum rothschildianum	1/2 MS	3% suc.	3% gr	–	60 g/L BH	25±2 °C	0 h/ 24 h	8 wks	PLB proliferation	Ng et al. 2012
P. rothschildianum	1/2 MS	3% suc.	3% gr	–	30% cw	25±3 °C	1 h/ 24 h	9 wks	PLB proliferation	
**Dendrobium orchid**										
Dendrobium macrostachyum	MS	3% suc.	0.8% ag	–	15% cw	25±2 °C	–	10 wks	Shoot regeneration	Pyati et al. 2002
D. officinale	MS	3% suc.	0.55% cn	2 mg/L BA + 0.1 mg/L NAA	100 g/L potato extract	26±2 °C	12 h/ 12 h	6 days	Shoot multiplication	Chen et al. 2014
D. densiflorum Lindl.	MS	3% suc.	0.8% ag	0.01 g/L MI	15% cw	25±2 °C	16 h/ 8 h	–	PLB proliferation and shoot development	Pant et al. 2022

Table S4. Effective light emitting diodes (LEDs) for the in vitro culture in ornamentals.

Plant speces	Effective LEDs	Media	Carbon source	Solidifying agent	Media supplements	Temperature (°C)	Light /dark cycle	Clture period	Organogenesis/ regenaration	Ref
**Cymbidium orchid**										
Cymbidium "Twilight Moon "Day Light"	100% red	NN	2% suc.	0.8% bag	0.1 mg/L NAA + 0.01 mg/L TDZ	–	16 h/ 8 h	1 month	Callus proliferation	Le and Tanaka 2004
	25% red +75% blue	NN	2% suc.	0.8% bag	0.1 mg/L NAA + 0.01 mg/L TDZ	–	16 h/ 8 h	1 month	PLBs formation	
Cymbidium "Golden Bird"	Red light (1:0)	VW	2% suc.	–	2 g/L tryptone + 1 ml/L NN microelements	25±1 °C	16 h/ 8 h	–	Plantlet regeneration	Tanaka et al. 1998
Cymbidium Waltz 'Idol'	Red + green	mMS[1]	2% suc.	0.2% pg	–	25±2 °C	16 h/ 8 h	6 wks	PLB regeneration	Kaewjampa and Shimasaki 2012
Cymbidium (eight hybrids)	Different ratios (1:0; 1.5:1; 1:1; 1:1.5; 0:1) of red and blue	TC1	2% suc.	0.8% bag	0.1 mg/L NAA + 0.1 mg/L KIN + 2 g/L tryptone	25±1 °C	16 h/ 8 h	–	PLB regeneration	da Silva 2014b
**Phalaenopsis orchid**										
Phalaenopsis cv. 'Fmk02010'	Red → white	mMS[1]	2% suc.	0.22% pg	–	25±2 °C	16 h/ 8 h	42 days	PLB regeneration	Mehraj et al. 2019
	Blue → white	mMS[1]	2% tre.	0.22% pg	–	25±2 °C	16 h/ 8 h	42 days	PLB regeneration	
	Red → green → white	mMS[1]	2% mal.	0.22% pg	–	25±2 °C	16 h/ 8 h	42 days	PLB regeneration	
Phalaenopsis Hybrid	80% red + 20% blue	VW	1 % suc.	–	–	–	–	4 months	PLB organogenesis	Wongnok et al. 2008
	90% red + 10% blue				–	–	–	–	Shoot regeneration	
Phalaenopsis	White or blue + red	MS	–	0.25% pg	PGRs supplemented (not specified)	24±2 °C	16 h/ 8 h	60 days	Plantlet regeneration	Bello-Bello et al. 2017
**Dendrobium orchid**										
Dendrobium officinale	Different ratios (1:0; 0:1; 1:1; 2:1; 1:2) of red and blue	1/2 MS	3% suc.	0.7% ag	0.5 g/L NAA, 0.2 g/L 6-BA,	25±2 °C	16 h/ 8 h	30, 60, and 90 days	PLB regeneration	Lin et al. 2011
D. sonia	Yellow	MS	3% suc.	0.6% ag	11.1 μM BAP + 11.42 μM IAA	22±2 °C	16 h/ 8 h	4 wks	Shoot and PLB regeneration	Billore et al. 2017
D. sonia	Yellow	MS	3% suc.	0.6% ag	2.5 mg/L BAP + 2 mg/L IAA	22~25 °C	16 h/ 8 h	–	Plantlet regeneration	Billore et al. 2019
D. kingianum	Blue	mMS[1]	2% suc.	0.22% pg	–	25±1 °C	16 h/ 8 h	4 wks	PLB regeneration	Habiba et al. 2014
D. okinawense	Red	mMS[1]	2% suc.	0.22% pg	–	25±1 °C	16 h/ 8 h	42 days	PLB proliferation	Mehbub et al. 2022
**Cattleya orchid**										
Cattleya Lindl. hybrid Red, NAA al. 2007	blue MS 3% suc. 0.8% bag 2	mg/L	AS + 5	mg/L BA	+ 0.2 mg/L ZT + 1 mg/L 23±1 °C 16 h/ 8 h 3	months	Plantlet		regeneration Cybularz-Urban et	
**Oncidium orchid**										
Oncidium "Gower Ramsey"	Red	MS	3% suc.	0.6% ag	0.1 g/L BA + 0.5 mg/L NAA	25±2 °C	16 h/ 8 h	30 days	PLB regeneration	Mengxi et al. 2011
**Orchids in other genus**										
Paphiopedilum delenatii	Blue	SH	3% suc.	0.9% ag	1 mg/L TDZ + 0.3 mg/L NAA	25±3 °C	16 h/ 8 h	4 months	Shoot regeneration	Luan et al. 2015
Bletilla ochracea Schltr.	Green, oranage	NDM	2% suc.	0.2% pg	–	25±2 °C	16 h/ 8 h	3 wks	Seed germination	Godo et al. 2011
B. ochracea Schltr.	Red, oranage	NDM	2% suc.	0.2% pg	–	25±2 °C	16 h/ 8 h	4 wks	Rhizoid	
B. ochracea Schltr.	White, blue	NDM	2% suc.	0.2% pg	–	25±2 °C	16 h/ 8 h	3 months	Plantlet regeneration	
B. ochracea Schltr.	White, blue	NDM	2% suc.	0.2% pg	–	25±2 °C	16 h/ 8 h	3 months	Pseudobulb	
Calanthe	Red + blue		1.5% & 3% suc.	0.2% ag	–	25±2 °C	16 h/ 8 h	8 wks	Plantlet regeneration	Baque et al. 2011
Doritaenopsis	Red + blue	mHm	2% suc.	0.55% ag	30 g/L potato homogenate, 2 g/L Pt, 0.5 g/L ac	–	16 h/ 8 h	2, 5, 8 wks	Plantlet regeneration	Shin et al. 2008
Microlaelia lundii	Blue	SM	3% suc.	0.8% ag	1 g/L ac	25±2 °C	16 h/ 8 h	200 days	Plantlet regeneration	Favetta et al. 2017
**Rose**										

Rose	Blue	MS	3% suc.	0.25% gr	0.5 mg/L BAP + 0.1 g/L MI + 0.1g/L Ferrum	26±1 °C	16 h/ 8 h	2, and 4 wks	Plantlet regeneration	Azmi et al. 2015
Rosa × kordesii	80% red + 20% blue	MS	3% suc.	0.25% gr	1.5 g/L BAP + 0.1 mg/L IBA + 0.1 g/L MI + 0.1g/L Ferrum	26±1 °C	16 h/ 8 h	30 days	Plantlet regeneration	Azmi et al. 2014
**Chrysanthemum**										
Chrysanthemum× grandiflorum	8% blue + 2% green + 65% red + 25% far-red	MS	3% suc.	0.8% gr	–	23±0.5 °C	16 h/ 8 h	10 wks	Plantlet regeneration	Miler et al. 2019
C. morifolium cv. Ramat. Ellen	78% red + 14% blue + 8% far-red	MS	–	–	0.2 mg/L IAA + 0.05 mg/L BAP + ½ NH_4NO_3 + ½ KNO_3	–	–	42 days	Plantlet regeneration	Kurilčik et al. 2008
Dendranthema grandiflorum cv. Kitam 'Cheonsu'	Red, and far-red (plant growth), red + blue (stomata size)	MS	3% suc.	0.24% gr	–	25 °C (day) and 18 °C (night)	16 h/ 8 h	5 wks	Plantlet regeneration	Kim et al. 2004
**Weeping fig**										
Ficus benjamina	WF	MS	3% suc.	0.6% gr	4 mg/L BA + 30 mg/L addenine sulfate	23±0.5 °C	16 h/ 8 h	12 wks	Plantlet regeneration	Miler et al. 2019
**Gerbera**										
Gerbera jamesonii	70% red + 30% blue with 80 µmol m^{-2} s^{-1} photosynthetic photon flux density	MS	3% suc.	0.5% bag	5 µM BA + 0.5 µM NAA	23/21±1 °C	16 h/ 8 h	6 wks	Shoot multiplication	Cioć et al. 2019
G. jamesonii	12% blue + 19% green + 61% red + 8% far-red; 8% blue + 2% green + 65% red + 25% far-red; 1% UV + 20% blue + 39% green + 35% red + 5% far-red;	MS	3% suc.	0.8% gr	3 mg/L KIN	23±0.5 °C	16 h/ 8 h	8 wks	Plantlet regeneration	Miler et al. 2019
G. jamesonii	Red (shoot), blue (leaf size)	MS	3% suc.	0.5% bag	1 µM IAA	25 °C (day) and 23 °C (night)	16 h/ 8 h	6 wks	Acclimatization efficiency	Pawłowska et al. 2018a
G. jamesonii cv. "Dura"	50% red + 50% blue (SM); 70% red + 30% blue (SM, PPC, abc); 100% red (SE); 100% blue (LDW)	MS	3% suc.	0.5% bag	5 µM BA + 0.5 µM NAA	25 °C (day) and 23 °C (night)	16 h/ 8 h	6 wks	Shoot multiplication (SM); shoot elongation (SE); Photosynthetic pigment content (PPC); chlorophyll a and b and carotenoids (abc); Leaf dry weight (LDW)	Pawłowska et al. 2018b
**Coral bells**										
Heuchera× hybrida	8% blue + 2% green + 65% red + 25% far-red	MS	3% suc.	0.8% gr	0.1 mg/L BA + 0.1 mg/L IAA	23±0.5 °C	16 h/ 8 h	13 wks	Plantlet regeneration	Miler et al. 2019
**Bleeding heart**										
Lamprocapnos spectabilis	WF	MS	3% suc.	0.8% gr	0.25 mg/L BA + 0.25 mg/L IAA	23±0.5 °C	16 h/ 8 h	9 wks	Plantlet regeneration	Miler et al. 2019
**Anthurium**										
Anthurium andreanum	Blue or white or blue + red	MS	–	0.25% pg	PGRs supplemented (not specified)	24±2 °C	16 h/ 8 h	60 days	Plantlet regeneration	Bello-Bello et al. 2017
A. andreanum	Blue + red	MS	–	–	2 mg/L BAP	–	–	61 days	Plantlet regenaration	Martínez-Estrada et al. 2016
A. andreanum Lind.	Blue, blue + red	MS	3% suc.	0.6% ag	–	25 °C	12 h/ 12 h	60 days	Plantlet regeneration	Gu et al. 2012
	Red + blue	MS	3% suc.	0.6% ag	–	25 °C	12 h/ 12 h	60 days	Rooting	

Anthurium (Violeta and Pink Lady)	100% red (callus); 25% red + 75% blue or 100% blue (torpedo callus)	NN	–	–	1 mg/L 2,4-D + 1 mg/L KIN + 1 mg/L BA	24 °C (day) and 18 °C (night)	16 h/ 8 h	30-50 days	Callus	Budiarto 2010
	100% red or 75% red + 25% blue (SI); 25% red + 75% blue or 100% blue (SM)	NN	–	–	1 mg/L 2,4-D + 1 mg/L KIN + 1 mg/L BA	24 °C (day) and 18 °C (night)	16 h/ 8 h	60 days	Shoot induction and multiplication	
**Heliconia**										
Heliconia champneiana cv. Splash	100% red or (70% red + 30% blue	MS	3% suc.	0.18 gg	3 mg/L BAP	25±2 °C	16 h/ 8 h	6, 12 wks	Plantlet regeneration	Rodrigues et al. 2018
**Paeony**										
Paeonia suffruticosa	80% red + 20% blue	WPM	3% suc.	0.21 gg	4 mg/L IBA	25±1 °C	16 h/ 8 h	45 days	Plantlet regeneration	Wang et al. 2012b
**Spurflowers**										
Plectranthus scutellarioides	80% red + 20% green	MS	3% suc.	0.8% ag	0.5 g/L 2-(N-morpholino) ethane sulfonic acid	25±1.5 °C	16 h/ 8 h	28 days	Plantlet regeneration	Cho et al. 2019
**Crown of thorns**										
Euphorbia millii	Blue	MS	3% suc.	0.8% ag	1 mg/L BA + 0.3 mg/L IAA	25 °C	16 h/ 8 h	8 wks	Plantlet regeneration	Dewir et al. 2005
**Lilies**										
Spathiphyllum cannifolium	red, red + far-red (1:1)	MS	3% suc.	0.2% gr	1 mg/L BA + 0.3 mg/L IAA	25 °C	16 h/ 8 h	2 wks	Shoot proliferation	Dewir et al. 2005
Lilium hybrid "Pesaro"	Red + blue	MS	3% suc.	0.24% gr	1 mg/L BA + 0.3 mg/L NAA	–	–	60 days	Bulblets generation	Lian et al. 2012
**Giant protea**										
Protea cynaroides	red	MS	3% suc.	0.9% ag	–	21±2 (day) and 12±2 °C (night)	16 h/ 8 h	45 days	Shoot and root regeneration	Wu and Lin 2012
**Red ginger**										
Alpinia purpurata "Red Ginger" and "Pink"	white (other LED were not applied)	MS	3% suc.	0.6% ag	1.5 mg/L BAP + 0.1 mg/L NAA + 100 mg/L MI	25±2 °C	16 h/ 8 h	55 days	Plantlet regeneration	Pinheiro et al. 2019
**Tsuru-rindo**										
Tripterospermum japonicum	red (root), 70% red + 30% blue (plant growth)	MS 3%	suc.	0.3% gr –	24±1 °C 16 h/ 8 h 2, and 4 wks Plantlet				regeneration Moon et al. 2006	
**Hosta**										
Hosta plantaginea "Joseon"	red	MS	3% suc.	0.8% ag	0.1 mg/L MI	23±1 °C	16 h/ 8 h	6wks	Plantlet regeneration	Ku et al. 2016
**Poplar**										
Populus euramericana "Dorskamp"	50% red + 50% blue	1/2 MS	3% suc.	0.3% gr	4.4 μM BA + 2.2 μM ZT	24±1 °C	16 h/ 8 h	4 wks	Shoot regeneration	Kwon et al. 2015

Table S5. Studies in combination of light emitting diodes (LEDs) and plant growth regulators in vitro culture in ornamentals.

Plant species	Effective LEDs	Media	Carbon source	Solidifying agent	Effective PGRs	Temperature	Photoperiod (light/dark)	Culture period	Organogenesis/ regeneration	Ref

**Cymbidium orchid**										
Cymbidium dayanum	Green	mMS[1]	2% suc.	0.2% pg	0.1 mg/L chondroitin sulfate	25±1 °C	16 h/ 8 h	5-7 wks	PLB regeneration	Nahar et al. 2016
C. finlaysonianum	Green	mMS[1]	2% suc.	0.2% pg	1 mg/L chondroitin sulfate	25±1 °C	17 h/ 8 h	5-7 wks	PLB regeneration	
C. insigne	Green	mMS[1]	2% suc.	0.2% pg	1 mg/L CS	25±1 °C	16 h/ 8 h	8 wks	PLB regeneration	Nahar et al. 2013
C. dayanum	Blue	mMS[1]	2% suc.	0.2% pg	1 mg/L HA9	–	16 h/ 8 h	5 wks	PLBs proliferation	Nahar et al. 2017
	red or green	mMS [1]	2 % suc.	0.2 % pg	1 mg/L Ch	–	17 h/ 8 h	6 wks	PLBs proliferation	
**Dendrobium orchid**										
Dendrobium okinawense	green	mMS[1]	2% suc.	0.22% pg	0.01 mg/L PCIB	25±1 °C	16 h/ 8 h	42 days	PLB proliferation	Mehbub et al. 2022
D kingianum	White (other LEDs were not used)	mMS [1]	2 % suc.	0.22 % pg	**Expt BA** : 0.1 g/L; **Expt HA9**: 0.1 mg/L	25±1 °C	16 h/ 8 h	5 wks	PLB regeneration	Habiba et al. 2014
**Gerbera**										
Gerbera jamesonii cv. Queen Rebecca	red, green	MS	–	–	5 mg/L KN	20-23 °C	16h /8h	8 wks	Shoot multiplication	Gabryszewska and Rudnicki 1995
	red	MS	–	–	5 mg/L IAA	20 -24 °C	16 h /8h	9 wks	Rooting	
**Myrtle**										
Myrtus communis	red	MS	3% suc.	0.5% bio ag	1 or 5 μM BA	23/21±1 °C (day/night)	16h /8h	6 wks	Plantlet regeneration	Cioć et al. 2018
	100 % blue or 70% red + 30% blue	MS	3 % suc.	0.5 % bio ag	5 μM BA		16 h /8h	6 wks	Fresh weight	
**Bells of Ireland/ Shellflower**										
Moluccella laevis L.	PAR LED	MS	3% suc.	0.6% ag	3 μM BA + 0.5 μM NAA	25±0.5 °C	16h /8h	3 wks	Shoot multiplication	Zielińska et al. 2020

Supplemental references

1. Abu-Qaoud, H. Direct regeneration in *Cyclamen persicum* Mill. using seedling tissues. *An-Najah Univ. J. Res.-A* **2004**, *18*, 147–156.
2. Acemi, A. Chitosan versus plant growth regulators: a comparative analysis of their effects on in vitro development of *Serapias vomeracea* (Burm.f.) Briq. *Plant Cell Tiss. Organ Cult.* **2020**, *141*, 327–338.
3. Acemi, A.; Bayrak, B.; Çakır, M.; Demiryürek, E.; Gün, E.; Gueddari, N.E.E.; Özen, F. Comparative analysis of the effects of chitosan and common plant growth regulators on *in vitro* propagation of *Ipomoea purpurea* (L.) Roth from nodal explants. *In Vitro Cell. Dev. Biol.–Plant* **2018**, *54*, 537–544.
4. Afrin, S.; Rahman, M.A.; Khalekuzzaman, M.; Hasan, M.M.; Fahim, A.H.F.; Alam, M.A. Study on in vitro micropropagation of Rosa sp. *Bangladesh J. Agri.* **2022**, *47*, 66–74.
5. Ali, M.; Baloch, S.K.; Seema, N.; Yaeen, S.; Kaleri, A.A.; Kaleri, R.R.; Nizamani, G.S.; Subhapoto, G.F.; Kaleri, M.A.; Shahani, F.; Shahani, M. Influence of phytohormones on callus indication and micrpropagation on rose (*Rosa indica* L.). Journal of Basic & Applied Sciences **2018**, *14*, 9–11.
6. Ali, M.R.; Mehraj, H; Uddin, A.F.M.J. Kinetin (KIN) and indole-3-acetic acid (IAA) on *in vitro* shoot and root initiation of tuberose. *Int. J. Sust. Agril. Technol.* **2014**, *10*, 1–4.
7. Ali, M.R; Akand, M; Homayra, H; Mehraj, H; Uddin, A.F.M.J. *In vitro* regeneration and rapid multiplication of tuberose. *Int. J. Bus. Soc. Sci. Res.* **2015**, *3*, 35–38.
8. Alsemaan, T. Micro-propagation of Damask rose (*Rosa damascena* Mill.) cv. Almarah. *Int. J. Agric. Res.* **2013**, *8*, 172–177.
9. Amer, E.M.; Fetouh, M.I.; Rasha, S.E. Micropropagation and acclimatization of *Gardenia jasminoides* Ellis. *J. Biol. Chem. Environ. Sci.* **2019**, *14*, 107–120.
10. Asghar, S.; Ahmad, T.; Hafiz, I. A.; Yaseen, M. *In vitro* propagation of orchid (*Dendrobium nobile*) var. Emma white. *Afr. J. Biotechnol.*, **2011**, *10*, 3097–3103.
11. Aslam, J.; Mujib, A.; Sharma, M.P. *In vitro* micropropagation of *Dracaena sanderiana* Sander ex Mast: An important indoor ornamental plant. *Saudi J. Biol. Sci.* **2013**, *20*, 63–68.
12. Aung, W.T.; Bang, K.S.; Yoon, S.A.; Ko, B.; Bae, J.H. Effects of different natural extracts and plant growth regulators on plant regeneration and callus induction from pseudobulbs explants through in vitro seed germination of endangered orchid *Bulbophyllum auricomum* Lindl. J. Bio-Environ. Con. **2022**, *31*, 133–141.
13. Azmi, N.S.; Ahmad, R.; Ibrahim, R. Effects of red and blue (RB) LED on the *in vitro* growth of *Rosa kordesii* in multiplication phase. In 2nd International Conference on Agriculture and Biotechnology, IACSIT Press, Singapore, Volume 79, 2014.
14. Azmi, N.S.; Ahmad, R.; Ibrahim, R. Fluorescent light (FL), red led and blue led spectrums effects on in vitro shoots multiplication. *J. Teknol.* **2016**, *78*, 6.
15. Baker, A.; Kaviani, B.; Nematzadeh, G.; Negahdar, N. Micropropagation of *Orchis catasetum*–A rare and endangered orchid. *Acta Sci. Pol.–Hortorum Cultus* **2014**, *13*, 197–205.
16. Bakheet, G.I.; Soliman, S.S.; Abdelkader, M.A.I.; Elashtokhy, M.M.A. Effects of different croton (*Codiaeum variegatum* L.) genotypes and growth regulators on callus induction, micro propagation and antibacterial activities. *Zagazig J. Agric. Res.* **2018**, *45*, 331–347.
17. Balilashaki, K.; Vahedi, M.; Karimi, R. *In vitro* direct regeneration from node and leaf explants of *Phalaenopsis* cv. 'Surabaya'. *Plant Tissue Cult. Biotech.* **2015**, *25*, 193–205.
18. Baque, A.M.; Shin, Y.K.; Elshmari, T.; Lee, E.J.; Paek, K.Y. Effect of light quality, sucrose and coconut water concentration on the microporpagation of *Calanthe* hybrids ('Bukduseong' 'Hyesung' and 'Chunkwang' 'Hyesung'). *Aust. J. Crop Sci.* **2011**, *5*, 1247–1254.
19. Bello-Bello, J.J.; Perez-Sato, J.A.; Cruz-Cruz, C.A.; Martinez-Estrada, E. Light-emitting diodes: progress in plant micropropagation. In *Chlorophyll*, Jacob-Lopes, E., Zepka, L.Q., Queiroz, M.I., Eds.; IntechOpen: London, UK, Chapter 6, 2017; pp 93–103.
20. Belokurova, V.; Lystvan, K.; Volga, D.; Vasylenko, M.; Kuchuk, M. In vitro culture and some biochemical characteristics of *Fittonia albivenis* (Lindl. ex Veitch) Brummitt. *Agrbiodiv. Impr. Nut. Health Life Qual.* **2019**, *3*, 186–194.
21. Bhatt, A.; Stanly, C.; Keng, C.L. *In vitro* propagation of five *Alocasia species*. *Hortic. Bras.* **2013**, *31*, 210-215.
22. Bhattacharjee, B.; Islam, S.S. Effects of plant growth regulators on multiple shoot induction in *Vanda tessellata* (Roxb.) Hook. Ex G. Don an endangered medicinal orchid. *Int. J. Sci. Nat.* **2014**, *5*, 707–712.
23. Bhattacharyya, P.; Kumaria, S.; Job, N.; Tandon, P. Phyto-molecular profiling and assessment of antioxidant activity within micropropagated plants of *Dendrobium thyrsiflorum*: a threatened, medicinal orchid. *Plant Cell Tiss. Organ Cult.* **2015**, *122*, 535–550.
24. Bhattacharyya, P.; Kumaria, S.; Tandon, P. High frequency regeneration protocol for *Dendrobium nobile*: a model tissue culture approach for propagation of medicinally important orchid species. *S. Afr. J. Bot.* **2016**, *104*, 232–243.
25. Bhowmik, T.K.; Rahman, M.M. Micropropagation of commercially important orchid *Dendrobium palpebrae* Lindl. through *in vitro* developed pseudobulb culture. *J. Adv. Biotechnol. Exp. Ther.* **2020**, *3*, 225–232.
26. Billore, V.; Jain, M.; Suprasanna, P. Monochromic radiation through light-emitting diode (LED) positively augments *in vitro* shoot regeneration in Orchid (*Dendrobium sonia*). *Can. J. Biotech.* **2017**, *1*, 50.

27. Billore, V.; Mirajkar, S.J.; Suprasanna, P.; Jain, M. Gamma irradiation induced effects on in vitro shoot cultures and influence of monochromatic light regimes on irradiated shoot cultures of *Dendrobium sonia* orchid. *Biotechnol. Rep.* **2019**, *22*, e00343.
28. Budiarto, K. Spectral quality affects morphogenesis on anthurium plantlet during in vitro culture. *AGRIVITA J. Agric. Sci.* **2010**, *32*, 234–240.
29. Bustam, B.M.; Dixon, K.; Bunn, E. *Ex situ* germplasm preservation and plant regeneration of a threatened terrestrial orchid, *Caladenia huegelii*, through micropropagation and cryopreservation. *Aust. J. Bot.* **2016**, *64*, 659–663.
30. Cardoso, J.C. Silver nitrate enhances *in vitro* development and quality of shoots of *Anthurium andraeanum*. *Sci. Hortic.* **2019**, *253*, 358–363.
31. Cardoso, J.C.; Habermann, G. Adventitious shoot induction from leaf segments in *Anthurium andreanum* is affected by age of explant, leaf orientation and plant growth regulator. *Hortic. Environ. Biotechnol.* **2014**, *55*, 56–62.
32. Chauhan, S.; Promila Pathak, A.; Sharma, S.K. Teepol Regeneration of *Eulophia dabia* through rhizome explants and flowering: a study *in vitro*. *J. Orchid Soc. India* **2015**, *29*, 61–65.
33. Chavan, J. J.; Gaikwad, N. B.; Yadav, S. R. High multiplication frequency and genetic stability analysis of *Ceropegia panchganiensis*, a threatened ornamental plant of Western Ghats: conservation implications. *Sci. Hortic.* **2013**, *161*, 134-142.
34. Chavan, J.J.; Gaikwad, N.B.; Kshirsagar, P.R.; Umdale, S.D.; Bhat, K.V.; Dixit, G.B.; Yadav, S.R. Highly efficient *in vitro* proliferation and genetic stability analysis of micropropagated *Ceropegia evansii* by RAPD and ISSR markers: a critically endangered plant of Western Ghats. *Plant Biosyst.* **2015**, *149*, 442-450.
35. Chavan, J.J.; Nalawade, A.S.; Gaikwad, N.B.; Gurav, R.V.; Dixit, G.B.; Yadav, S.R. An efficient in vitro regeneration of *Ceropegia noorjahaniae*: an endemic and critically endangered medicinal herb of the Western Ghats. *Physiol. Mol. Biol. Plants* **2014**, *20*, 405–410.
36. Chavan, J.J.; Nimbalkar, M.S.; Adsul, A.A.; Kamble, S.S.; Gaikwad, N.B.; Dixit, G.B.; Gurav, R.V.; Bapat, V.A.; Yadav, S.R.Micropropagation and in vitro flowering of endemic and endangered plant *Ceropegia attenuata* Hook. *J. Plant Biochem. Biotechnol.* **2011**, *20*, 276–282.
37. Chen, B.; Trueman, S. J.; Li, J.; Li, Q.; Fan, H.; Zhang, J. Micropropagation of the endangered medicinal orchid, *Dendrobium officinale*. *Life Sci. J.* **2014**, *11*, 526–530.
38. Chen, J.T.; Chang, W.C. Direct somatic embryogenesis and plant regeneration from leaf explants of *Phalaenopsis amabilis*. *Biol. Plant.* **2006**, *50*, 169–173.
39. Chen, J.T.; Chang, WC. TIBA affects the induction of direct somatic embryogenesis from leaf explants of *Oncidium*. *Plant Cell Tiss. Organ Cult*. **2004**, *79*, 315–320.
40. Chhalgri, M.A.; Khan, M.T.; Nizamani, G.S.; Yasmeen, S.; Khan, I.A.; Aslam, M.M.; Rajpar A.A.; Tayyaba, Nizamani F.; Nizamani M.R.; Iqbal R.; Panhwar M.J.; Siddiqui, M.A. Effect of plant growth hormones on shoot and root regeneration in rose under *in vitro* conditions. *Adv. Life Sci.* **2020**, *8*, 93–97.
41. Cho, K.H.; Laux, V.Y.; Wallace-Springer, N.; Clark, D.G.; Folta, K.M.; Colquhoun, T.A. Effects of light quality on vegetative cutting and in vitro propagation of coleus (*Plectranthus scutellarioides*). *HortSci.* **2019**, *54*, 926–935.
42. Choi, H.; Yang, J.C.; Ryu, S.H.; Yoon, S.M.; Kim, S.Y.; Lee, S.Y. *In vitro* multiplication of *Hosta* Tratt. species native to Korea by shoot-tip culture. *Korean J. Plant Resour.* **2019**, *32*, 53–62.
43. Chookoh, N.; Chiu, Y.T.; Chang, C.; Hu, W.H.; Dai, T.E. Micropropagation of *Tolumnia* orchids through induction of protocorm-like bodies from leaf segments. *HortSci.* **2019**, *54*, 1230–1236.
44. Cioć, M.; Kalisz, A.; Żupnik, M.; Pawłowska, B. Different LED light intensities and 6-benzyladenine concentrations in relation to shoot development, leaf architecture, and photosynthetic pigments of *Gerbera jamesonii* Bolus in vitro. *Agronomy* **2019**, *9*, 358.
45. Cioć, M.; Szewczyk, A.; Żupnik, M.; Kalisz, A.; Pawłowska, B. LED lighting affects plant growth, morphogenesis and phytochemical contents of *Myrtus communis* L. in vitro. *Plant Cell Tiss. Organ Cult.* **2018**, *132*, 433–447.
46. Coello, C.Y.; Miceli, C.L.; Orantes, C., Dendooven, L.; Gutiérrez, F.A. Plant growth regulators optimization for *in vitro* cultivation of the orchid *Guarianthe skinneri* (Bateman) Dressier & WE Higgins. *Gayana Bot.* **2010**, *67*, 19–26.
47. Cybularz-Urban, T.; Hanus-Fajerska, E.; Swiderski, A. Effect of light wavelength on in vitro organogenesis of a *Cattleya* hybrid. *Acta Biol. Crac. Ser. Bot.* **2007**, *49*, 113–118.
48. da Silva, J.A.T. Response of hybrid *Cymbidium* (Orchidaceae) protocorm-like bodies to 26 plant growth regulators. *Bot. Lith.* **2014a**, *20*, 3–13. da Silva, J.A.T. The effect of ethylene inhibitors (AgNO3, AVG), an ethylene-liberating compound (CEPA) and aeration on the formation of protocorm-like bodies of hybrid *Cymbidium* (Orchidaceae). *Front. Biol.* **2013**, *8*, 606–610.
49. da Silva, J.A.T. The response of protocorm-like bodies of nine hybrid *Cymbidium* cultivars to light-emitting diodes. *Environ. Exp. Biol.* **2014b**, *12*, 155–159.
50. Daneshvar, M.H; Havil, M; Lotfi Jalal-Abadi, A. Micropropagation of *Polianthes tuberosa* L. through direct organogenesis. *J. Plant Prod.* **2022**, *45*,
51. Decruse, S.W.; Gangaprasad, A.; Seeni, S.; Menon, V.S. A protocol for shoot multiplication from foliar meristem of *Vanda spathulata* (L.) Spreng. *Indian J. Exp. Biol.* **2003**, *41*, 924–927.

52. Deshmukh, V.D.; Kharde, A.V.; Talekar, B.K. Interactive effects of BA and IAA on shoot proliferation of gladiolus (*Gladiolus grandiflorus*) var. White Prosperity. *J. Oriental Res. Madras* **2021**, *XC*, II-VII.
53. Devi, P.; Kumar, P.; Sengar, R.S.; Yadav, M.K.; Kumar, M.; Singh, S.K.; Singh, S. *In-vitro* multiple shoots production from cormel shoot buds in gladiolus (*Gladiolus hybrida*). *Int. J. Curr. Microbiol. App. Sci.* **2019**, *8*, 1345–1350.
54. Dewir, Y.H.; Chakrabarty, D.; Kim, S.J.; Hahn, E.J.; Paek, K.Y. Effect of light-emitting diode on growth and shoot proliferation of *Euphorbia millii* and *Spathiphyllum cannifolium*. *J. Kor. Soc. Hort. Sci.* **2005**, *46*, 375–379.
55. Dhir, R.; Shekhawat, G.S. Ecorehabilitation and biochemical studies of *Ceropegia bulbosa* Roxb.: a threatened medicinal succulent. *Acta Physiol. Plant.* **2014**, *36*, 1335–1343.
56. Dhir, R.; Shekhawat, G.S. Production, storability and morphogenic response of alginate encapsulated axillary meristems and genetic fidelity evaluation of *in vitro* regenerated *Ceropegia bulbosa*: a pharmaceutically important threatened plant species. *Ind. Crops Prod.* **2013**, *47*, 139–144.
57. Doğan, S.; Çağlar, G.; Palaz, E. B. The effect of different applications on *in vitro* bulb development of an endemic hyacinth plant (*Hyacinthus orientalis* L. subsp. *chionophyllus* Wendelbo) grown in Turkey. *Turkish JAF Sci. Technol.* **2020**, *8*, 1713–1719.
58. Driver, J.A.; A.H. Kuniyuki. *In vitro* propagation of Paradox walnut rootstock. *HortSci.* **1984**, *19*, 507–509.
59. Duhoky, M.M.; Al-Mizory, L.S. *In vitro* micropropagation of selected *Bougainvillea* sp. through callus induction. *J. Agric. Vet. Sci.* **2014**, *6*, 1–6.
60. Faisal, M.; Ahmad, N.; Anis, M. In vitro regeneration and mass propagation of *Ruta graveolens* L.—a multipurpose shrub. *HortSci.* **2005**, *40*, 1478–1480.
61. Faisal, M.; Ahmad, N.; Anis, M.; Alatar, A.A.; Qahtan, A.A. Auxin-cytokinin synergism *in vitro* for producing genetically stable plants of *Ruta graveolens* using shoot tip meristems. *Saudi J. Biol. Sci.* **2018**, *25*, 273–277.
62. Farooq, I.; Qadri, Z.A.; Rather, Z.A.; Nazki, I.T.; Banday, N.; Rafiq, S.; Mansoor, S. Optimization of an improved, efficient and rapid *in vitro* micropropagation protocol for *Petunia hybrida* Vilm. Cv. "Bravo". *Saudi J. Biol. Sci.* **2021**, *28*, 3701–3709.
63. Favetta, V.; Colombo, R.C.; Mangili Júnior, J.F.; de Faria, R.T. Light sources and culture media in the *in vitro* growth of the Brazilian orchid *Microlaelia lundii*. *Semina: Ciênc. Agrár.* **2017**, *38*, 1775–1784.
64. Gabryszewska, E.; Rudnicki, R. The influence of light quality on the shoot proliferation and rooting of Gerbera jamesonii *in vitro*. *Acta Agrobot.* **1995**, *48*, 105–111.
65. Gajbhiye, S.S; Tripathi, M.K; Vidya, M.S; Singh, M.; Baghel, B.S.; Tiwari, S. Direct shoot organogenesis from cultured stem disc explants of tuberose (*Polianthes tuberosa* Linn.). *J. Agric. Technol.* **2011**, *7*, 695–709.
66. Gantait, S., Sinniah, U.R. Rapid micropropagation of monopodial orchid hybrid (*Aranda* Wan Chark Kuan 'Blue' × *Vanda coerulea* Grifft. ex. Lindl.) through direct induction of protocorm-like bodies from leaf segments. *Plant Growth Regul.* **2012**, *68*, 129–140.
67. Ghareeb, Z.F.; Taha, L.S. Micropropagation protocol for *Antigonon leptopus* an important ornamental and medicinal plant. *J. Genet. Eng. Biotechnol.* **2018**, *16*, 669–675.
68. Godo, T.; Fujiwara, K.; Guan, K.; Miyoshi, K. Effects of wavelength of LED-light on *in vitro* asymbiotic germination and seedling growth of *Bletilla ochracea* Schltr.(Orchidaceae). Plant Biotechnol. **2011**, *28*, 398–400.
69. Goswami, K.; Yasmin, S.; Nasiruddin, K. M.; Khatun, F.; Akte, J. *In vitro* regeneration of *Dendrobium* sp. of orchid using leaf tip as explant. *J. Environ. Sci. Nat. Resour.* **2015**, *8*, 75–78.
70. Govindaraju, S.; Arulselvi, P. I. Effect of cytokinin combined elicitors (l-phenylalanine, salicylic acid and chitosan) on *in vitro* propagation, secondary metabolites and molecular characterization of medicinal herb–*Coleus aromaticus* Benth (L). *J. Saudi Soc. Agric. Sci.* **2018**, *17*, 435–444.
71. Gow, W.P.; Chen, J.T.; Chang, W.C. Effects of genotype, light regime, explant position and orientation on direct somatic embryogenesis from leaf explants of *Phalaenopsis* orchids. *Acta Physiol. Plant.* **2009**, *31*, 363–369.
72. Gow, W.P.; Chen, J.T.; Chang, W.C. Enhancement of direct somatic embryogenesis and plantlet growth from leaf explants of *Phalaenopsis* by adjusting culture period and explant length. *Acta Physiol. Plant.* **2010**, *32*, 621–627.
73. Gu, A.; Liu, W.; Ma, C.; Cui, J.; Henny, R.J.; Chen, J. Regeneration of *Anthurium andraeanum* from leaf explants and evaluation of microcutting rooting and growth under different light qualities. *HortSci.* **2012**, *47*, 88–92.
74. Guo, W.L.; Chang, Y.C.A.; Kao, C.Y. Protocorm-like bodies initiation from root tips of *Cyrtopodium paranaense* (Orchidaceae). *HortSci.* **2010**, *45*, 1365–1368.
75. Habas, R.R.; Turker, M.; Ozdemir, F.A. *In vitro* multiple shoot regeneration from *Petunia hybrida*. *Turkish JAF Sci. Technol.* **2019**, *7*, 1554–1560.
76. Habiba, S.U.; Shimasaki, K.; Ahasan, M.M.; Alam, M.M. Effect of 6-benzylaminopurine (BA) and hyaluronic acid (HA) under white light emitting diode (LED) on organogenesis in protocorm-likebodies (PLBs) of *Dendrobium kingianum*. *Am.–Eurasian J. Agric. Environ. Sci.* **2014**, *14*, 605–609.
77. Habiba, S.U.; Shimasaki, K.; Ahasan, M.M.; Alam, M.M. Effects of different light quality on growth and development of protocorm-like bodies (PLBs) in *Dendrobium kingianum* cultured in vitro. *Bangladesh Res. Pub. J.* **2014**, *10*, 223–227.
78. Hajong, S.; Kumaria, S.; Tandon, P. Effect of plant growth regulators on regeneration potential of axenic nodal segments of *Dendrobium chrysanthum* Wall. ex Lindl. *J. Agr. Sci. Tech.* **2013**, *15*, 1425–1435.

79. Han, B.H.; Yae, B.W.; Yu, H.J.; Peak, K.Y. Improvement of in vitro micropropagation of *Lilium* oriental hybrid 'Casablanca'by the formation of shoots with abnormally swollen basal plates. *Sci. Hortic.* **2005**, *103*, 351–359.
80. Haque, S.M.; Ghosh, B. *In vitro* completion of sexual life cycle: production of R1 plants of *Ipomoea quamoclit* L. *Propag. Ornam. Plants* **2013**, *13*, 19–24.
81. Hernández-Mendoza, F.; Carrillo-Castañeda, G.; García-Gaytán, V.; Pedraza-Santos, M.; de la Cruz-Torres, E.; MendozaCastillo, M. *In vitro* plant regeneration of *Polianthes tuberosa* L. from leaf and flower buds tissue. *Trop. Subtrop. Agroecosystems* **2021**, *24*, 55.
82. Hoda, E. *In vitro* regeneration and somaclonal variation of *Catharanthus roseus* Don. using leaf and internodal explants. *Alex. Sci. Exch. J.* **2013**, *34*, 452–459.
83. Hossen, M.M.; Saha, S.; Khatun, F. Effects of plant growth regulators on *in vitro* growth and development of orchid (*Dendrobium* sp.) from protocorm like bodies (PLBs). *J. Bangladesh Agric. Univ.* **2021**, *193*, 294–301.
84. Hung, C.Y.; Zhang, J.; Bhattacharya, C.; Li, H.; Kittur, F.S.; Oldham, C.E.; Wei, X.; Burkey, K.O,; Chen, J.; Xie, J. Transformation of long-lived albino *Epipremnum aureum* 'Golden Pothos' and restoring chloroplast development. *Front. Plant Sci.* **2021**, *12*, 647507.
85. Ibrahim, M.A., Draaj, I.A. The effect of explant source and cytokinin concentration on the direct bulb formation of tulip (*Tulipa gesnerina* L.) by plant tissue culture technique. *Plant Cell Biotechnol. Mol. Biol.* **2020**, *21*, 111–119.
86. Islam, S.S., Islam, T., Bhattacharjee, B., Mondal, T.K., Subramaniam, S. *In vitro* pseudobulb based micropropagation for mass development of *Cymbidium finlaysonianum* Lindl. *Emir. J. Food Agric.* **2015**, *27*, 469–474.
87. İzgü, T.; Sevindik, B.; Çürük, P.; Şimşek, Ö.; Kaçar, Y.A.; da Silva, J.A.T.; Mendi, Y. Development of an efficient regeneration protocol for four *Cyclamen* species endemic to Turkey. *Plant Cell Tiss. Organ Cult.* **2016**, *127*, 95–113.
a. Jabbarzadeh, Z; Khosh-Khui, M. Factors affecting tissue culture of Damask rose (*Rosa damascena* Mill.). *Sci. Hortic.* **2005**, *105*, 475–482.
88. Jainol, J.E.; Gansau, J.A. Effect of growth regulators and explant orientation on shoot tip culture of Borneo endemic orchid, *Dimorphorchis lowii*. *Trans. Sci. Technol.* **2016**, *3*, 306–312.
89. Jalali, N.; Naderi, R.; Shahi-Gharahlar, A.; da Silva, J.A.T. Tissue culture of *Cyclamen* spp. *Sci. Hortic.* **2012**, *137*, 11–19.
90. Javaheri, N.; Kaviani, B. Effect of hormonal combination of auxin and cytokinin on micropropagation of eastern lily (*Lilium* oriental hybrid 'Casablanca') plant using bulb scale explant. *J. Hort. Sci.* **2022**, *36*, 57–69.
91. Kaewjampa, N.; Shimasaki, K. Effects of green LED lighting on organogenesis and superoxide dismutase (SOD) activities in protocorm-like bodies (PLBs) of *Cymbidium* cultured *in vitro*. *Environ. Control Biol.* **2012**, *50*, 247–254.
92. Kaewjampa, N.; Shimasaki, K.; Nahar, S.J. Hyaluronic acid can be a new plant growth regulator for hybrid *Cymbidium* micropropagation. *Plant Tissue Cult. Biotech.* **2012**, *22*, 59–64.
93. Kalimuthu, K.; Senthilkumar, R.; Vijayakumar, S. *In vitro* micropropagation of orchid, *Oncidium* sp. (Dancing Dolls). *Afr. J. Biotechnol*. **2007**, *6*, 1171–1174.
94. Kalyan, K.D.; Sil, S. Protocorm-like bodies and plant regeneration from foliar explants of *Coelogyne flaccida*, a horticulturally and medicinally important endangered orchid of eastern himalaya. *Lankesteriana* **2015**, *15*, 151–158.
95. Kananont, N.; Pichyangkura, R.; Chanprame, S.; Chadchawan, S.; Limpanavech, P. Chitosan specificity for the *in vitro* seed germination of two *Dendrobium* orchids (Asparagales: Orchidaceae). *Sci. Hortic.* **2010**, *124*, 239–247.
96. Kanchanapoom, K.; Posayapisit, N.; Kanchanapoom, K. In vitro flowering from cultured nodal explants of rose (*Rosa hybrida* L.). *Not. Bot. Horti Agrobot. Cluj-Napoca* **2009**, *37*, 261–263.
97. Kanchanapoom, K.; Sakpeth, P.; Kanchanapoom, L. In vitro flowering of shoots regenerated from cultured nodal expiants of *Rosa hybrida* cv. 'Heirloom'. *ScienceAsia* **2010**, *36*, 161–164.
98. Kanwar, J.K.; Kumar, S. Influence of growth regulators and explants on shoot regeneration in carnation. *Hort. Sci.* **2009**, *36*, 140–146.
99. Kaur, S.; Bhutani, K.K. *In vitro* mass propagation of ornamentally and medicinally important *Coelogyne flaccida* Lindl. through pseudobulb segments. *Plant Tissue Cult. Biotech.* **2013**, *23*, 39–47.
100. Kazeroonian, R.; Mousavi, A.; Jari, S.K.; Tohidfar, M. Factors influencing *in vitro* organogenesis of *Chrysanthemum morifolium* cv. 'Resomee Splendid'. *Iranian J. Biotech.* **2018**, 16, e1454.
101. Khalafalla, M.M.; Elgaali, E.I.; Ahmed, M.M. *In vitro* multiple shoot regeneration from nodal explants of *Vernonia amygdalina*-an important medicinal plant. *Afr. Crop Sci. Confer. Proceed.* **2007**, *8*, 747–752.
102. Khanchana, K.; Kannan, M.; Hemaprabha, K.; Ganga, M. Standardization of protocol for sterilization and *in vitro* regeneration in tuberose (*Polianthes tuberosa* L.). *Int. J. Chem. Stu.* **2019**, *7*, 236–241.
103. Khaskheli, A.J.; Khaskheli, M.I.; Khaskheli, M.A.; Shar, T.; Ahmad, W.; Lighari, U.A.; Khaskheli, M.A.; Khaskheli, A.A.; Makan, F.H. Proliferation, multiplication and improvement of micro-propagation system for mass clonal production of rose through shoot tip culture. *Am. J. Plant Sci.* **2018**, *9*, 296–310.
104. Khatri, P.; Rana, J.S.; Sindhu, A.; Jamdagni, P. Effect of additives on enhanced in-vitro shoot multiplication and their functional group identification of *Chlorophytum borivilianum* Sant. Et Fernand. *SN Appl. Sci.* **2019**, *1*, 1105.
105. Khatun, H.; Khatun, M.; Biswas, M.; Kabir, M.; Al-Amin, M. *In vitro* growth and development of *Dendrobium* hybrid orchid. *Bangladesh J. Agric. Res.* **2010**, *35*, 507–514.

106. Khatun, K.; Nath, U.K.; Rahman, M.S. Tissue culture of *Phalaenopsis*: Present status and future prospects. *J. Adv. Biotechnol. Exp. Therap.* **2020**, *3*, 273–285.
107. Khatun, M., Khatun, H., Khanam, D., Al-Amin, M. *In vitro* root formation and plantlet development in Dendrobium orchid. *Bangladesh J. Agric. Res.* **2010**, *35*, 257–265.
108. Kher, M.M.; Nataraj, M.; Parmar, H.D.; Buchad, H. Micropropagation of *Merremia quinquefolia* (L.) Hallier F. from nodal explants. *J. Hortic. Res.* **2015**, *23*, 13–16.
109. Kim, S.J.; Hahn, E.J.; Heo, J.W.; Paek, K.Y. Effects of LEDs on net photosynthetic rate, growth and leaf stomata of chrysanthemum plantlets in vitro. *Sci. Hortic.* **2004**, *101*, 143–151.
110. Knudson, L. A new nutrient solution for the germination of orchid seed. *Amer. Orchid Soc. Bull.* **1946**, 15, 214–217.
111. Krishnareddy, P.V.; Pullaiah, T. *In vitro* conservation of *Ceropegia elegans*, an endemic plant of South India. *Afr. J. Biotechnol.* **2012**, *11*, 12443–12449.
112. Kritskaya, T.A.; Kashin, A.S.; Kasatkin, M.Y. Micropropagation and somaclonal variation of *Tulipa suaveolens* (Liliaceae) in vitro. *Russ. J. Dev. Biol.* **2019**, *50*, 209–215.
113. Ku, B. S.; Cho, M. S. *In vitro* multiplication of *Hosta plantaginea* 'Joseon' by shoot-tip culture. *Flower Res. J.* **2016**, *24*, 328–336.
114. Kulus, D.; Miler, N. Application of plant extracts in micropropagation and cryopreservation of bleeding heart: An ornamental-medicinal plant species. *Agriculture* **2021**, *11*, 542.
115. Kumar, A.; Kumar, A.; Sharma, V.; Mishra, A.; Singh, S.; Kumar, P. *In vitro* regeneration of gladiolus (*Gladiolus hybrida* L.): Optimization of growth media and assessment of genetic fidelity. *Int. J. Curr. Microbiol. App. Sci.* **2018**, *7*, 2900–2909.
116. Kumar, A; Sood, A; Palni, U; Gupta, A; Palni, L.M. Micropropagation of *Rosa damascena* Mill. from mature bushes using thidiazuron. *J. Hort. Sci. Biotechnol.* **2001**, *76*, 30–34.
117. Kumar, V.; Moyo, M.; Van Staden, J. Enhancing plant regeneration of *Lachenalia viridiflora*, a critically endangered ornamental geophyte with high floricultural potential. *Sci. Hortic.* **2016**, *211*, 263–268.
118. Kumaria, S.; Kehie, M., Bhowmik, S.S.D.; Singh, M.; Tandon, P. In vitro regeneration of *Begonia rubrovenia* var. *meisneri* CB Clarke—A rare and endemic ornamental plant of Meghalaya, India. *Indian J. Biotechnol.* **2012**, *11*, 300–303.
119. Kuo, H.L.; Chen, J.T.; Chang, W.C. Efficient plant regeneration through direct somatic embryogenesis from leaf explants of *Phalaenopsis* 'Little Steve'. *In Vitro Cell. Dev. Biol.–Plant* **2005**, *41*, 453–456.
120. Kurilčik, A.; Miklušytė-Čanova, R.; Dapkūnienė, S.; Žilinskaitė, S.; Kurilčik, G.; Tamulaitis, G.; Duchovskis, P.; Žukauskas, A. *In vitro* culture of *Chrysanthemum* plantlets using light-emitting diodes. *Cent. Eur. J. Biol.* **2008**, *3*, 161–167.
121. Kwon, A.R.; Cui, H.Y.; Lee, H.; Lee, H.; Shin, H.; Kang, K.S.; Park, S.Y. Light quality affects shoot regeneration, cell division, and wood formation in elite clones of *Populus euramericana*. *Acta Physiol. Plant.* **2015**, *37*, 65.
122. Le, V.T.; Tanaka, M. Effects of red and blue light-emitting diodes on callus induction, callus proliferation, and protocormlike body formation from callus in *Cymbidium* orchid. *Environ. Control Biol.* **2004**, *42*, 57–64.
123. Lian, M.L.; Murthy, H.N.; Paek, K.Y. Effects of light emitting diodes (LEDs) on the in vitro induction and growth of bulblets of *Lilium* oriental hybrid 'Pesaro'. *Sci. Hortic.* **2002**, *94*, 365–370.
124. Liberman, R.; Shahar, L.; Nissim-Levi, A.; Evenor, D.; Reuveni, M.; Oren-Shamir, M. Shoot regeneration from leaf explants of *Brunfelsia calycina*. *Plant Cell Tiss. Organ Cult.* **2010**, *100*, 345–348.
125. Lin, Y.; Li, J.; Li, B.; He, T.; Chun, Z. Effects of light quality on growth and development of protocorm-like bodies of *Dendrobium officinale* in vitro. *Plant Cell Tiss. Organ Cult.* **2011**, *105*, 329–335.
126. Longchar, T.B.; Deb, C.R. Optimization of *in vitro* propagation protocol of *Dendrobium heterocarpum* Wall. ex. Lindl. and clonal genetic fidelity assessment of the regenerates: An orchid of horticultural and medicinal importance. *S. Afr. J. Bot.* **2022**, *149*, 67–78.
127. Luan, V.Q., Huy, N.P., Nam, N.B., Huong, T.T., Hien, V.T., Hien, N.T.T., Hai, N.T.; Thinh, D.K.; Nhut, D.T. Ex vitro and in vitro *Paphiopedilum delenatii* Guillaumin stem elongation under light-emitting diodes and shoot regeneration via stem node culture. *Acta Physiol. Plant.* **2015**, *37*, 1-11.
128. Lukatkin, A.S.; Mokshin, E.V.; Bolshakova, E.V.; da Silva, J.A.T. Effects of inorganic salts concentration and alternative plant growth regulators on the *in vitro* organogenesis of a new hybrid *Cymbidium*. *BioTechnologia* **2019**, *100*, 279– 288.
129. Lukatkin, A.S.; Mokshin, E.V.; da Silva, J.A.T. Use of alternative plant growth regulators and carbon sources to manipulate *Dianthus caryophyllus* L. shoot induction in vitro. *Rend. Fis. Acc. Lincei* **2017**, *28*, 583–588.
130. Luo, J.P; Wang, Y.; Zha, X.Q.; Huang, L. Micropropagation of *Dendrobium densiflorum* Lindl. ex Wall. through protocormlike bodies: effects of plant growth regulators and lanthanoids. *Plant Cell Tiss. Organ Cult.* **2008**, *93*, 333.
131. Maheswari, N.U.; Vaishnavi, K. In vitro micropropagation of *Rosa damascena* Mill. *Asian J. Multidimen. Res.* **2018**, *7*, 107– 114.
132. Malabadi, R.B.; Mulgund, G.S.; Kallappa, N. Micropropagation of *Dendrobium nobile* from shoot tip sections. J. *Plant Physiol.* **2005**, *162*, 473–478.
133. Marconi, P.L.; Radice, S. Organogenesis and somatic embryogenesis in *Codiaeum variegatum* (L.) Blume cv. "Corazón de Oro". *In Vitro Cell. Dev. Biol.–Plant* **1997**, *33*, 258–262.

134. Martin, K.P.; Madassery, J. Rapid in vitro propagation of *Dendrobium* hybrids through direct shoot formation from foliar explants, and protocorm-like bodies. *Sci. Hortic.* **2006**, *108*, 95–99.
135. Martínez-Estrada, E.; Caamal-Velázquez, J.H.; Morales-Ramos, V.; Bello-Bello, J.J. Light emitting diodes improve *in vitro* shoot multiplication and growth of *Anthurium andreanum* Lind. *Propag. Ornam. Plants* **2016**, *16*, 3–8.
136. Maślanka, M.; Bach, A. Induction of bulb organogenesis in *in vitro* cultures of tarda tulip (*Tulipa tarda* Stapf.) from seedderived explants. *In Vitro Cell. Dev. Biol.–Plant* **2014**, *50*, 712–721.
137. Masnoddin, M.; Repin, R.; Abd Aziz, Z. Micropropagation of an endangered Borneo orchid, *Paphiopedilum rothschildianum* callus using temporary immersion bioreactor system. *Thai Agric. Res. J.* **2016**, *34*, 161–171.
138. Matand, K.; Shoemake, M.; Li, C. High frequency in vitro regeneration of adventitious shoots in daylilies (*Hemerocallis* sp) stem tissue using thidiazuron. *BMC Plant Biol.* **2020**, *20*, 31.
139. Mata-Rosas, M.; Baltazar-García, R.J.; Chávez-Avila, V.M. In vitro regeneration through direct organogenesis from protocorms of *Oncidium tigrinum* Llave & Lex. (Orchidaceae), an endemic and threatened Mexican species. *HortSci.* **2011**, *46*, 1132–1135.
140. Mateen, R.M. Development and optimization of micro-propagation, in vitro methodology for gladiolus. *BioSci. Rev.* **2019**, *1*, 21–36.
141. Matos, A.V.C.D.S.D., Oliveira, B.S.D., Oliveira, M.E.B.S.D., Cardoso, J.C. AgNO3 improved micropropagation and stimulate *in vitro* flowering of rose (*Rosa* x *hybrida*) cv. Sena. *Ornam. Hortic.* **2020**, *27*, 33–40.
142. Mayer, J.L.S.; Stancato, G.C.; Appezzato-Da-Glória, B. Direct regeneration of protocorm-like bodies (PLBs) from leaf apices of *Oncidium flexuosum* Sims (Orchidaceae). *Plant Cell Tiss. Organ Cult.* **2010**, *103*, 411–416.
143. Mehbub, H.; Shimasaki, K.; Mehraj, H. Low concentration of anti-auxin and anti-fungal agent accelerates the PLB regeneration of *Dendrobium okinawense* under green LED. *Plants* **2022**, *11*, 1082.
144. Mehraj, H.; Alam, M.M.; Habiba, S.U.; Mehbub, H. LEDs Combined with CHO Sources and CCC priming PLB regeneration of *Phalaenopsis*. *Horticulturae* **2019**, *5*, 34.
145. Mehraj, H.; Shimasaki, K. *In vitro* PLBs organogenesis of *Phalaenopsis* using different concentrations of HA9 and HA12 combination. *J. Biosci. Agric. Res.* **2017**, *12*, 1036–1040.
146. Mei-Yin, C.; Sani, H. *In vitro* plantlet regeneration from nodal explant and callus induction of *Vernonia amygdalina* Delile. *J. Plant Sci.* **2018**, *6*, 1–6.
147. Memon, N.; Qasim, M.; Jaskani, M.J.; Ahmad, R. *In vitro* cormel production of gladiolus. *Pak. J. Agri. Sci.* **2010**, *47*, 115–123.
148. Mengxi, L.; Zhigang, X.; Yang, Y.; Yijie, F. Effects of different spectral lights on *Oncidium* PLBs induction, proliferation, and plant regeneration. *Plant Cell Tiss. Organ Cult.* **2011**, *106*, 1–10.
149. Miler, N., Kulus, D., Woźny, A. Rymarz, D.; Hajzer, M.; Wierzbowski, K.; Nelke, R.; Szeffs, L. Application of widespectrum light-emitting diodes in micropropagation of popular ornamental plant species: a study on plant quality and cost reduction. *In Vitro Cell. Dev. Biol.–Plant* **2019**, *55*, 99–108.
150. Minerva, G.; Kumar, S. Micropropagation of gerbera (*Gerbera jamesonii* Bolus). In *Protocols for Micropropagation of Selected Economically-important Horticultural Plant*s, Lambardi, M., Ozudogru, E., Jain, S., Eds.; Humana Press: New Jersey, USA, Chapter 24, 2012, pp. 305–316.
151. Mirzaei, S; Zare, A.G; Jafary, S. Evaluating micropropagation of Kashan Damask rose, Yasooj aromatic rose and their hybrid. *Int. J. Env. Agric. Biotech.* **2019**, *4*, 1407–1413.
152. Mitra, G.C.; Prasad, R.N.; Choudhury, R.A. Inorganic salts & differentiation of protocorms in seed callus of an orchid & correlated changes in its free amino acid content. *Indian J Exp. Biol.* **1976**, *14*, 350–351
153. Moon, H.K.; Park, S.Y. Kim, Y.W.; Kim, C.S. Growth of Tsuru-rindo (*Tripterospermum japonicum*) cultured *in vitro* under various sources of light-emitting diode (LED) irradiation. *J. Plant Biol.* **2006**, *49*, 174–179.
154. Mose, W.; Daryono, B.S.; Indrianto, A.; Purwantoro, A.; Semiarti, E. Direct somatic embryogenesis and regeneration of an Indonesian orchid *Phalaenopsis amabilis* (L.) Blume under a variety of plant growth regulators, light regime, and organic substances. *Jordan J. Biol. Sci.* **2020**, *13*, 509–518.
155. Nahar, S.J.; Haque, S.M.; Kazuhiko, S. Application of chondroitin sulfate on organogenesis of two *Cymbidium* spp. under different sources of lights. *Not. Sci. Biol.* **2016**, *8*, 156–160.
156. Nahar, S.J.; Haque, S.M.; Shimasaki, K. Effect of light quality and plant growth regulator on organogenesis of orkid *Cymbidium dayanum*. *Bangladesh J. Agric. Res.* **2017**, *42*, 185–190.
157. Nahar, S.J.; Shimasaki, K. Application of 5-aminolevulinic acid for the *in vitro* micropropagation of *Cymbidium* as a potential novel plant regulator. *Environ. Control Biol.* **2014**, *52*, 117–121.
158. Nahar, S.J.; Shimasaki, K.; Haque, S.M. Chondroitin sulfate can be a new plant growth regulator for *Cymbidium* micropropagation. *Acta Hortic.* **2013**, *1014*, 449–455.
159. Nahar, S.J.; Shimasaki, K.; Huang, C.L.; Naruemol, K. Effect of plant growth regulators on organogenesis in protocormlike body (PLBs) of *Cymbidium dayanum* in vitro. *ARPN J. Agric. Biol. Sci.* **2011**, *6*, 28–33.
160. Nahar, S.J.; Syed, M.H.; Shimasaki, K. Organogenesis of *Cymbidium* orchid using elicitors. *J. Plant Develop.* **2015**, *22*, 13–20.

161. Naing, A.H.; Jeon, S.M.; Han, J.S.; Lim, S.H.; Lim, K.B.; Kim, C.K. Factors influencing *in vitro* shoot regeneration from leaf segments of *Chrysanthemum*. *C. R. Biol.* **2014**, *337*, 383–390.
162. Naing, A.H.; Myint, K.T.; Hwang, Y.J.; Park, I.S.; Chung, J.D.; Lim, K.B. Micropropagation and conservation of the wild medicinal orchid, *Coelogyne cristata*. *Horti. Environ. Biotechnol*. **2010**, *51*, 109–114.
163. Naing, A.H.; Park, K.I.; Chung, M.Y.; Lim, K.B.; Kim, C.K. Optimization of factors affecting efficient shoot regeneration in *Chrysanthemum* cv. Shinma. *Braz. J. Bot.* **2016**, *39*, 975–984.
164. Nasib, A.; Ali, K.; Khan, S. In vitro propagation of croton (*Codiaeum variegatum*). *Pak. J. Bot.* **2008**, *40*, 99–104.
165. Nasiruddin, K.M.; Begum, R.; Yasmin, S. Protocorm like bodies and plantlet regeneration from *Dendrobium formosum* leaf callus. *Asian J. Plant Sci.* **2003**, 2, 955–957.
166. Nayak, N.R.; Chand, P.K.; Rath, S.P.; Patnaik, S.N. Influence of some plant growth regulators on the growth and organogenesis of *Cymbidium aloifolium* (L.) Sw. seed-derived rhizomes in vitro. *In Vitro Cell. Dev. Biol.–Plant* **1998**, *34*, 185.
167. Nayak, N.R.; Sahoo, S.; Patnaik, S.; Rath, S.P. Establishment of thin cross section (TCS) culture method for rapid micropropagation of *Cymbidium aloifolium* (L.) Sw. and *Dendrobium nobile* Lindl. (Orchidaceae). *Sci. Hortic.* **2002**, *94*, 107–116.
168. Ncube, B.; Finnie, J.F.; Van Staden, J. In vitro regeneration of *Cyrtanthus* species: ornamental plants with medicinal benefits. *In Vitro Cell. Dev. Biol.–Plant* **2015**, *51*, 42–51.
169. Ng, C.Y.; Saleh, N.M. In vitro propagation of *Paphiopedilum* orchid through formation of protocorm-like bodies. *Plant Cell Tiss. Organ Cult.* **2011**, *105*, 193–202.
170. Nge, K.L.; Nwe, N.; Chandrkrachang, S.; Stevens, W.F. Chitosan as a growth stimulator in orchid tissue culture. *Plant Sci.* **2006**, *170*, 1185–1190.
171. Nikbakht, A.; Kafi, M.; Mirmasoudi, M.; Babalar M. Micropropagation of damask rose (*Rosa damascena* Mill.) cvs Azaran and Ghamsar. *Int. J. Agri. Biol.* **2005**, 7, 535–538.
172. Niknejad, A.; Kadir, M.A.; Kadzimin, S.B. *In vitro* plant regeneration from protocorms-like bodies (PLBs) and callus of *Phalaenopsis gigantea* (Epidendroideae: Orchidaceae). *Afr. J. Biotechnol.* **2011**, *10*, 11808–11816.
173. Nowakowska, K.; Pacholczak, A.; Tepper, W. The effect of selected growth regulators and culture media on regeneration of *Daphne mezereum* L. 'Alba'. *Rend. Fis. Acc. Lincei* **2019**, *30*, 197–205.
174. Onsa, R.A.H.; Abdellatif, I.A.; Osman, M.G.; Abdullah, T.L. Effect of growth regulators in *in vitro* micropropagation of *Ixora coccinea*. *Intl. J Sci.; Res. Pub.* **2018**, *8*, 144–149.
175. Oo, K.T.; Lwin, K.M.; Khai, A.A. *In vitro* micropropagation of rose (*Hybrid Rosa* spp.) through plant tissue culture technique. J. Sci. Innov. Res. **2021**, *10*, 1–4.
176. Osburn, L.D.; Yang, X.; Li, Y.; Cheng, Z.M. Micropropagation of Japanese honeysuckle (*Lonicera japonica*) and Amur honeysuckle (*L. maackii*) by shoot tip culture. *J. Environ. Hort.* **2009**, *27*, 195–199.
177. Panigrahi, J.; Dholu, P.; Shah, T.J. Gantait, S. Silver nitrate-induced in vitro shoot multiplication and precocious flowering in *Catharanthus roseus* (L.) G. Don, a rich source of terpenoid indole alkaloids. *Plant Cell Tiss. Organ Cult.* **2018**, *132*, 579–584.
178. Panigrahi, J.; Dholu, P.; Shah, T.J.; Gantait, S. Silver nitrate-induced in vitro shoot multiplication and precocious flowering in *Catharanthus roseus* (L.) G. Don, a rich source of terpenoid indole alkaloids. *Plant Cell Tiss. Organ Cult.* **2018**, *132*, 579–584.
179. Pant, B.; Chand, K.; Paudel, M.R. Joshi, P.R.; Thapa, B.B.; Park, S.Y.; Shakya, S.; Thakuri, L.S.; Rajbahak, S.; Sah, A.K.; Baniya, M.K.; Gurung, P.R.; Maharjan, L.; Rajbhandari, P. Micropropagation, antioxidant and anticancer activity of pineapple orchid: *Dendrobium densiflorum* Lindl. *J. Plant Biochem. Biotechnol.* **2022**, *31*, 399–409.
180. Pant, B.; Swar, S. Micropropagation of *Cymbidium iridioides*. *Nepal J. Sci. Technol.* **2011**, *12*, 91–96.
181. Pant, B.; Thapa, D. *In vitro* mass propagation of an epiphytic orchid, *Dendrobium primulinum* Lindl. through shoot tip culture. *Afr. J. Biotechnol.* **2012**, *11*, 9970–9974.
182. Papafotiou, M.; Skylourakis, A. *In vitro* propagation of *Callistemon citrinus*. *Acta Hortic.* **2010**, *885*, 267–270.
183. Parida, R.; Mohanty, S.; Nayak, S. *In vitro* propagation of *Hedychium coronarium* Koen. through axillary bud proliferation. *Plant Biosyst.* **2013**, *147*, 905–912.
184. Parzymies, M.; Dąbski, M.; Pogorzelec, M.; Kozak, D.; Durlak, W.; Dudkiewicz, M. Rooting of a trumpet creeper (*Campsis radicans* (L.) seem.) microshoots in presence of auxins. *Acta Sci. Pol.–Hortorum Cultus* **2014**, 13, 187–196.
185. Paudel, M.R.; Pant, B. *In vitro* micropropagation of rare orchid (*Esmeralda clarkei* Rchb. f.) from shoot tip section. *Int. J. Biol., Pharm. Allied Sci.* **2012**, *1*, 1587–1597.
186. Pawłowska, B.; Cioć, M.; Prokopiuk, B. How LED light rooting in vitro affected *Gerbera* acclimatization efficiency. *Acta Hortic.* **2018a**, *1201*, 583-590.
187. Pawłowska, B.; Żupnik, M.; Szewczyk-Taranek, B.; Cioć, M. Impact of LED light sources on morphogenesis and levels of photosynthetic pigments in *Gerbera jamesonii* grown in vitro. *Hortic. Environ. Biotechnol.* **2018b**, *59*, 115–123.
188. Pe, P.P.W.; Naing, A. H.; Soe, M.T.; Kang, H.; Park, K.I.; Kim, C.K. Establishment of meristem culture for virus-free and genetically stable production of the endangered plant *Hosta capitata*. *Sci. Hortic.* **2020**, *272*, 109591.

189. Picolotto, D.R.N.; Paiva, V.B.D.; Barros, F.D.; Padilha, D.R.C.; Cruz, A.C.F.D.; Otoni, W.C. Micropropagation of *Cyrtopodium paludicolum* (Orchidaceae) from root tip explants. *Crop Breed. Appl. Biotechnol.* **2017**, *17*, 191–197.
190. Pinheiro, M.V.M.; Schmidt, D.; Diel, M.I.; Santos, J.D.; Thiesen, L.A.; Azevedo, G.C.V.D.; Holz, E. *In vitro* propagation of alpinia cultivars in different light sources. *Ornam. Hortic.* **2019**, *25*, 49–54.
191. Plessis, H.J.D.; Nikolova, R.V.; Egan, B.A.; Kleynhans, R. Preliminary study on *in vitro* shoot culture of *Hibiscus coddii* subsp. *barnardii*, an indigenous South African flowering plant. *Ornam. Hortic.* **2021**, *27*, 408–416.
192. Podwyszyńska, M.; Kosson, R.; Treder, J. Polyamines and methyl jasmonate in bulb formation of in vitro propagated tulips. *Plant Cell Tiss. Organ Cult.* **2015**, *123*, 591–605.
193. Podwyszyńska, M.; Marasek-Ciolakowska, A. Micropropagation of tulip via somatic embryogenesis. *Agronomy* **2020**, *10*, 1857.
194. Pornpienpakdee, P.; Singhasurasak, R.; Chaiyasap, P.; Pichyangkura, R.; Bunjongrat, R.; Chadchawan, S.; Limpanavech, P. Improving the micropropagation efficiency of hybrid *Dendrobium* orchids with chitosan. *Sci. Hortic.* **2010**, **124**, 490–499.
195. Pradhan, S.; Paudel, Y.P.; Pant, B. Efficient regeneration of plants from shoot tip explants of *Dendrobium densiflorum* Lindl., a medicinal orchid. *Afr. J. Biotechnol*. **2013**, 12, 1378–1383.
196. Prasad, G.; Seal, T.; Mao, A.A.; Vijayan, D.; Lokho, A. Assessment of clonal fidelity and phytomedicinal potential in micropropagated plants of *Bulbophyllum odoratissimum*-An endangered medicinal orchid of Indo Burma megabiodiversity hotspot. *S. Afr. J. Bot.* **2021**, *141*, 487–497.
197. Ptak, A.; Bach, A. Somatic embryogenesis in tulip (*Tulipa gesneriana* L.) flower stem cultures. *In Vitro Cell. Dev. Biol.–Plant* **2007**, *43*, 35–39.
198. Pyati, A.N.; Murthy, H.N.; Hahn, E.J.; Paek, K.Y. *In vitro* propagation of *Dendrobium macrostachym* Lindl.—a threatened orchid. *Indian J. Exp. Biol.* **2002**, *40*, 620–623.
199. Qu, L.; Chen, J.; Henny, R.J.; Huang, Y.; Caldwell, R.D.; Robinson, C.A. Thidiazuron promotes adventitious shoot regeneration from pothos (*Epipremnum aureum*) leaf and petiole explants. *In Vitro Cell. Dev. Biol.–Plant* **2014**, *50*, 561–567.
200. Quynh, N.P.D.; Pha, N.T. Effect of culture media on micropropagation and *in vitro* flowering of red Eden Rose (*Rosa* 'Red Eden'). *Dong Thap Univ. J. Sci.* **2020**, *9*, 93–99.
201. Quynh, N.P.D.; Pha, N.T. Effect of culture media on micropropagation and in vitro flowering of Red Eden Rose (*Rosa* 'Red Eden'). *Dong Thap Univ. J. Sci.* **2020**, *9*, 93–99.
202. Raghuvanshi, S.; Tripathi, M.K.; Vidhya-Sankar, M.; Singh, O.P. Establishment of low-cost effective protocol for massive *in vitro* propagation in *Polianthes tuberosa* Linn. *Plant Cell Biotech. Mol. Biol.* **2013**, *14*, 49–59.
203. Rahman, M.S.; Mouri, N.J.; Nandi, N.C.; Akter, S.; Khan, M.S. *In vitro* micropropagation of *Jasminum grandiflorum* L. *Bangladesh J. Sci. Ind. Res.* **2018**, *53*, 277–282.
204. Rathod, H.P.; Pohare, M.B.; Bhor, S.A.; Jadhav, K.P.; Batule, B.S.; Shahakar, S.B.; Wagh, S.G.; Wadekar, H.B.; Kelatkar, S.K.; Kulkarni, M.R. *In vitro* micro propagation of blue passion flower (*Passiflora caerulea* L.). *Trends Biosci.* **2014**, *7*, 30793082.
205. Reddy, M.C.; Bramhachari, P.V.; Murthy, K.S.R. Optimized plant tissue culture protocol for *in vitro* morphogenesis of an endangered medicinal herb *Ceropegia ensifolia* Bedd. *Trop. Subtrop. Agroecosystems* **2015**, *18*, 95-101.
206. Restanto, D.P.; Santoso, B.; Kriswanto, B.; Supardjono, S. The application of chitosan for protocorm like bodies (PLB) induction of orchid (*Dendrobium* sp) in vitro. *Agric. Agric. Sci. Procedia* **2016**, *9*, 462-468.
207. Rittirat, S.; Kongruk, S.; Te-chato, S. Induction of protocorm-like bodies (PLBs) and plantlet regeneration from wounded protocorms of *Phalaenopsis cornucervi* (Breda) Blume & Rchb. f. *Int. J. Agric. Technol.* **2012**, *8*, 2397–2407.
208. Riva, S.S.; Islam, A.; Hoque, M.E. *In vitro* regeneration and rapid multiplication of *Dendrobium bensoniae*, an indigenous ornamental orchid. *Agriculturists*, **2016**, *14*, 24–31.
209. Rodrigues, P.H.V.; Arruda, F.; Forti, V.A. Slow-grown *in vitro* conservation of *Heliconia champneiana* cv. Splash under different light spectra. *Sci. Agric.* **2018**, *75*, 163–166.
210. Roy, A.R.; Patel, R.S.; Patel, V.V.; Sajeev, S.; Deka, B.C. Asymbiotic seed germination, mass propagation and seedling development of *Vanda coerulea* Griff ex. Lindl. (Blue Vanda): An *in vitro* protocol for an endangered orchid. *Sci. Hortic.* **2011**, *128*, 325–331.
211. Roy, J.; Banerjee, N. Induction of callus and plant regeneration from shoot-tip explants of *Dendrobium fimbriatum* Lindl. var. *oculatum* Hk. f. *Sci. Hortic.* **2003**, *97*, 333–340.
212. Saito, H.; Nakano, M. Plant regeneration from suspension cultures of *Hosta sieboldiana*. *Plant Cell Tiss. Organ Cult.* **2002**, *71*, 23–28.
213. Saleh-E-In, M.M.; Bhattacharyya, P.; Van Staden, J. Chemical composition and cytotoxic activity of the essential oil and oleoresins of in vitro micropropagated *Ansellia africana* Lindl: A vulnerable medicinal orchid of Africa. *Molecules* **2021**, *26*, 4556.
214. Sangavai, C.; Chellapandi, P. *In vitro* propagation of a tuberose plant (*Polianthes tuberosa* L.). *Electron. J. Biol.* **2008**, *4*, 98– 101.

215. Seo, S.G.; Ryu, S.H.; Zhou, Y.; Kim, S.H. Development of an efficient protocol for high-frequency regeneration system in *Hibiscus syriacus* L. *J. Plant Biotechnol.* **2017**, *44*, 164–170.
216. Shabbir, A.; Hameed, N.; Ali, A.; Bajwa, R. Effect of different cultural conditions on micropropagation of rose (*Rosa indica* L.). *Pak. J. Bot.* **2009**, *41*, 2877–2882.
217. Sharma, U.; Kataria, V.; Shekhawat, N.S. In vitro propagation, ex vitro rooting and leaf micromorphology of *Bauhinia racemosa* Lam.: a leguminous tree with medicinal values. *Physiol. Mol. Biol. Plants* **2017**, *23*, 969–977.
218. Sheela, V. L.; Sarada, S.; Anitha, S. Development of protocorm-like bodies and shoots in *Dendrobium* cv. Sonia following gamma irradiation. *J. Trop. Agric.* **2006**, *44*, 86–87.
219. Shen, X.; Kane, M. E.; Chen, J. Effects of genotype, explant source, and plant growth regulators on indirect shoot organogenesis in *Dieffenbachia* cultivars. *In Vitro Cell. Dev. Biol.–Plant* **2008**, *44*, 282–288.
220. Sherif, N.A.; Senthil Kumar, T.; Rao, M.V. DNA barcoding and genetic fidelity assessment of micropropagated *Aenhenrya rotundifolia* (Blatt.) C.S. Kumar and F.N. Rasm.: a critically endangered jewel orchid. *Physiol. Mol. Biol. Plants* **2020**, *26*, 2391–2405.
221. Shiau, Y.J.; Nalawade, S.M.; Hsia, C.N.; Mulabagal V.; Tsay, H.S. *In vitro* propagation of the Chinese medicinal plant, *Dendrobium candidum* wall. ex lindl., from axenic nodal segments. *In Vitro Cell. Dev. Biol.–Plant* **2005**, *41*, 666–670.
222. Shin, K.S.; Murthy, H.N.; Heo, J.W.; Hahn, E.J.; Paek, K.Y. The effect of light quality on the growth and development of in vitro cultured *Doritaenopsis* plants. *Acta Physiol. Plant.* **2008**, *30*, 339–343
223. Singh, K.; Madhavan, J.; Sadhukhan, R.; Chandra, S.; Rao, U.; Mandal, P.K. Production of nematode free plantlets in *Polianthes tuberosa* using in vitro culture techniques. *Hortic. Environ. Biotechnol.* **2020**, *61*, 929–937.
224. Singh, N.; Kumaria, S. A combinational phytomolecular-mediated assessment in micropropagated plantlets of *Coelogyne ovalis* Lindl.: A horticultural and medicinal orchid. *Proc. Natl. Acad. Sci., India, Sect. B Biol. Sci.* **2020**, *90*, 455–466.
225. Sinha, P.; Jahan, M.A.A. Clonal propagation of *Phalaenopsis amabilis* (L.) BL. cv. 'Golden Horizon' through *In vitro* culture of leaf segments. *Bangladesh J. Sci. Ind. Res.* **2011**, *46*, 163–168.
226. Soares, J.D.R.; Pasqual, M.; Rodrigues, F.A.; Villa, F.; Araujo, A.G.D. Silicon sources in the micropropagation of the *Cattleya* group orchid. *Acta Sci. Agron.* **2011**, *33*, 503–507.
227. Song, K.; Kim, D.H.; Sivanesan, I. Effect of plant growth regulators on micropropagation of *Hosta minor* (Baker) Nakai through shoot tip culture. *Propag. Ornam. Plants* **2020**, *20*, 57–62.
228. Sopalun, K.; Thammasiri, K.; Ishikawa, K. Micropropagation of the Thai orchid *Grammatophyllum speciosum* blume. *Plant Cell Tiss. Organ Cult.* **2010**, *101*, 143–150.
229. Souza, E.H.; Soares, T.L.; Souza, F.V.D.; Santos-Serejo, J.A. Micropropagation of *Heliconia rostrata* and *Heliconia bihai* from mature zygotic embryos. *Acta Hort.* **2008**, *865*, 315–320.
230. Sultana, K.S.; Hasan, K.M.; Hasan, K.M.; Sultana, S.; Mehraj, H.; Shimasaki, K.; Habiba, S.U. Effect of hyaluronic acid (ha) on organogenesis in protocorm-like bodies (PLBs) of *Phalaenopsis* 'Fmk02010'cultured *in vitro*. *Am. Eurasian J. Agric. Environ. Sci.* **2015a**, *15*, 1721–1724.
231. Sultana, K.S.; Hasan, K.M.; Hasan, K.M.; Sultana, S.; Mehraj, H.; Ahasan, M.; Shimasaki, K.; Habiba, S.U. Effect of two elicitors on organogenesis in protocorm-like bodies (PLBs) of *Phalaenopsis* 'Fmk02010'cultured *in vitro*. *World Appl. Sci. J.* **2015b**, *33*, 1528–1532.
232. Sunitibala Devi, Y.; Neelashree, N. Micropropagation of the monopodial orchid, *Rhynchostylis retusa* (L.). *Int. J. Life Sci.* **2018**, *6*, 181–186.
233. Surendranath, R.; Ganga, M.; Jawaharlal, M. *In vitro* propagation of tuberose. *Environ. Ecol.* **2015**, *34*, 2556–2560.
234. Tanaka, M.; Takamura, T.; Watanabe, H.; Endo, M.; Yanagi, T.; Okamoto, K. *In vitro* growth of *Cymbidium* plantlets cultured under superbright red and blue light-emitting diodes (LEDs). *J. Hortic. Sci. Biotech.* **1998**, 73, 39–44.
235. Tao, J.; Yu, L.; Kong, F.; Zhao, D. Effects of plant growth regulators on in vitro propagation of *Cymbidium faberi* Rolfe. *Afr. J. Biotechnol.* **2011**, *10*, 15639–15646.
236. Tawfik, A.A.; Ibrahim, O.H.M.; Abdul-Hafeez, E.Y.; Ibrahim, S.A. Optimizing micropropagation protocol for *Rosa hybrida* cv. Eiffel Tower with improved in vitro rooting ability. *Egypt. J. Hort.* **2018**, *45*, 323–335.
237. Thokchom, R.; Maitra, S. Micropopagation of *Anthurium andreanum* cv. Jewel from leaf explants. *J. Crop Weed* **2017**, *13*, 23– 27.
238. Thokchom, R.; Maitra, S. Micropropagation of *Anthurium andreanum* cv. Jewel from leaf explants. *J. Crop Weed* **2017**, *13*, 23-27.
239. Tibkwang, A; Junkasiraporn, S; Chotikadachanarong, K. Effects of cytokinin and sucrose on tissue culture of *Rosa chinensis* Jacq. var. *minima* Voss. *Burapha Sci. J.* **2018**, *23*, 712–721.
240. Tikendra, L.; Koijam, A.S.; Nongdam, P. Molecular markers based genetic fidelity assessment of micropropagated *Dendrobium chrysotoxum* Lindl. *Meta Gene*, **2019**, *20*, 100562.
241. Tikendra, L.; Potshangbam, A.M.; Dey, A.; Devi, T.R.; Sahoo, M.R.; Nongdam, P. RAPD, ISSR, and SCoT markers based genetic stability assessment of micropropagated *Dendrobium fimbriatum* Lindl. var. *oculatum* Hk. f.- an important endangered orchid. *Physiol. Mol. Bio.l Plants* **2021**, *27*, 341–357.

242. Timofeeva, S.N.; Elkonin, L.A.; Tyrnov, V.S. Micropropagation of *Laburnum anagyroides* Medic. through axillary shoot regeneration. *In Vitro Cell. Dev. Biol.–Plant* **2014**, *50*, 561–567.
243. Tirkey, D.S.; Nirala, D.P.; Kumari, D.P.N.P. Micropropagation from nodal explants of rose (*Rosa hybrida* L.) at different concentration of BAP (6-Benzyl Amino Purine). *Int. J. Chem. Stud.* **2019**, *SP6*, 427–430
244. Torabi-Giglou, M.; Hajieghrari, B. *In vitro* study on regeneration of *Gladiolus grandiflorus* corm calli as affected by plant growth regulators. *Pak. J. Biol. Sci.* **2008**, *1*, 1147–1154.
245. Tour, J.; Ikram, U.; Bilal, M.; Ali, M.; Zaheer, U.; Nawaz, M.A. Efficient *in vitro* propagation of *Amaranthus viridis* L. using node explants. *Acta Sci. Pol. Hortorum Cultus* **2020**, *19*, 41–51.
246. Tripathi, M.K.; Malviya, R.K.; Vidhyashankar, M.; Patel, R.P. Effect of plant growth regulators on *in vitro* morphogenesis in gladiolus (*Gladiolus hybridus* Hort.) from cultured corm slice. *Int. J. Agric. Tech.* **2017**, *13*, 583–599.
247. Ülfer, R.; Türkoğlu, N.; Özdemir, F.A. Micropropagation of *Zinnia elegans* L. *Int. J. Agri. For. Life Sci.* **2020**, *4*, 161–6.
248. Veraplakorn, V. *In vitro* micropropagation and allelopathic effect of lantana (*Lantana camara* L.). *Agric. Nat. Resour.* **2017**, *51*, 478–484.
249. Vila, I.; Sales, E.; Ollero, J.; Munoz-Bertomeu, J.; Segura, J.; Arrillaga, I. Micropropagation of oleander (*Nerium oleander* L.). *HortSci.* **2010**, *45*, 98-102.
250. Wang, G.Y.; Yuan, M.F.; Hong, Y. *In vitro* flower induction in roses. *In Vitro Cell. Dev. Biol.–Plant* **2002**, *38*, 513–518.
251. Wang, H.Y.; He, S.L.; Tanaka, M.; Van, P.T.; da Silva, J.A.T. Effect of IBA concentration, carbon source, substrate, and light source on root induction ability of tree peony (*Paeonia suffruticosa* Andr.) plantlets *in vitro*. *Europ. J. Hort. Sci.* **2012b**, *77*, 122.
252. Wang, H.Y.; He, S.L.; Tanaka, M.; Van, P.T.; da Silva, J.A.T. Effect of IBA concentration, carbon source, substrate, and light source on root induction ability of tree peony (*Paeonia suffruticosa* Andr.) plantlets *in vitro*. *Europ. J. Hort. Sci.* **2012a**, *77*, S122–S128.
253. Waseem, K.; Jilani, MS.; Jaskani, MJ.; Khan, M.S.; Kiran, M.; Khan, G.U. Significance of different plant growth regulators on the regeneration of chrysanthemum plantlets (*Dendranthema morifolium* L.) through shoot tip culture. *Pak. J. Bot.* **2011**, *43*, 1843–1848.
254. Wei, A.; Xu, Y.; Yang, N.; Jiang, L.; Hu, J.; Yang, H.; Cai, C.; Chen, J.; Chen, G.; Pan, D. *In vitro* propagation of *Codiaeum variegatum* 'Golden Queen'. *Chinese J. Trop. Crops* **2019**, *40*, 724–730.
255. Wei, X.; Chen, J.; Zhang, C.; Wang, Z. In vitro shoot culture of *Rhododendron fortunei*: An important plant for bioactive phytochemicals. *Ind. Crops Prod.* **2018**, *126*, 459–465.
256. Winhelmann, M.C.; Tedesco, M.; Lucchese, J.R.; Fior, C.S.; Schafer, G. (*In vitro* propagation of *Angelonia integerrima*. *Rodriguésia* **2019**, *70*, e02232017.
257. Wojtania, A.; Matysiak, B. *In vitro* propagation of *Rosa* 'Konstancin'(*R. rugosa* × *R. beggeriana*), a plant with high nutritional and pro-health value. *Folia Hort.* **2018**, *30*, 259–267.
258. Wongnok, A.; Piluek, C.; Techasilpitak, T.; Tantivivat, S. Effects of light emitting diodes on micropropagation of *Phalaenopsis* orchids. *Acta Hortic.* **2008**, *788*, 149–156.
259. Wu, H.C.; Lin, C.C. Red light-emitting diode light irradiation improves root and leaf formation in difficult-to-propagate *Protea cynaroides* L. plantlets in vitro. *HortSci.* **2012**, *47*, 1490–1494.
260. Xu, J.; Beleski, D.G.; Vendrame, W.A. Effects of culture methods and plant growth regulators on *in vitro* propagation of
261. *Brassavola nodosa* (L.) Lindl. hybrid. *In Vitro Cell. Dev. Biol.–Plant* **2022**, https://doi.org/10.1007/s11627-022-10276-7
262. Yalcın-Mendı, Y.; Unek, C.; Eldogan, S.; Akakacar, Y., Serce, S.; Curuk, P.; Kocaman, E. The effects of different hormones on regeneration of gazania (*Gazania rigens*). Rom. Biotechnol. Lett. **2009**, *14*, 4728–4732.
263. Yamaner, Ö.; Erdag, B. Direct shoot formation and microtuberization from aseptic seedlings of *Cyclamen* mirabile Hildebr. *Biotechnology* **2008**, *7*, 328–332.
264. Youssef, N.M.; Shaaban, S.A.; Ghareeb, Z.F.; Taha, L.S. In vitro bulb formation of direct and indirect regeneration of *Lilium orientalis* cv. "Starfighter" plants. *Bull. Natl. Res. Cent.* **2019**, *43*, 211.
265. Zahara, M.; Datta, A.; Boonkorkaew, P.; Mishra, A. The effects of different media, sucrose concentrations and natural additives on plantlet growth of *Phalaenopsis* hybrid'Pink'. Brazilian Archives of Biology and Technology **2017**, 60.
266. Zanello, C.A.; Cardoso, J.C. PLBs induction and clonal plantlet regeneration from leaf segment of commercial hybrids of *Phalaenopsis*. *J. Hortic. Sci. Biotechnol.* **2019**, *94*, 627–631.
267. Zhao, D.; Hu, G.; Chen, Z.; Shi, Y.; Zheng, L.; Tang, A.; Long, C. Micropropagation and *in vitro* flowering of *Dendrobium wangliangii*: a critically endangered medicinal orchid. *J. Med. Plants Res.* **2013**, *7*, 2098–2110.
268. Zhao, P.; Wu, F.; Feng, F.S.; Wang, W.J. Protocorm-like body (PLB) formation and plant regeneration from the callus culture of *Dendrobium candidum* Wall ex Lindl. *In Vitro Cell. Dev. Biol.–Plant* **2008**, *44*, 178–185.
269. Zielińska, S.; Piątczak, E.; Kozłowska, W.; Bohater, A.; Jezierska-Domaradzka, A.; Kolniak-Ostek, J.; Matkowski, A. LED illumination and plant growth regulators' effects on growth and phenolic acids accumulation in *Moluccella laevis* L. in vitro cultures. *Acta Physiol. Plant.* **2020**, *42*, 72.
